# A human 3′UTR clone collection to study post-transcriptional gene regulation

**DOI:** 10.1186/s12864-015-2238-1

**Published:** 2015-12-09

**Authors:** Kasuen Kotagama, Cody S. Babb, Justin M. Wolter, Ronan P. Murphy, Marco Mangone

**Affiliations:** Molecular and Cellular Biology Graduate Program, Arizona State University, Tempe, AZ USA; Virginia G. Piper Center For Personalized Diagnostics, The Biodesign Institute at Arizona State University, Tempe, AZ USA; School of Health & Human Performance, Dublin City University, Dublin, Ireland

**Keywords:** 3′UTRome, 3′UTR, 3′LIFE, miRNAs, RBPs, mRNAs, 3′ untranslated region, ORFeome, Resource

## Abstract

**Background:**

3′untranslated regions (3′UTRs) are poorly understood portions of eukaryotic mRNAs essential for post-transcriptional gene regulation. Sequence elements in 3′UTRs can be target sites for regulatory molecules such as RNA binding proteins and microRNAs (miRNAs), and these interactions can exert significant control on gene networks. However, many such interactions remain uncharacterized due to a lack of high-throughput (HT) tools to study 3′UTR biology. HT cloning efforts such as the human ORFeome exemplify the potential benefits of genomic repositories for studying human disease, especially in relation to the discovery of biomarkers and targets for therapeutic agents. Currently there are no publicly available human 3′UTR libraries. To address this we have prepared the first version of the human 3′UTRome (h3′UTRome v1) library. The h3′UTRome is produced to a single high quality standard using the same recombinational cloning technology used for the human ORFeome, enabling universal operating methods and high throughput experimentation. The library is thoroughly sequenced and annotated with simple online access to information, and made publically available through gene repositories at low cost to all scientists with minimal restriction.

**Results:**

The first release of the h3′UTRome library comprises 1,461 human 3′UTRs cloned into Gateway® entry vectors, ready for downstream analyses. It contains 3′UTRs for 985 transcription factors, 156 kinases, 171 RNA binding proteins, and 186 other genes involved in gene regulation and in disease. We demonstrate the feasibility of the h3′UTRome library by screening a panel of 87 3′UTRs for targeting by two miRNAs: *let-7c*, which is implicated in tumorigenesis, and *miR-221*, which is implicated in atherosclerosis and heart disease. The panel is enriched with genes involved in the RAS signaling pathway, putative novel targets for the two miRNAs, as well as genes implicated in tumorigenesis and heart disease.

**Conclusions:**

The h3′UTRome v1 library is a modular resource that can be utilized for high-throughput screens to identify regulatory interactions between *trans*-acting factors and 3′UTRs, Importantly, the library can be customized based on the specifications of the researcher, allowing the systematic study of human 3′UTR biology.

**Electronic supplementary material:**

The online version of this article (doi:10.1186/s12864-015-2238-1) contains supplementary material, which is available to authorized users.

## Background

3′untranslated regions (3′UTRs) are the sequences located immediately downstream of from the STOP codon of mature mRNAs. Although historical attention focused on protein coding sequences and upstream regions, 3′UTRs have recently become subject to intense study because they are targets of a variety of regulatory molecules, including RNA binding proteins (RBPs) and small non-coding RNAs (ncRNAs), that recognize small *cis*-elements present in the 3′UTRs. These *cis*-elements play critical roles in deciding the fate of the mRNA via various mechanisms, including co-transcriptional processing, modulating protein translation, mRNA localization and trafficking, and mRNA degradation and stability [1]. Disruption of these processes is known to affect diverse developmental and metabolic processes, and contributes to various diseases, including neurodegenerative diseases, diabetes, and cancer [2–5].

RBPs play a role in every aspect of mRNA biogenesis, such as stability, localization, translation and decay. The human transcriptome contains approximately ~400 proteins with distinguishable RNA binding domains [6], and their deregulation is linked to major neurodegenerative disorders, cancer, and muscular dystrophies. Compared to transcription factors, which generally bind highly specific linear DNA sequence elements, elements in 3′UTRs targeted by RBPs are generally more degenerate and difficult to identify bioinformatically because RNA is a single-stranded molecule and RBP binding is mostly dictated by local folding and polarity [6]. Consequently, RBPs have the potential to bind to multiple elements in different 3′UTRs, leading to intricate, dynamic, and mostly unknown networks of RNA-protein interactions.

3′UTRs are also targeted by a class of post-transcriptional regulators known as microRNAs (miRNAs), which are short non-coding RNAs that bind to complementary sequences in the 3′UTRs of metazoans [1]. Once bound, based on the degree of complementarity, miRNAs can induce either translational repression or mRNA degradation [7]. MiRNAs canonically recognize targets in 3′UTRs via Watson-Crick base pairing, requiring complementarity with as few as six consecutive nucleotides between the 5′end of a mature miRNAs and the 3′UTR of a target transcript [7]. However, recent evidence suggest that miRNAs do not require perfect complementarity with target 3′UTRs to induce functional translational repression, and non-canonical interactions are frequent [8]. Because miRNA target elements are degenerate and small they are difficult to detect, thus a vast majority of biologically relevant miRNA targets are still unknown. Based on bioinformatic predictions of miRNA-binding sites in 3′UTRs, it has been proposed that each miRNA controls large networks of hundreds of mRNAs [9]. However, recent analysis of the predictive performance of several of the most prominent prediction algorithms, such as TargetScan [10], PicTar [11] and DIANA-microT [12] report extremely high false negative rates [8, 13, 14]. While these algorithms are very useful for candidate gene approaches to identify miRNA targets, the extremely high error rates make high-throughput target detection challenging. Coupled with the absence of a publically available and comprehensive 3′UTR library, the field currently lacks tools to systematically study miRNA targets, which is the gold standard in miRNA biology.

Several genomic resources are currently available to systematically study gene expression and its regulation in humans. The human ORFeome for example, is a collection of over 12,000 human protein-coding genes cloned in modular vectors and optimized to study the dynamics of gene expression [15, 16]. The ORFeome has been used to characterize genome wide protein-protein interaction networks, leading to important discoveries relevant to human disease [15]. HT resources such as this can significantly advance our understanding of gene functions in multicellular organisms. Unfortunately, such a standardized HT tool to detect and study regulatory elements in 3′UTRs are not available since 3′UTR sequences are not present in the ORFeome. Some individual 3′UTR clones are available commercially, but these products have sporadic coverage, are too expensive for HT studies, use only proprietary vectors and are not compatible with the ORFeome. Furthermore, endogenous full length 3′UTRs frequently undergo alternative processing in a tissue specific fashion [17], which limits the biological relevance of experiments that use truncated or partial 3′UTRs.

To overcome this limitation, a recent study used ~240,000 short RNA sequences containing all possible 9-base nucleotide permutations immobilized on microarrays to study the binding requirements of 205 human RBPs [6]. Although this work and others highlights important binding properties of RBPs, they do not necessarily reflect biological settings, where accessory elements near binding sites that may cooperate with the RBPs targeting are not present.

Recently, our group experimented with the usage of a pilot human full length 3′UTR library to detect miRNA targets in 3′UTRs using a scalable dual-luciferase assay named Luminescent Identification of Functional Elements in 3′UTRs (3′LIFE) [8, 18]. Although we cloned and screened only ~300 query 3′UTRs, the proof of principle 3′LIFE screen was highly effective at the rapid and efficient discovery of many novel targets for two cancer relevant miRNAs, *let-7c* and *miR-10b* [8]. This pilot screen demonstrated the value of such an unbiased HT approach, and supports the need for the development of a publically available genome wide 3′UTR library.

Furthermore, there is a critical need in the field for a high-quality and standardized human 3′UTR resource, which could be widely used in the community to study miRNAs and RBPs using full length 3′UTRs in unbiased and HT experiments.

To overcome these limitations, we have developed the first publically available and high-quality human 3′UTR clone library, sequenced verified and cloned in modular vectors amenable to various downstream analyses. This resource enables the systematic study of 3′UTR biology, can be used to efficiently detect miRNA and RBP targets at high resolution, and study mRNA localization and dynamics. In the context of disease states, this library allows the study of key disease alterations in post-transcriptional processing, such as disease-specific: 1) mRNA mislocalization, 2) alternative polyadenylation, 3) altered miRNA expression, 4) mutation of RNA binding protein elements in 3′UTRs, and 5) more generally, the contribution of post-transcriptional gene regulation to gene output in disease initiation and progression.

## Results and discussion

**Fig. 1:**
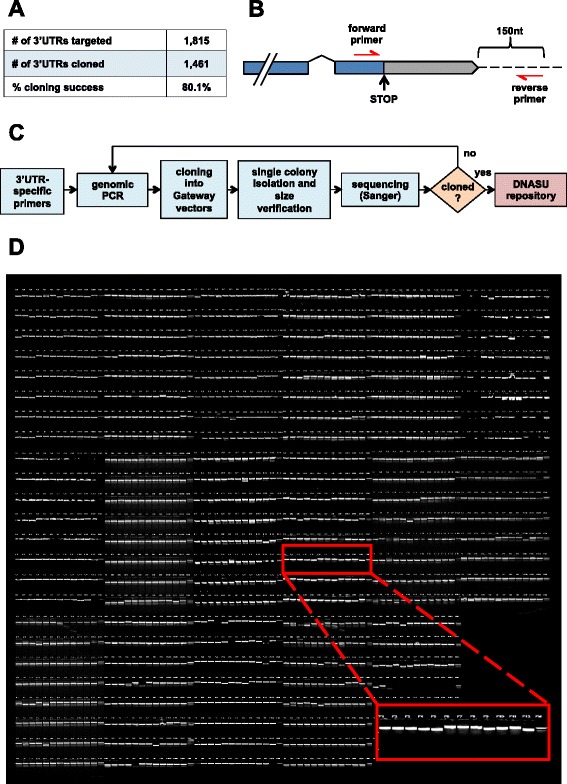
The human 3′UTRome cloning pipeline. **a** We targeted a panel of 1,815 unique human 3′UTRs and successfully cloned and sequence verified 1,461 unique 3′UTRs (80.1 % cloning success). **b** The forward primers used to amplify 3′UTR genomic loci were anchored within the last exon of each transcript, ending with the gene specific STOP codons. The reverse primers bound 150 nucleotides downstream of the annotated transcript. **c** Flow chart summarizing the cloning pipeline of the h3′UTRome v1. Genomic PCR was performed using 3′UTR specific primers and the PCR products were shuttled into Gateway® Entry vectors by recombinational cloning. Single cloned colonies were isolated and screened based on the expected 3′UTR length using PCR and gel electrophoresis. Bacterial colonies passing the screen were then re-arrayed, and the cloned 3′UTRs were sequenced using Sanger sequencing method. The sequence verified 3′UTRs were then submitted to the DNASU plasmid repository for public distribution. 3′UTRs that were not successfully cloned were subject to a second pass of cloning. **d** Electrophoretic analysis of PCR products from the complete h3′UTRome v1. The sizes of 3′UTRs from 1,461 PCR reactions were analyzed on ethidium bromide stained gels

The human 3′UTRome v1 clone collection (h3′UTRome v1) consists of 1,461 unique, cloned and sequence-validated human 3′UTRs from transcription factors, kinases and other regulatory genes (Fig. 1a). This collection is contained in modular Gateway® compatible Entry vectors, is amenable for large screens and is publically available to the community through the at the DNASU plasmid repository (https://dnasu.org/DNASU/Home.do).

### Primer design and genomic PCR

As a first release, we targeted and designed genomic primer pairs encompassing the 3′UTR regions of 1,815 human protein coding genes using the human genome release 19 (Additional file 1: Table S1) (GRCh37/hg19 Feb. 2009) [19].

The forward primers used for the genomic PCR were designed to anneal within the last exon of the target gene, ending with the gene-specific STOP codon in frame with the rest of the transcript (Fig. 1b). This expedient allowed us to increase the melting temperature of each forward primer, since the G/C content drops considerably after the STOP codon. In addition, designing the forward primer within the open reading frame provides the 3′UTR with its natural gene-specific STOP codon at its 5′end, allowing convenient in-frame integration with the human ORFeome library, which instead lacks termination codons [16] (Fig. 1b). The melting temperatures of the primers ranged from 50 to 76 °C. Given this wide range of temperature we opted for a touchdown genomic PCR approach, starting at 66 °C and decreasing by 1 °C each cycle [8]. The reverse primers were designed to target a genomic site 150 nt downstream of the annotated transcript, encompassing downstream elements that may play a role in mRNA 3′end formation (Fig. 1b). We added the Gateway® recombination elements attB2 (forward primers) and attB3 (reverse primers) to the 5′ends of the genomic primers, to facilitate the cloning into Gateway® compatible Entry vectors. A minimum of 200 ng of genomic DNA per reaction was required to obtain an enriched PCR product while minimizing non-specific amplicons, which is known to impact the recombinational cloning procedure. The complete pipeline used in this study is shown in Fig. 1c.

### Gateway® recombinational cloning

The full understanding of gene expression must consider both transcriptional and post-transcriptional regulation, requiring attention to the transcriptional promoter, the Open reading frame (ORF) and the regulatory sites within the 3′UTR.

**Fig. 2:**
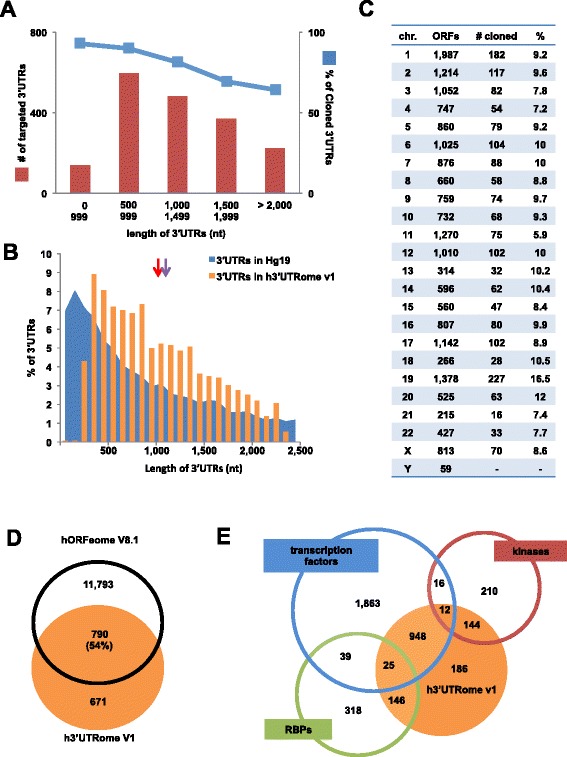
Overview of the human 3′UTRome v1 library. **a** Percentage of cloning success vs 3′UTR length. The efficiency of 3′UTR cloning (blue line) decreases ~30 % beyond the length of 1,000 nucleotides in the cloned dataset (red). The maximum cloning efficiency for amplicons smaller than 499 nt was 92.8 %, while the cloning efficiency of 3′UTRs grater than 2,000nts was 64.3 %. **b** Length of the 3′UTRs of genes in the human genome hg19 vs the h3′UTRome v1. The 3′UTRs in the h3′UTRome v1 range between 200 nt to 2,500 nt, and are enriched for longer 3′UTR isoforms. The median length of 3′UTRs in the h3′UTRome v1 is 1,159 nt (purple arrow) while in the human genome hg19 it is 1,040 nt (red arrow). **c** The h3′UTRome v1 contains 3′UTRs for 6-10 % of the ORFs within each chromosome. None of the 3′UTRs cloned in this release originates from the Y chromosome, as the source of genomic DNA used was of female origin. **d** The degree of overlap between the h3′UTRome v1 and the hORFeome V8.1. More than half of the 3′UTRs cloned in the h3′UTRome v1 contain corresponding clones present in the hORFeome V8.1. **e** The h3′UTRome v1 (orange circle) is enriched for 3′UTRs of genes involved with gene regulation as it contains predominantly 3′UTRs of transcription factors (33.9 %), kinases (40.8 %), and RNA binding proteins (32.4 %)

The human 3′UTRs in this collection were cloned into the pDONR P2r-P3 Gateway® Entry vector (Invitrogen) using BP recombinant cloning. This vector is part of the three-fragment Gateway® technology, which allows modular cloning of a given promoter, an ORFeome entry and correspondent 3′UTR to be assembled in order, into a single vector in the same reaction. This allows investigators to combine these 3′UTRs with different ORFs (which are already available in the ORFeome collection) to create both natural and novel regulatory contexts. Current protein expression vectors typically rely on viral 3′UTRs, such as the SV40 polyA, which often do not reflect natural translational levels or post-transcriptional regulation. In addition, the natural 3′UTR may contribute to proper localization and stability. This technology is also compatible with the 3′LIFE assay system and has been previously used to screen for functional miRNA targeting in 3′UTRs [8]. Successfully cloned colonies were isolated and grown in LB and analyzed by colony PCR using primers specific to the pDONR P2r-P3 backbone. The PCR amplicons were analyzed by agarose gel electrophoresis and screened based on the expected lengths of the 3′UTRs (Fig. 1d). We observed an inverse correlation between the size of the inserted 3′UTR and the BP cloning success rate (Fig. 2a). A size bias during the BP cloning reaction has been previously reported [20], with a decreased efficiency for amplicons greater than 1,000 nt and in agreement with our observations [20]. However 3′UTRs in the h3′UTRome v1 are enriched with longer 3′UTR isoforms and on average contain longer 3′UTRs than those within the human transcriptome (Fig. 2b). The nucleotide lengths of the human 3′UTR clones in this release span from 200 nt to 2,500 nt and have a median length of 1,159 nt, as opposed to the median length of 3′UTRs within the human genome, which is 1,040 nt (Fig. 2b, purple and red arrows).

The first pass of cloning produced a yield of 1,410 bacterial colonies with PCR products of the expected size. We performed a second pass on all 405 missed 3′UTRs and gained an additional 172 3′UTRs, a 12 % increase to the total number of size verified clones (Fig. 1c). The complete size-verified first release of the h3′UTRome v1 is shown in Fig. 1d.

### Sanger sequencing

A total of 1,582 size verified clones were subsequently sequenced using Sanger method using a custom primer anchored within the P2rP3 plasmid backbone. We used Perl scripts to perform BLAT alignments [21] using the Sanger trace files obtained during the sequencing. Our analysis revealed that out of the initially targeted 1,815 unique 3′UTRs, 1,461 were successfully sequence verified (~80 % success rate from genomic PCR to sequence verification).

### 3′UTRome library overview

The human 3′UTR clones contained in the h3′UTRome v1 are unbiased towards any particular regions of the genome and correspond to ~6-10 % of the total protein-coding genes present in each chromosome (Fig. 2c). The source of DNA used for the genomic PCR was GM12878, a lymphoblastoid cell line of female origin recommended as a Tier 1 cell line by the ENCODE project. Over 54 % of the 3′UTRs in the h3′UTRome v1 overlap with genes present in the hORFeome V8.1 (Fig. 2d) [16]. We targeted 971 3′UTRs of genes already present in the ORFeome and successfully cloned 790 3′UTRs (Fig. 2d). For this first release, we targeted predominantly 3′UTRs of genes previously classified as transcription factors [22, 23], kinases [24], and RBPs [25] (Fig. 2e). We targeted the 3′UTRs of this class of genes because they have widespread regulatory functions and have corresponding ORFeome clones. The h3′UTRome v1 release includes 3′UTRs for 985 transcription factors, 171 Kinases and 156 RBPs (Fig. 2e).

### Library distribution

The h3′UTRome v1 library is distributed by the DNA repository DNASU (https://dnasu.org/DNASU/Home.do), a public plasmid repository hosted at the Biodesign Institute at Arizona State University, which already distributes over 180,000 individual plasmids and full genome collections, including the human ORFeome [26]. Users can either search for a given 3′UTR clone, a plate or order the complete dataset. Many researchers are not interested in HT screens nor have the resources for large screens in their departments, but want to detect miRNA targets, mutations, or truncation of regulatory elements in the 3′UTR of their gene of interest. These researchers will be able to accelerate their research significantly because they can now order the correct ORF, 3′UTR clones and the vectors they need for their analysis at reduced cost. To simplify the ordering procedure we have given a unique ID prefix ‘HSU’ to the human 3′UTRs available with this release.

### 3′LIFE validation screen

The 3′LIFE screen is a high throughput dual luciferase assay, previously shown to detect functional repression of test 3′UTRs by query miRNAs [8, 18]. The 3′LIFE screen utilizes Gateway® cloning technology and is fully compatible with the h3′UTRome v1. In order to demonstrate the usability and functionality of this library we have selected 87 human 3′UTRs from the h3′UTRome v1 library and screened for miRNA targets of two disease-relevant miRNAs: *let-7c* and *miR-221* using the 3′LIFE assay [8, 18]. *let-7c* is a well-characterized tumor suppressor gene, is down-regulated in many cancers, and is known to target genes in the RAS pathway [27]. Conversely, *miR-221* is frequently overexpressed in breast cancer, hepatocellular carcinomas, glioblastoma and prostate cancer [28–31], and has been shown to target several tumor suppressor genes such as Kip-1 (p-27), CDKN1B, CDKN1C, PTEN, ARHI and PUMA [29, 32, 33]. In addition, *miR-221* is known to be involved with muscle damage repair and atherosclerosis [34, 35]. One of the goals of this experiment was to use this 3′UTR library to rapidly identify *bona fide* miRNA targets from false targets predicted by miRNA targeting software. These programs, such as TargetScan [10], PicTar [11] and DIANA-microT [12] are known to have high false negative rates (~43 %) [8, 13, 14] and false positive rates (~66 %) [8, 36, 37], and cannot be used alone to definitively assign targets.

These 87 human 3′UTRs were enriched with *let-7c* and *miR-221* predicted and validated targets from all three prediction softwares (9 predicted and 3 validated targets for *let-7c* and 10 predicted and 9 validated targets for *miR-221*) (Additional file 2: Table S2). For the *let-7c* screens, we also included two genes that contain validated miRNA targets identified in a previous screen [8]. In addition, since miRNAs preferentially target genes within the same regulatory pathways [8], and *let-7c* was previously shown to target the RAS family of genes [8, 27], we were interested to test if *let-7c* could also target additional 25 members of this pathway (Additional file 2: Table S2), as defined by Gene Ontology [38] and KEGG databases [39].

**Fig. 3:**
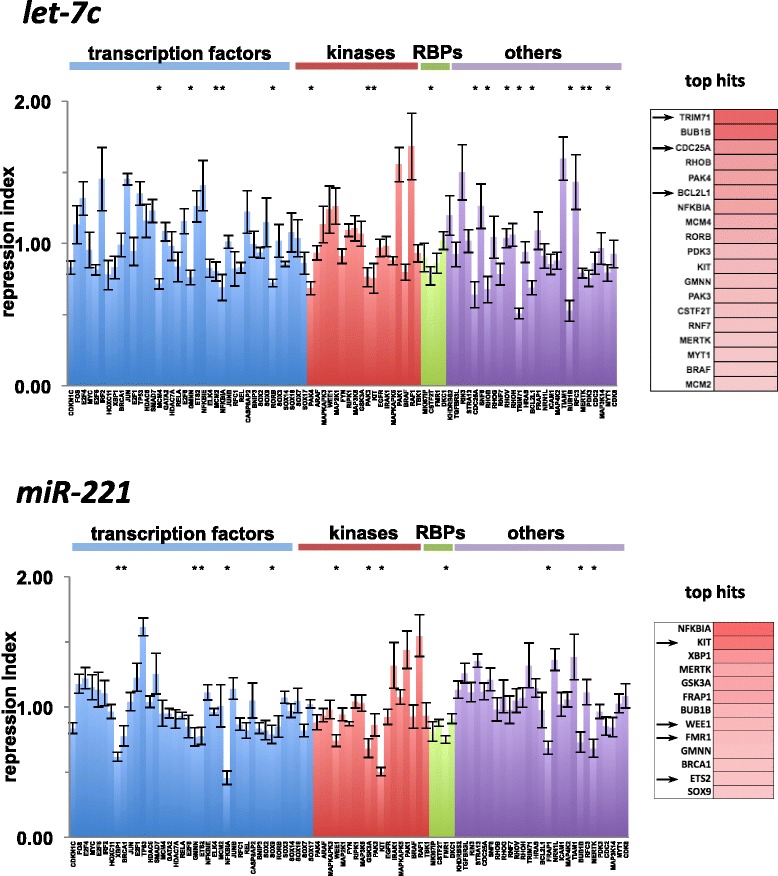
The h3′UTRome v1 as a resource to detect miRNA targets in high throughput. 3′LIFE screen performed on 87 human 3′UTRs extracted from the h3′UTRome v1, queried against two miRNAs *let-7c* and *miR-221*. The repression index was calculated for each 3′UTR and indicates a normalized ratio between Firefly and Renilla luciferases. A minimum cutoff for repression was drawn at 0.8 and the asterisks denote statistically significant repression (*p* < 0.05). The top hits for each miRNA are displayed as a heat map on the right panels. Increased intensity of color indicates greater repression. 18 top hits were identified for *let-7c* of which 3 were previously validated (as denoted by the arrows). 13 top hits were identified for *miR-221* of which 4 were previously validated (as denoted by the arrows)

We shuttled these 87 human 3′UTRs from the h3′UTRome v1 clone library into the 3′LIFE vector using LR recombination reactions. Using this custom library, we performed 435 fully automated transfections and dual luciferase experiments. The results of the screen are shown in Fig. 3. Using a cut-off for functionally repressed targets at a repression index of 0.8 and a *p*-value <0.05, we obtained 19 statistically significant hits for *let-7c,* and 13 for *miR-221* (Fig. 3).

Our results validate 4 out of 9 of the *let-7c* targets predicted by prediction softwares [10–12]. Within the predicted hits, we detected all three previously validated targets (*CDC25A, TRIM71* and BCL2L1) and an unvalidated, predicted target (RNF7) (Fig. 3 and Additional file 2: Table S2). Furthermore we detected an additional 10 novel and unpredicted targets for *let-7c* (Fig. 3 and Additional file 2: Table S2). We found that one of these novel targets *PAK3*, was predicted by the prediction algorithm miRanda [40], which takes into account non-canonical seed interactions. Of note, 3 targets within this group (*MCM2, BUB1B* and *GMNN)* were previously correlated indirectly with *let-7* expression [41].

For *miR-221*, our results validated 4 out of 10 of the *miR-221* targets predicted by the prediction software [10–12], and an additional 4 out of 9 of the targets previously validated by others (WEE1, ETS2, FMR1 and KIT) (Additional file 2: Table S2) [42–45]. Interestingly, we were unable to detect repression in 3′UTRs of 5 genes previously known to contain *miR-221* responsive elements (*CDKN1C,* FOS, IRF2, ICAM1, PAK1) [46–50]. Upon further review, we found that repression of all five targeted elements was demonstrated using truncated sections of the 3′UTR. Thus, the observation that the 3′LIFE screen did not detect these targets could be caused by the inability of these elements to recruit *miR-221* when expressed within their full length endogenous 3′UTRs, or by the presence of alternative polyadenylation events that cause the lost of these elements [8]. Two targets of *miR-221* called by the prediction software [10–12] were also not detected as hits in our assay (KHDRBS2 and RORB). We also discovered 9 novel and unpredicted targets for *miR-221* not anticipated by major prediction software [10–12], or detected by others*.* (Additional file 2: Table S2). Within this group, *FRAP1* was the only gene predicted by miRanda [40]. Perfect complementarity within the seed region is considered the canonical indicator of miRNAs targeting. Interestingly, most of these novel targets do not always contain canonical seeds. Recent studies indicate that miRNAs are also capable of recognizing non-canonical elements in target mRNAs [8, 36, 51, 52], supporting our findings.

Taken together, these experiments validate 9 out of 18 bioinformatically predicted targets [10–12] (50 % false positive rate), which is in accordance with the false positive rates of prediction algorithms reported in previous studies [8, 13, 14, 36, 37]. In previous studies we used repression data from the 3′LIFE assay to identify and validate functional miRNA binding sites [8]. With experimentally validated miRNA target sites, targeting signatures can be extrapolated to refine target predictions for specific miRNAs.

Interestingly, while the 3′LIFE assay is designed to detect repression of 3′UTRs by miRNAs, we detected several 3′UTRs that significantly enhanced the expression of the luciferase reporter gene in the presence of *let-7c* and *miR-221* (Fig. 3). Perhaps these enhancements are caused by increase stability of a given 3′UTR due to direct or indirect interactions with the query miRNAs.

The ability to systematically screen large numbers of human 3′UTRs allowed in depth analysis of high-confidence target genes regulated by different miRNAs, and may reveal novel mechanisms that miRNAs use to regulate biological processes. For example, a gene ontology analysis of the *let-7c* top hits showed an enrichment for genes involved in cell cycle checkpoint regulation, while a similar analysis for *miR-221* revealed a relationship with genes involved in negative regulation of muscle differentiation (Additional file 3: Table S3).

In addition, out of the 25 genes involved in the RAS pathway, our screen identified 7 genes directly targeted by *let-7c* (RhoB, PAK1, PAK3, BRAF, NFKBIA, BCL2L1, KIT), suggesting a role for *let-7c* in regulating this pathway.

## Conclusion

3′UTRs contain powerful regulatory elements that are critical in various biological processes, yet remain poorly characterized because due to the absence of genomic tools that allow their systematic study. In this work we have prepared the first human 3′UTR clone collection named h3′UTRome v1, which is produced to a single high quality standard. This library is compatible with the cloning technology used to produce the human ORFeome, expanding the potential of well-established operating methods for high throughput experimentation. The h3′UTRome v1 library is sequence verified, and readily available to the community with simple online access to information through the DNASU repository [53], at a low cost to all scientists with minimal restriction. In order to demonstrate its utility, we performed a screen with 87 human 3′UTRs cherry picked from the h3′UTRome v1, and rapidly identified 27 miRNA targets for two disease-relevant miRNAs, *let-7c* and *miR-221*. Within this pool, we identified 18 novel targets for these two miRNAs, which were previously uncharacterized (67 %). In addition, we were able to eliminate 9 out of 18 bioinformatically predicted targets (50 % false positive), and rapidly associate miRNA activities to biological pathways using a rapid screening technology.

The h3′UTRome v1 can be easily used in similar HT experiments to systematically study RBP targeting in 3′UTRs, mRNA localization and the role of small ncRNAs in post transcriptional gene regulation.

## Methods

### Primer design

DNA primer sequences were designed using custom Perl scripts using the annotated 3′UTR sequences in the Human Genome release 19 (Additional file 1: Table S1) (GRCh37/hg19 Feb. 2009) [19]. The forward primers were anchored upstream of the last exon of each gene and included the gene specific endogenous STOP codons in frame with the ORFome library (Fig. 1). The reverse primers were designed to target sites 150 nt downstream of the longest annotated transcript, as per the RefSeq annotation, in order to include downstream 3′end processing elements (Fig. 1). Forward and reverse primers were fused to the *attB2* (5′-GGGGACAGCTTTCTTGTACAAAGTGGAG-3′) and *attB3* (5′-GGGGACAACTTTGTATAATAAAGTTG-3′) Gateway® sequences to allow modular cloning into pDONR P2rP3 Entry vectors. The full list of primers used is available as (Additional file 1: Table S1) and through DNASU (https://dnasu.org/DNASU) [53]. The first release of the h3′UTRome V1 targeted a panel of 1,815 3′UTRs (Fig. 1) enriched for transcription factors, kinases, RNA binding protein and other regulatory genes. The length of the 3′UTRs cloned in this release ranges between 200 and 2,500 nt in length, which is larger than the average size of human 3′UTRs (Fig. 2).

### Genomic DNA

We used the NA12878 DNA sample obtained from the NIGMS Human Genetic Cell Repository at the Coriell Institute for Medical Research (Camden, New Jersey). This genomic DNA was extracted from the GM12878, a B-lymphocyte cell line of a human female subject. Once received, the genomic DNA was diluted to a concentration of 200 ng/μl, aliquoted in 96-well PCR reactions, and stored at -80 °C until use.

### Genomic touchdown PCR

The reactions were conducted using Platinum Taq polymerase (Invitrogen) in 96-well plates using 200 ng of genomic DNA per reaction. The reaction conditions were maintained as per the manufacturers protocol with changes to the annealing temperature of the reaction. The PCR conditions included 16 cycles of touchdown PCR, where the temperature of the annealing phase decreased by 1 °C per cycle, ending at a temperature of 50 °C. The reaction proceeded for 15 more cycles at an annealing temperature of 55 °C. The resulting PCR products were visualized on by electrophoresis on 96-well agarose gels and screened by size to determine successful amplicons.

### Gateway® BP recombination reaction and transformation

Site-specific DNA recombination was used to clone the human 3′UTR PCR amplicons into the Gateway® Entry vector pDONR P2r-P3 (Invitrogen), using BP Clonase II Enzyme Mix kit (Invitrogen, Carlsbad, CA) following the manufacturer’s specifications. DH5α *E. coli* cells were transformed with 1 μl of the resultant reaction mixture, and screened the following day for successful recombinants using Lysogeny Broth (LB) agar plates with Kanamycin (Kan) antibiotic.

### *3*′*UTR isolation and size screening*

In order to isolate single clonal populations, unique bacterial colonies for each 3′UTR clone were picked from the LB plates, and grown overnight in 96 deep-well plates containing LB (500 mL) with Kanamycin resistance (50 μg/mL) (total colonies picked = 1,824). The resultant bacterial growths were used as a template to perform colony PCR reactions using M13 DNA primer pairs. The amplicons were then analyzed in 96-well agarose gels and positive clones were initially screened based on their expected size (Fig. 1d). Up to three more colonies for genes that did not satisfy our quality control inspection were picked (total colonies picked = 753), and rescreened by repeating the bacterial colony PCR step. Bacterial colonies that passed the initial screen were re-arrayed and stored in glycerol stocks, while the primer pairs of the remaining genes were used in a second pass, starting at genomic PCR to capture any 3′UTR missed (Fig. 1c).

### Sanger sequencing

PCR analysis with M13 DNA primer pairs was performed for each positive 3′UTR clone, using overnight bacterial growths as a template and Phusion® Taq polymerase (New England Biolabs), as per manufacturers protocol. These PCR amplicons were then sent for sequencing at the DNA Lab, School of Life Sciences, Arizona State University, using the sequencing primer 1FP2rP3 seq (5′-GCATATGTTGTGTTTTACAGTATTATGTAG-3′) which binds ~100 nt upstream of the recombination element in the P2rP3 plasmid. 1,461 3′UTR clones successfully sequence verified and passed this step. The trace files for each 3′UTR clone successfully screened are available through DNAsu website [53]. Using custom a BioPerl script with Blat integration [21] we mapped our sequencing results to 1,461 unique 3′UTRs in the human genome.

### *3*′*LIFE screen*

The 3′LIFE assay was performed as previously described [18]. We re-arrayed bacterial colonies from a panel of human 3′UTRs from the h3′UTRome v1, and grew the plate over-night in a 96 deep-well format using 200 mL of LB in presence of kanamycin (50 μg/mL). We used 1 μl of the resultant overnight culture to perform the colony PCR with Phusion Taq polymerase (Invitrogen) as per manufacturers protocol. The amplicons from the PCR reaction were shuttled into the pLIFE-3′UTR vector (DNASU Plasmid ID: EvNO00601503) by LR recombination using LR Clonase enzyme (Invitrogen) as per manufacturers protocol. 1 μl of the resultant LR reaction mixture was transformed in DH5ɑ *E.coli* cells. The transformed cells were then plated on LB agar plates containing ampicillin (100 μg/ml), and incubated overnight at 37 °C. Single bacterial colonies were isolated and grown overnight in 500 mL of LB containing ampicillin (100 μg/mL). The resultant overnight bacterial growth was screened based on size using agarose gel electrophoresis. Bacterial colonies from wells passing the screen were frozen as glycerol stocks and also grown overnight for 96-well plasmid DNA extraction as previously described [8, 18]. In order to express *let-7c* miRNA we used the pLIFE-miR *let-7c* construct [8, 18]. The miRNA *miR-221* was extracted from human genomic DNA derived from GM12878 cells using DNA primers containing Gateway® recombination elements (forward primer – 5′-GGGGACAGCTTTCTTGTACAAAGTGGAGTTTCAACATGATGTCATGATTAAATG-3′; reverse primer- 5′-GGGGACAACTTTGTATAATAAAGTTGCACCTTATCTCTGGTTTACTAGGCTG-3′). The amplified PCR amplicon was cloned into pLIFE-miR (DNASU Plasmid ID: EvNO00601504) vector using LR Clonase II enzyme as per manufactures protocol (Invitrogen). We designed the positive and negative controls for *miR-221* targeting by introducing 22 nt long complementary sequences for the 3p arm (positive control) and 5p arm (negative control) arms of *miR-221* into the SV40 3′UTR by site directed mutagenesis (Quikchange®, Invitrogen), as per manufacturer protocol. We used the *let-7c* positive control as previously described [8]. Plasmid DNA was extracted as was previously described [8]. The 3′LIFE assay was performed as previously described [8, 18]. In brief, 87 queried human 3′UTRs + 3 controls were transfected into HEK293T cells using the 96-well Shuttle nucleofection system (Lonza). Transfected cells were cultured for 72 h and then lysed, then used to perform the dual luciferase assay. The screen was performed five times (435 reactions), and the resulting data was analyzed as previously described [8, 18]. The top hits for each miRNA were distinguished by requiring a minimum repression index of 0.8 and a *p-value* < 0.05.

## Additional files

Additional file 1: Table S1. List of primers used in the h3′UTRome v1. A unique RefSeq ID refers to each transcript. For each transcript listed the table displays its given alias, the forward and reverse primer sequences used for genomic PCR, the chromosome of origin and the length of the cloned 3′UTR. The Entrez and Ensembl gene IDs are listed where available. (PDF 391 kb) Additional file 2: Table S2. List of 3′UTRs from the h3′UTRome v1 clone library used in the 3′LIFE assay. Each 3′UTR is referred to by a unique RefSeq ID and its Alias. The 3′UTRs were queried for predicted binding by *miR-221* and *let-7c* using three prediction software; TargetScan, PicTar and DIANA tools [10–12]. Previously validated 3′UTR-miRNA interactions are listed with reference PMIDs. The direct involvement of each gene in the RAS pathway was established by using the GO [54] and KEGG [39] databases. The following column indicates if a given 3′UTR was among the top hits in the 3′LIFE screen for each miRNA. (PDF 37 kb) Additional file 3: Table S3. Gene Ontology analysis of *let-7c* and *miR-221* top hits. The top hits for *let-7c* and *miR-221* were queried for enrichments in biological processes. Only results with *p* < 0.05 are shown. The resulting biological processes are sorted based on fold enrichment. (PDF 57 kb)

## Abbreviations

3′UTRs: 3′Untranslated Regions
3′LIFE: Luminescent Identification of Functional Elements in 3′UTRs
h3′UTRome: human 3′UTRome
miRNAs: MicroRNAs
RBPs: RNA Binding Proteins
ncRNAs: non-coding RNAs
