# Supplementary material for: A human 3′UTR clone collection to study post-transcriptional gene regulation
Source: BMC Genomics. 2015 Dec 9;16:1036. doi: 10.1186/s12864-015-2238-1 (PMC4673713; doi:10.1186/s12864-015-2238-1)
Supplement: Additional file 1: Table S1. — List of primers used in the h3′UTRome v1. A unique RefSeq ID refers to each transcript. For each transcript listed the table displays its given alias, the forward and reverse primer sequences used for genomic PCR, the chromosome of origin and the length of the cloned 3′UTR. The Entrez and Ensembl gene IDs are listed where available. (PDF 391 kb) [file 12864_2015_2238_MOESM1_ESM.pdf]

RefSeq ID	Entrez gene ID	Ensembl gene ID	Alias	Chromosome	3`UTR length	Forward primer	Reverse primer	h3`UTRome v1
NM_001042416	169270	ENSG00000172748	ZNF596	8	1157	ACTCACACTAAAAAAGCAATGAATATGTAA	AACCAAGGAGGCAGAGCTTG	Y
NM_012239	23410	ENSG00000142082	SIRT3	11	1848	GTATTCCAGCTTGATGGACCAGACAAATAG	CAGGGCTGGAGGCCTCTTAG	Y
NM_001137608	654254	ENSG00000186777	ZNF732	4	604	AATAAAATTTATACTGGAGAGAAACTCTAG	TCTCTCCAGTATGAATCCTATGTTCAATTA	Y
NM_033089	85364	ENSG00000247315	ZCCHC3	20	1701	GGCGTGGCCGGGCACTAA	CAACGTAGTAGGTGCTAAGTGTTGC	Y
NM_006624	10771	ENSG00000015171	ZMYND11	10	2347	TGCCGCCGGAAAAGATGA	GTTTAAGTGAACAAATGATGTTTAACACAC	
NM_021158	57761	ENSG00000101255	TRIB3	20	1049	AGAGAAGTGGTTCTGTATGGCTAG	TGGGCTCCAGGTAATCTGTTCTCA	Y
NM_133474	170960	ENSG00000182903	ZNF721	4	1885	CATACTGGAGATAAAACCATACAAGTGTAA	CTGTTGAAAGCCACTAAAAAAGACTAGAGA	Y
NM_001895	1457	ENSG00000101266	CSNK2A1	20	1447	GCTGGCGCTCAGCAGTAA	TCTCTGCTCTGCCCGGTAC	Y
NM_005343	3265	ENSG00000174775	HRAS	11	574	AAGTGTGTGCTCTCCTGA	GAGCACCACAGCCCAGAC	Y
NM_006883	6473	ENSG00000185960	SHOX	Y	762	TGGAGTATAATGGCATGA	GAGGGTAAGAAGGAATGAGAGATGGAG	Y
NM_004609	6939	ENSG00000125878	TCF15	20	778	CGAGGGCCACGGAGATGA	TGAATGGGACATCACAGGAAACTG	Y
NM_005632	6650	ENSG00000103326	SOLH	16	1300	GGGCCCCGACCGCTGTGA	CTTGTGAGTCACCCCACCAC	
NM_006651	10815	ENSG00000168993	CPLX1	4	1724	CAGGACATGCTCAAGAAGTAG	CTGGACGGCCTAGGGAAAATG	
NM_002819	5725	ENSG00000011304	PTBP1	19	1681	TCCTTCTCCAAGTCCACCATCTAG	TTGGTCATTCGCTCTGGCC	Y
NM_023924	65980	ENSG00000028310	BRD9	5	913	GCCTCTGCCAAGACCTAA	ACGACAGCTGGGCCAGAG	Y
NM_001166260	NULL	NULL	TRIP13	5	647	TTGACCCAAATTGATCAGATTAAAAGGTAA	AGTGGGTGGGATAGGAGAGAAGT	Y
NM_004237	9319	ENSG00000071539	TRIP13	5	1126	AAGAAGCTTGCAGCTTACATCTGA	GGCCACTGGCACCTTCCC	Y
NM_001347	1609	ENSG00000145214	DGKQ	4	1916	GAGAGCGATCCTAGGTAG	TCTCCAACCTGAAGACACGTTG	Y
NM_021951	1761	ENSG00000137090	DMRT1	9	1131	ATCGAGGAGGACGAGTGA	GCATTTAATAAAAAACAAGATGGTCAGGTT	
NM_005224	1820	ENSG00000116017	ARID3A	19	918	ACATCTACCTCAAATAACTCGTTGCCTTAA	AGTTCATTGGCTCCGCGG	
NM_021240	58524	ENSG00000064218	DMRT3	9	907	GACTCTAGAACACTCAACACATCATCTTAA	ACCCTAAAACTTTGCCATTCATATTTCTCA	
NM_014587	30812	ENSG00000005513	SOX8	16	1773	ACCCTGACCAGGCCCTGA	GGCTCGCGGCCGCCCTCC	
NM_001130865	10655	ENSG00000173253	DMRT2	9	1960	TATTATGTGTACATAATGAACCATCTTTAG	TGTGCCCTCCACAATGCAAAACTAA	
NM_001328	1487	ENSG00000159692	CTBP1	4	980	GCCAGTGACCAGTTGTAG	GACCACTGCCCAACCTGC	Y
NM_198253	7015	LRG_343	TERT	5	736	TTCAAGACCATCCTGGACTGA	TGACCACAACCCCATTCACTCATAG	Y
NM_001280	1153	ENSG00000099622	CIRBP	19	909	TCCTCAGCTACACACAACGAGTAA	GTCTGCTGGGCCTCCCTTTCT	Y
NM_033260	94234	ENSG00000164379	FOXQ1	6	1022	GAGACGCTCCTAGCCTGA	GGAAATCTGCAAGCCCATTCTATAATACCT	Y
NM_016823	1398	ENSG00000167193	CRK	17	2340	CCCGATGAGGACTTCAGCTGA	AGGAATCCTTAGGACTTGAGTAGCG	Y
NM_001452	2295	ENSG00000137273	FOXF2	6	918	ATTAAGCCCTGCGTCATGTGA	TGTTACTCAAGGCACAGACCCC	Y
NM_018959	26528	ENSG00000071626	DAZAP1	19	951	CACCCCTACCGACGCTAG	GCCTCTGCCCTGAAGCCC	Y
NM_001037125	64718	ENSG00000059145	UNKL	16	717	CCTTCCTCCACCGCCTGA	CGAGAGTTTTGATCCCACAATTTAAAACTC	
NM_003957	9024	ENSG00000174672	BRSK2	11	1880	CTGATACCAGGAATTATCCCGAAAAGTTAA	CCGCCCGGAATCTCACCC	Y
NM_203304	399664	ENSG00000181588	RKHD1	19	1074	CAGGCCATTCATATCTTTTCCTAG	CCCAATTCCTTCGTCTGGAACAAG	
NM_003926	NULL	NULL	MBD3	19	1841	GAGATGGAGCACGTCTAG	CCTAAGCCTGACTCTTGGGTTGT	Y
NM_001453	2296	ENSG00000054598	FOXC1	6	1967	GTCTACGACTGTAGCAAGTTTTGA	CCAGCGAGATTTAAACGGGGC	Y
NM_006527	7884	ENSG00000163950	SLBP	4	977	AGAGACTTCTCAGCCATGAGCTAA	AATTAAAAAAATAAAGAAGGCCCTTTACCC	Y
NM_016358	50805	ENSG00000113430	IRX4	5	722	AAACCCTTCTGCGCCTGA	GGCTTAAAGTCTTGGGTTGAACCA	Y
NM_006497	3090	ENSG00000177374	HIC1	17	1032	GACCGTTTCTCTCCCACCTAG	CCTCTTGCTGATCTGTTTCTGGGA	Y
NM_001319	1455	ENSG00000133275	CSNK1G2	19	1314	CTGCAGCGACACAAGTGA	CCTTACCTTCCGCCTCATCAGT	Y
NM_017572	2872	ENSG00000099875	MKNK2	19	448	AGAACCGTTACTGTGAATGAGTGA	CATCCAGGCATCCTGGAGCA	Y
NM_002744	5590	ENSG00000067606	PRKCZ	1	566	ACCGAGGAGTCGGTGTGA	AAACAATTCTTGATTGCGGTGTGTC	Y
NM_000296	5310	ENSG00000008710	PKD1	16	1197	CACCCCAGCAGCACTTAG	CCAGCGGAAGCGCCTCAT	Y
NM_003070	6595	ENSG00000080503	SMARCA2	9	1064	AGTGGGACGGATGATGAGTGA	GACCACTTCAAGGAGACTGGCTC	Y
NM_005170	430	ENSG00000183734	ASCL2	11	1433	TGGTTAGGGGGCTACTGA	CAAGGTGTCCCAATGGCTCC	
NM_006711	10921	ENSG00000205937	RNPS1	16	1045	TCCAACTCCTCCCGATAA	GATAAAACCATCTCCTTCTATTTGTCCACC	
NM_004729	9189	ENSG00000214717	ZBED1	X	2401	AGGGACAGCAGCTTCCTGTAG	AGAGGAGCACAGAACTGACCG	Y
NM_001037283	8662	ENSG00000106263	EIF3S9	7	1425	CTCGGGAATCAGGAGTGA	ACACAGGGTGGCCGAGTG	Y
NM_003091	6628	ENSG00000125835	SNRPB	20	474	CTTCTTACTCTTACTTCAGGCCTTCTTTGA	GTGTATGAGGATGTACAGATGCAAATGG	Y
NM_001010926	388585	ENSG00000197921	HES5	1	904	CTCTGGCGGCCCTGGTGA	GACCCCACCCTTCTTCCG	Y
NM_024325	79175	ENSG00000088876	ZNF343	20	1520	AGACACCAGAGGACACACTGA	ACTGTGAATTTGAATTCCTTGCACCCTT	Y
NM_001185010	6047	ENSG00000063978	RNF4	4	2291	GCCTCCGTGATTCCCTGA	AGCCTCTCACATGCTTGCTG	Y
NM_001110514	57593	ENSG00000088881	EBF4	20	1309	CAGGGCCTGGCATACTCCTAA	CCAACAGTCCCCAAGAACAGAC	Y
NM_001134222	153572	ENSG00000170561	IRX2	5	1579	GTCCAGCCCTACCTATAG	TTTTTTTTTTTTTTTCAATGCTCAGGAGAG	
NM_016333	23524	ENSG00000167978	SRRM2	16	725	ATCTTCTCTTGCAGGTCTCCATAA	AAGCTGGAGCCAGTGTCCT	Y
NM_001102651	115196	ENSG00000172006	ZNF554	19	2063	TACCAGAACAGACATCTTATTGGATATTAG	TAATGCACATCAGCACTCCTGGTC	Y
NM_000076	1028	LRG_533	CDKN1C	11	966	CGCAAGAGGCTGCGGTGA	TGGGCCAGGCCCAGCCAG	Y
NM_018463	NULL	NULL	ITFG2	12	1058	CTCCAGGATCCCACCTAG	AGTATAATTGAGAGGAATTTGAAAGCAATG	
NM_021217	58492	ENSG00000175691	ZNF77	19	451	ACACATGCTGGAGCGTGA	AAAAAAAACAAGAAAGTAGGAATCAAAAAC	Y
NM_001243089	2305	ENSG00000111206	FOXM1	12	1137	CAGTTTATTCCTGAGCTACAGTAG	TGTGTGCCTGTGCAAGTGCT	Y
NM_001144761	7089	ENSG00000065717	TLE2	19	466	GTCCTCAGTCCTGAGTTGTGA	AGAGTGGCTCTCACATACCAGG	

NM_003324	7289	ENSG00000078246	TULP3	12	1876	AGTAAGCTGGCGTGTGAATGA	TCCTGGCCTCAGGTGATCC	
NM_001160408	7289	ENSG00000078246	TULP3	12	594	CCACATCAGATAAGCATGTGA	TCCTGGCCTCAGGTGATCC	Y
NM_003804	8737	ENSG00000137275	RIPK1	6	2028	TTGATTTACGTCAGCCAGAACTAA	AGGGAGGTGTTCTTACCATCAGCA	Y
NM_002919	5991	ENSG00000080298	RFX3	9	1019	GACTGTGGAGTTATTGCAAGAGTTCCTTAA	GAAGTGGAAAAGATGAATATTTCAACTGAC	Y
NM_198087	7752	ENSG00000010539	ZNF200	16	1747	AAGACCCGAAAGCAGAAGTAA	TTGTGGAGGATTTGTTGTTTTTAAATTACG	Y
NM_001198536	4210	LRG_190	MEFV	16	1843	CCTAGGTATTCAAATTTTCTTTGCAGTTAA	ACCCGCAGATTTTCCCATACAAAC	Y
NM_005741	10127	ENSG00000006194	ZNF263	16	1081	ATGAGTCATCAGAGAACTCACACAGGTTAG	GCCTCTGCCTGTCTGCTG	Y
NM_153028	7627	ENSG00000162086	ZNF75A	16	887	AGACACCAGAAACTCCACCTGTGA	CTCTCATTCTTAACAACAAAGAAAAGCCAA	Y
NM_001130520	7748	ENSG00000005801	ZNF195	11	1371	CTGACTGTACATGAAAGCATTCATACTTGA	GTCTCTAGCAGGTTCTGGAAGGG	Y
NM_017810	54925	ENSG00000140987	ZNF434	16	947	CAGGAAGGAAGAGATGCGTTATGA	CTCTTCCCCATTTCCTACCCCTATTC	Y
NM_139214	90655	ENSG00000176679	TGIF2LY	Y	419	GCCAGAAGCTCACCGGAATAG	TGCTGCAGTGTTCATTATGATGGCA	Y
NM_174886	7050	ENSG00000177426	TGIF1	18	648	CTTCAGGCAAAACTTACAGCTTAA	GGCTTGTTGTCCACAGGTTTTCAAA	Y
NM_003450	7727	ENSG00000103343	ZNF174	16	625	CTTCACCATGGGGACTAA	TTTTCTTTTTGTAAAAGCACCATCTAACTA	Y
NM_006339	10362	ENSG00000064961	HMG20B	19	742	TTCAGCGAGCACCTGTGA	GCTCCCGTCTCCCTATCTGAGAAA	Y
NM_031965	83903	ENSG00000177602	GSG2	17	546	TGCCAGCACAGTCTGTTTAAGTAA	GAGGCTGAGGTAGGAGAATTGCTTG	Y
NM_001810	1059	ENSG00000125817	CENPB	20	1013	GGTCTTGGACATCAAAGCTGA	CCCAAGCTTGAGGTCTCCTCC	Y
NM_032294	84254	ENSG00000004660	CAMKK1	17	2068	GACGAGGCTGCATCCTGA	CCCTCCGCCAAGACCACTT	Y
NM_001665	391	ENSG00000177105	RHOG	11	765	TCCTGCATCCTCTTGTGA	GGGAGCCTAGAGCTTTGTTAGGGA	Y
NM_001145640	23217	ENSG00000105278	ZFR2	19	822	TGGGACCCTGCCTGGTGA	AGTCAGCTATGTATGGCCAAATGC	Y
NM_020873	57633	ENSG00000175928	LRRN1	3	1091	GACACATCCAGAAGCTATTACATGTGGTAA	CACTGCCCATTAGGTGCTAGTCAGT	Y
NM_001348	1613	ENSG00000167657	DAPK3	19	827	GAGTGCGGGCTGCGCTAG	GTCTGCGAGGACCCCCGG	Y
NM_032607	84699	ENSG00000060566	CREB3L3	19	1262	GCGGGAGACGAGCTGTGA	TGCCCGGCGCCTCTCCCT	Y
NM_003223	7023	ENSG00000090447	TFAP4	16	1049	GACGGGGAGCTTCCCTGA	TTTTTCATCGCAACACCACACACAA	Y
NM_145291	166793	ENSG00000168826	ZNF509	4	650	AATGAAAACGAGTTAGACCAGTGA	GAGTCAGGCAGTTCTGACCACTG	Y
NM_032575	84662	ENSG00000126603	GLIS2	16	2253	CCGGCTGTGGTGAACTGA	CAACCTTCCAGTTCCTGCTCTAAG	Y
NM_014520	10514	ENSG00000132382	MYBBP1A	17	699	AAGGCAGGGAAGCCCTGA	CAGGTGGAATACTCACCCACCAA	
NM_001199054	29965	ENSG00000089486	C16orf5	16	2076	TACAAGCGCCTGTGCTAA	ACTCTAACCGTCTTGCAAAGATCTTTAAAG	
NM_170663	50488	ENSG00000141503	MINK1	17	954	AACTGCATCATGAACTGGTGA	CTAGGTTTTCTTGGCCCAGCACT	Y
NM_002448	4487	ENSG00000163132	MSX1	4	970	AGCATGTACCACCTGACATAG	AGACACCCCCCTCTATCCCT	
NM_001171167	23125	ENSG00000108509	CAMTA2	17	894	CGCCTCGTGCAGTCTTAA	GGGAAGGGCGGAATGCCTTTAA	Y
NM_001171166	23125	ENSG00000108509	CAMTA2	17	944	CCGGGACTGGCCACATGA	GGGAAGGGCGGAATGCCTTTAA	Y
NM_003670	8553	ENSG00000134107	BHLHB2	3	1668	CCCCCTTTAAACTTAGAAACCAAAGACTAA	CTAAGCTTATTCTGATTGAATACAAAGCAC	
NM_004505	9098	ENSG00000129204	USP6	17	2231	GATTACGAAAAGTACTCTATGTTACAGTAA	GTACTGGTCTGTAGCCCAGGAGT	
NM_004972	3717	LRG_612	JAK2	9	1572	GATCAAATAAGGGATAACATGGCTGGATGA	GATTTCTGCATCCAGAACTATTAAAATGCT	Y
NM_003409	7541	ENSG00000198081	ZFP161	18	2019	GAGACGATAGCCTGTAGCTAG	GGTTACCACCATGACTATGTGGTTTCAAAG	Y
NM_014649	9667	ENSG00000130254	SAFB2	19	424	TTCACCCGCCGCTACTAA	GACAACTCAGCAGGATGATGCAG	Y
NM_001199573	10346	ENSG00000132274	TRIM22	11	1395	TGCCCACCGAGCTCCTGA	CAAGTGTTTCATTACGAGAAACAGATCTGA	
NM_173464	91133	ENSG00000154655	L3MBTL4	18	1694	CAAGAAGTCAGGGGATGA	GAAATATATCCCAAGAAAGACAGCACTCCA	Y
NM_134433	5990	ENSG00000087903	RFX2	19	1851	TCCCTGCAGGGCATCTAG	GGGGGACAGGGCCTAGAG	Y
NM_001145547	84991	ENSG00000134453	RBM17	10	2083	TTGGATTTGGCAGAACAAGTTTGA	GTTGCAGTGAGCCCAGATCAT	Y
NM_032286	84246	ENSG00000133398	MED10	5	757	GATCACCCGCCTTCTTAA	AAAAATTGGAGTTCTGCATTTTGCTTTAGC	Y
NM_002096	2962	ENSG00000125651	GTF2F1	19	892	CACTTCTCCCTCAAGGAGTGA	TGCAATCCAGCCTGGTGAACA	Y
NM_003685	8570	ENSG00000088247	KHSRP	19	1184	TCTCCGCAGGCTCAATGA	AGGGGAAGACAGCAGTGCT	
NM_001257326	322	ENSG00000166313	APBB1	11	589	GGGGCCCATACCCCATGA	AAAGATAAGAGACTTGGACTCCAGACC	Y
NM_001257324	NULL	NULL	APBB1	11	633	TGTGGGTGGGGTCACTGA	CTGGTAGAAGAAGTATTGGAAGGCAGA	Y
NM_016060	51003	ENSG00000108590	MED31	17	1334	CAACAGCAAAATAACACATCGGGAAAATGA	CCATGAACCTTTAGTTTGCCTGTAATACAT	Y
NM_001014795	3611	LRG_444	ILK	11	437	CCTATCCTTGAGAAGATGCAGGACAAGTAG	CCCTCAGGACCGCAAGTACA	Y
NM_001273	1108	ENSG00000111642	CHD4	12	774	GTAGCCCAGCAGCAGTGA	GCAAGGCACATACTGCACCC	Y
NM_138350	90326	ENSG00000041988	THAP3	1	1678	TGGTTGAGTGAGGAGTGA	CCTTCTGATCACCTGCGTCTGTC	Y
NM_004240	9322	ENSG00000125733	TRIP10	19	487	CTCCGAGTCACGCTCAATTGA	ATGACATAGGCCAACTTCCAGAAAG	Y
NM_001200	650	ENSG00000125845	BMP2	20	1354	GGTTGTGGGTGTCGCTAG	TGACTTATCAAATAACTTGCCTGCAATTTT	
NM_001127586	51147	ENSG00000111653	ING4	12	988	TTGCCTGTGTGGGGCTGA	CCCTGGTCTGAGGCCTAC	Y
NM_133476	171017	ENSG00000126746	ZNF384	12	1417	CACCTGGCCAGCTCTTAG	TGACTGGAGAATTGCACAAATGGG	Y
NM_002824	5763	ENSG00000159335	PTMS	12	690	AATGGGGCATCGGCGTGA	ATCTTGTCTTCTTGAGTCGTGGGT	Y
NM_181844	255877	ENSG00000161940	BCL6B	17	2203	ATTCTCGGGGGGCCCTAG	GGGCAGGGAAGGGGACAA	Y
NM_001195563	23261	ENSG00000171735	CAMTA1	1	462	TCCTTTGTTTTGCAGAGATCATGA	TGGTTTGGGTGGTTTGGGAGTA	Y
NM_001242701	23261	ENSG00000171735	CAMTA1	1	656	TGGAGCCATGAGGGCTGA	CCCTGAACTAGGCTTCCAGTTACTTCA	Y
NM_013250	7762	ENSG00000149054	ZNF215	11	1696	CAAAAACTGCATACTCGAGATAAGTCCTGA	TACCTCAAGAATTTTTTACATTCAGAGTGC	Y
NM_013249	7761	ENSG00000149050	ZNF214	11	724	CACAATAATCATAGAAGAGGAAACTTATAA	ATTATTAAGCGTGGAAAACCGGAAAGG	Y
NM_001940	1822	ENSG00000111676	ATN1	12	721	GAAAGCGACAAGCCACTGTAG	CAATTCTTCCCAGCCCCCACA	Y
NM_001144831	11331	ENSG00000215021	PHB2	12	513	AGCCTCATCAAGGGTAAGAAATGA	CGGAGCCCAGGCCTTGCT	Y

NM_014469	27288	ENSG00000170748	HNRNPG-T	11	1029	GGCCGGAGCAGATACTAA	GCTCTTTCAGGGATTCTCTGACCTTT	Y
NM_015982	51087	ENSG00000006047	YBX2	17	716	ACCACCATCCTGGAGTGA	AACCCATGATAGGGGCACTCTTC	Y
NM_001251902	8711	ENSG00000174292	TNK1	17	869	GTCCTGGCCAGGCCCTGA	GGTCTCTACCTCCCCATCAACA	Y
NM_031480	83732	ENSG00000124784	RIOK1	6	776	GCCAAGACGAAAAAAGGCAAATAG	CTGCTTTTTACTTACTTTGGTTAATGTCTT	Y
NM_004860	9513	LRG_285	FXR2	17	780	TTGGGTAGTATGGTGAATGGGGTTTCATAA	TTCCCAGAAGTAAGACCATCTTTGGG	Y
NM_001126115	7157	LRG_321	TP53	17	1387	GGGCCTGACTCAGACTGA	CAGACTGACCCAGTCTCCAG	Y
NM_001126118	7157	LRG_321	TP53	17	1387	GGGCCTGACTCAGACTGA	CAGACTGACCCAGTCTCCAG	Y
NM_001005271	1107	ENSG00000170004	CHD3	17	1352	GTGATCTGTATAGACGACTGA	AACCTCAGTTTTAGTGAATAATCTAAGAGA	Y
NM_001718	654	ENSG00000153162	BMP6	6	1565	GTAAGAGCTTGTGGATGCCACTAA	ACTTTGCATCCAACACTCTTCACC	Y
NM_024865	79923	ENSG00000111704	NANOG	12	1144	ATGCAACCTGAAGACGTGTGA	AGGTTCAAGCGATTCTCCTGC	Y
NM_145185	5609	ENSG00000076984	MAP2K7	19	2227	CTGCCCTTCTTCAGGTAG	AGCTGCTTGATACCCATCGG	
NM_001195259	100507588	ENSG00000260001	TGFBR3L	19	1151	CCCAGGAGGTCCCAGTGA	TGGGCATATGGGCCCCGT	Y
NM_001165967	84667	ENSG00000179111	HES7	17	1161	TGGAGACCTTGGCCCTGA	AAGTAAGGAATGGGGCAAATCTTAAGAGTG	Y
NM_014232	6844	ENSG00000220205	VAMP2	17	1888	CCTCTCTCCACAGTTTACTTCAGCACTTAA	CTCTTGCAAAGTCCAGTCCATTACCAAT	
NM_002051	2625	ENSG00000107485	GATA3	10	1358	GTCACCGCCATGGGTTAG	GACGTTGACTCTCTGGGGTTAGC	Y
NM_001080826	157285	ENSG00000275342	SGK223	8	598	CTCCTGCAGCTTCTGTGA	AGAGAAACAGGGAAGATGAATTGGTAC	Y
NM_002315	4004	ENSG00000166407	LMO1	11	492	ACCTTTGAATCCCAAGTTCAGTAA	TTCCTCCCAGCTTCTTCCCCTAAA	Y
NM_001270428	4004	ENSG00000166407	LMO1	11	492	ACCTTTGAATCCCAAGTTCAGTAA	TTCCTCCCAGCTTCTTCCCCTAAA	Y
NM_030906	65975	ENSG00000130413	STK33	11	819	TCCAGAACCAAAAAGAAACTCTAA	AGAGAAATGAGGTATGGTCCCCTGTTT	Y
NM_031203	4670	ENSG00000099783	HNRPM	19	444	ATTGACGTTCGAATTGATAGAAACGCTTAA	AGAGTCTCCCCCCATCTGTG	Y
NM_014583	29995	ENSG00000071282	LMCD1	3	702	AAGTCCAAACGCTCCTGA	CGGGGGTTCTGTCCCAAAG	Y
NM_002166	3398	ENSG00000115738	ID2	2	1734	AAAGCACTGTGTGGCTGA	GGGGTGCTGCTGGTGGGG	
NM_018942	3166	ENSG00000215612	HMX1	4	826	ATGCCTGGCCTGGTGTGA	CCTCCGACTAGGGTCTGCC	Y
NM_001428	2023	ENSG00000074800	ENO1	1	540	AGAAACCCCTTGGCCAAGTAA	CAAGGACCCAGGAAAATGACCC	Y
NM_001042476	23589	ENSG00000153048	CARHSP1	16	2399	GGACATGTCATCAGCTCCTAG	AAACAGATGATCCCATCTTCTTATTCACCA	Y
NM_004426	1911	ENSG00000111752	PHC1	12	2185	GCCAAGATAAATGTCCTCAAGGAGACCTAA	CAGTTGTAAGTCAGAGTTAATATAATAGGG	Y
NM_015453	25917	ENSG00000134077	THUMPD3	3	2283	ACTCTTTGGCAATGCAAAGAATGA	CACTCTGGTACCCTCTAGTATTGGGAGATA	
NM_001202411	84527	ENSG00000188321	ZNF559	19	2397	ATTTTCCATGTGTAACAGAATTCAGAGTAA	CTGGGATTACAGGAATGAGCCACT	
NM_177990	57144	ENSG00000101349	PAK7	20	2252	ATGAGACAATACAGGCATCACTGA	ATGGATATATTTGTCTTTACGTCATGTCAT	
NM_001271314	10781	ENSG00000174652	ZNF266	19	1029	GATGAGAGACTGTCAGCATAA	TCACAGGGCCTATCACCACAAGA	Y
NM_003442	7702	ENSG00000166478	ZNF143	11	1091	ACGCCAGGGTTGGATGATTAA	TAAAAATATATGGAAAAGCACTTACTCTGT	Y
NM_001143976	7465	ENSG00000166483	WEE1	11	1344	TCTGTCAGCCTTACTATATACTGA	CTAGCAAGTCACATGCATAACATGCAAT	Y
NM_024106	79088	ENSG00000130818	ZNF426	19	555	TCACTTCGAAGACATGAACAAATTCACTAG	GCAGATGGCCATCATCTGATGACTT	Y
NM_001008727	7675	ENSG00000197961	ZNF121	19	504	TTACTTACTAAACATTTAAAAACACACTGA	CTCGAGACCAGCCTGGCC	
NM_006354	10474	ENSG00000171148	TADA3L	3	567	AAGCTGCTGGATGGGTAG	AGAAAGAGGTACTCAGAAAGTTCTCCCT	Y
NM_133480	10474	ENSG00000171148	TADA3L	3	1076	CACGACCTGCTGAGGTGA	GCAGAACAAGATGGATGGGAGTTGA	Y
NM_198182	29841	ENSG00000134317	GRHL1	2	1757	CTCACCCTGACGGAGATCTAA	ATCATTTCAGGCATCAGTAAGGAAAAGTTA	Y
NM_001379	1786	LRG_362	DNMT1	19	501	GAGGAAGCTGCTAAGGACTAG	AGTTCTGAACAAACACAGACAGACACA	Y
NM_001570	3656	ENSG00000134070	IRAK2	3	1695	GAGCTCTTTGGCCCCTGA	AACTCCCTAACTGGGCCCAAGA	Y
NM_000201	3383	ENSG00000090339	ICAM1	19	1508	CAAGCCACGCCTCCCTGA	CATAGAAAGTCCGATTTGGACCAGAAC	Y
NM_003220	7020	ENSG00000137203	TFAP2A	6	1914	AAAGAGGAGAAGCACAGAAAGTGA	GGGGGGCGGGGGGGGGG	
NM_001103167	100125288	ENSG00000220201	ZGLP1	19	468	GACCCCATTCAGGAAGGTTAA	TGTGGGGGCTGCTGCCTG	Y
NM_133452	125950	ENSG00000161847	RAVER1	19	1421	CTGAAGCGGAAGAGGATTTTCTAA	ATCAATGGAACATAAGCGCAGATGTTT	Y
NM_003331	7297	LRG_121	TYK2	19	486	GTGTTCAGCGTGTGCTGA	GAGCAAGACTCTGTCTCAAAAAGAAAAAAA	Y
NM_031439	83595	ENSG00000171056	SOX7	8	2150	ACGTACTACAACAGCTACAGTGTGTCATAG	AACCATCCCTACCAGATAACAACTTTTTAA	Y
NM_012289	9817	ENSG00000079999	KEAP1	19	712	CAGCAGAACTGTACCTGTTGA	CTTGCCTACGGGGAGAAAGATG	Y
NM_001800	1032	ENSG00000129355	CDKN2D	19	776	ATGGTGGCCCCGCTGTGA	CCCAGCTCACTCACCCTG	Y
NM_004565	5195	ENSG00000142655	PEX14	1	951	GAGAGTGAGCGGGACTAG	CACTGGACCTGAACAAGGCCT	Y
NM_017766	54897	ENSG00000130940	CASZ1	1	771	CTCCAGTTCCAGGAGAAGTGA	ACTCTGACCAGGCCAGGGAA	Y
NM_005906	4117	ENSG00000111837	MAK	6	1909	TATGGAGGCCACCGGTAG	ACCCACCTATGACTCCCAGAGTT	Y
NM_001145426	8531	ENSG00000060138	CSDA	12	2391	CAGAGCAGTGCTGAGTAA	TTTTTTATCATCTCAAGCTTATATGCCTAA	
NM_004752	9247	ENSG00000124827	GCM2	6	952	TTCTTTACCTACAACAATGAGGATTTTTGA	ACAACTGTTTCCCAGGTCATAATATGAACA	
NM_000246	4261	LRG_49	CIITA	16	1860	TCACGGATCAGCCTGAGATGA	CCAACAGCCAGCATTGCCT	Y
NM_199141	10498	ENSG00000142453	CARM1	19	1195	ATGCACTACGGGAGCTAG	CCCTGGAACAGGAGGGAGA	Y
NM_004958	2475	LRG_734	FRAP1	1	1134	CTTAGGTGCCCTTTCTGGTAA	TGGGAACAGTCTGAGGAAAGGGA	Y
NM_004850	9475	ENSG00000134318	ROCK2	2	1965	TAACTTTATTTTTCCTTTCTTTTCAGCTAA	TAGGCCAGTAGCTACTCTTCAGTTTC	
NM_001715	640	ENSG00000136573	BLK	8	671	TACGAGCTGCAGCCCTAG	CGGATTTATGAGGCCACAGC	Y
NM_198256	1876	ENSG00000169016	E2F6	2	2282	AGTGAAGAATTGCTTGAAGTAAGCAACTGA	GGATCGCTCAAGGTTTCTGAAGGAA	Y
NM_138783	115950	ENSG00000161914	ZNF653	19	435	GATCACAAGCCCACCTGA	GCCTCACCCCATCCTGTG	Y
NM_002052	2626	ENSG00000136574	GATA4	8	1705	GGGGACATAATCACTGCGTAA	ACCCACTGCTACCTAAAGAGGGA	Y
NM_001136472	9516	LRG_253	LITAF	16	2095	GGCACCTACAAGCGTTTGTAG	TGACATTATTTCCTTGCATCTAGCAAAATG	

NM_148903	9687	ENSG00000196208	GREB1	2	957	TCGCCCCGGAGCTTATGA	GCTTCACTGGGTGCTGCC	Y
NM_033090	9687	ENSG00000196208	GREB1	2	902	GCACATCAAATACGAAATCCGGACGTATAA	AAGTTGGAAAGACAATTTTTCAAAAAGCCA	Y
NM_078628	10943	ENSG00000005302	MSL3L1	X	2348	TGCCAGGCATGGTGCTGA	AATACACTAAAAACTACTGGATTGAACTGT	Y
NM_001193270	10943	ENSG00000005302	MSL3	X	854	AACCCCCGGGCAATTTATTAA	CTGCCAAGTGAGGCAAAACCAAA	Y
NM_001080493	55552	ENSG00000197933	HSZFP36	19	616	AAAAGGACTCACTGGAAAGATACTCTCTAA	TCCTGCTTGTCTATAATGCTACAACACATG	Y
NM_144680	7566	ENSG00000154957	ZNF18	17	692	TTAGGAAAGAAGCCCTTTCAATAG	ACATTAGCCTAGGACTTTCCTATTACTGTT	Y
NM_152355	126068	ENSG00000197044	ZNF441	19	2352	TCCTTTCATAAACATGAAATGACTCACTAG	ATTAGGTTGTGCAAAAATAACCATACATGC	
NM_152262	90594	ENSG00000171291	ZNF439	19	1102	AAGAATGCACTCTGGAGAAAGACCTTATAA	GCAAAAGGAAAAAAAAAGAAAAATTACAGT	Y
NM_021915	7620	ENSG00000198429	ZNF69	19	1320	AATTTTTCTTATCTTTTTTTTTTTCCAGAA	CTTAAAGGAAGTGACTGAATTGTTCTAAGC	Y
NM_002114	3096	ENSG00000095951	HIVEP1	6	718	AGGCTTGTGATAGCAACCTGA	TTTCACATGGTAGAAAGGCGAGTAAAGTCT	
NM_001203250	7568	ENSG00000132010	ZNF20	19	1414	CATGAAAGAACTCATACCATTAATAGATGA	GAGATCGAGTCACTGCACTCCA	Y
NM_145233	90589	ENSG00000257591	ZNF625	19	583	CAAGGCGAGAAGATTGCTTAA	GAGTCTCACTCTGTCACCCAG	Y
NM_003437	7695	ENSG00000196646	ZNF136	19	1428	CCTTATAAATGCATGTGGGAAAGCCTTTAA	TGGGCAACAGAGCACAGAG	Y
NM_016264	51710	ENSG00000197857	ZNF44	19	777	AAAAGGACACACTGGAAGGATATTCTATAA	ACAAAAGTAATCTGAGTATTGAAACAAGAA	Y
NM_145276	147837	ENSG00000188868	ZNF563	19	1284	GAAAAGACTCACTGGAGAGAAACAATATGA	GACAGGGTTTTGCCACATTACCCA	Y
NM_030824	79973	ENSG00000198342	ZNF442	19	510	AAAAGGACTCACTGGAGAGATACTCTATAA	AAAAGAAACTGTAACATTCTAGGTGACATG	Y
NM_014160	23609	ENSG00000075975	MKRN2	3	1638	CTTTCTGGAGTGGAATCATCAGAACCCTAA	TCTTCCATCATGCTGAATTTTGTCTTCCAG	Y
NM_002880	5894	LRG_413	RAF1	3	1093	AGGCTGCCTGTCTTCTAG	AAAGAGTACAAAGGTTAAATTACAAATTCA	Y
NM_144976	163050	ENSG00000249709	ZNF564	19	1256	CAACCTTCGAATACCTGTGAAAATGAATAG	GGGGTTTCATCATGTTAGCCAGGAT	Y
NM_153498	57118	ENSG00000183049	CAMK1D	10	1027	CACTCTGGAAGCAAGTGA	CAAAGTCCCCCGCAGAGAAGA	Y
NM_021643	28951	ENSG00000071575	TRIB2	2	2118	GAAGAGAACTTGGACCCTTTCTTTAACTGA	ACGATGAAAGGGAGATTCCATGTCTTAAAA	Y
NM_002229	3726	ENSG00000171223	JUNB	19	676	AAGGGACACGCCTTCTGA	CCTGGGCTGGGACCACAA	Y
NM_006563	10661	ENSG00000105610	KLF1	19	642	ATGAAGCGCCACCTTTGA	CAGTGCCCAAGGGGGCTT	Y
NM_004343	811	ENSG00000179218	CALR	19	757	GCCAAGGACGAGCTGTAG	TAAATAAGGGTCCCTGGAATCTTGTGGA	Y
NM_001008213	10133	ENSG00000123240	OPTN	10	1590	CAGATTCACGTGATGGATTGCATCATTTAA	TACTGTTGAATTTGTATCATGTGTTCTCTT	Y
NM_005583	4066	ENSG00000104903	LYL1	19	471	AGCCCAGAGGTGCGGTGA	CCTCTGTCCAAGCGCCGG	Y
NM_001178	406	ENSG00000133794	ARNTL	11	689	TTGCCATGGCCGCTGTAA	TTATGCAAGCCCAGAATCGTTATGAAG	Y
NM_001136041	79885	ENSG00000163517	HDAC11	3	1921	CCCCCTGCAGTGCCCTGA	TTACTTTGGGATCTGGCAGAGCT	
NM_023072	65249	ENSG00000132003	ZSWIM4	19	1360	CGGGAGCGTTTTGGTTGA	CTGGGCAACAGAGAAAGACCAAAAAAAAAA	Y
NM_002918	5989	ENSG00000132005	RFX1	19	1345	GCGCTGCCCTCCAGCTAA	CCAGGCCGGCAACCTGGC	
NM_001135610	7799	ENSG00000116731	PRDM2	1	2075	GAAGTGACCTGGAATCAGTGA	TACCAGATGGGACGTTTCCAGAGT	Y
NM_002730	5566	ENSG00000072062	PRKACA	19	1597	AAGTGTGGCAAGGAGTTTTCTGAGTTTTAG	GGAGTGCCGGAATTTCAGCTAAAC	
NM_014463	27258	ENSG00000170860	LSM3	3	433	CCACTGAGAGTTGGCTGA	CAGTAGGTGCACTGCAAATGTGT	Y
NM_007118	7204	ENSG00000038382	TRIO	5	1107	AGGCTTCTGCCTAGAGTTTGA	TCAGAGTATGAAATCACTTTTACAGTCCTC	Y
NM_002582	5073	ENSG00000140694	PARN	16	1197	TTTGAAGTTCCTGACACATGGTAA	CAGTTAGGGGTAAAAAGAAGAAGAAAAAAA	Y
NM_018179	55729	ENSG00000171681	ATF7IP	12	870	GATGTGATCTCTTCTACCCAGAGCAGTTAA	GATTGTGCCACCGCACTCTAG	Y
NM_004963	2984	ENSG00000070019	GUCY2C	12	663	GACAAGGAGAGCACCTATTTTTAA	TAACCTTCAAGGTTCTCACCCTTGA	Y
NM_032433	84449	ENSG00000160961	ZNF333	19	1815	TGTGGGCCCCTTGCTAATTAA	CTAAATTAAAAGATGCAAAGCTTCAGGCAG	Y
NM_001029954	441549	ENSG00000185267	ARMETL1	10	908	CCCAAAACAGAGCTCTGA	GAAATGTTTTTTCAGTATTCCCCAGCTGTA	Y
NM_001193425	79723	ENSG00000152455	SUV39H2	10	1973	TGCAGAGGTTACCTCAACTGA	CACTGGGGGCAACCTGTAAAC	Y
NM_018427	54700	ENSG00000085721	RRN3	16	1902	CAACCCAGTCCCCTCTGA	GAAACCATCCTCACCCCGG	Y
NM_014299	23476	ENSG00000141867	BRD4	19	2424	CTCTACGTAGGTCCTGCCTAA	GCCTAAAGGGGCTGGTGGTA	Y
NM_001128217	11168	ENSG00000164985	PSIP1	9	1633	TCTCTGAAGGATTCTACACTAGATAACTAG	TATCATCTTGAGCTTACTGTTTAGTTACCC	Y
NM_004973	3720	ENSG00000008083	JARID2	6	1971	AGTGCTTCGAGCTCATCATGA	ATGGAAACCATTAGGTTAGGCTGAAG	Y
NM_001721	660	ENSG00000102010	BMX	X	562	CTTCGGGAAAAAGACAAGCATTGA	GGAGTTTGCTAAAAATACAAATTCACAGGC	Y
NM_144770	54033	ENSG00000185272	RBM11	21	1259	CGAAAGTCTAAGAAGAAGAAAAGATACTAG	ATTTGGGTGGGGACACAGAC	Y
NM_006311	9611	ENSG00000141027	NCOR1	17	2382	CTGTCGGATAGTGATGACTGA	CAGAAACTATACGGTTGACCTTCAAATAAT	Y
NM_001190438	9611	ENSG00000141027	NCOR1	17	465	AACAGAGAGTGGGAAGGTAGGTAG	CAAGGTGAAGGCTTAGCCAGC	Y
NM_005378	4613	ENSG00000134323	MYCN	2	1090	ATTGAACACGCTCGGACTTGCTAG	GGGACAATGACTCATGCCCC	Y
NM_181716	201161	ENSG00000166582	PRR6	17	463	AAGACCATCAAGAACATGTCTAAAGAGTGA	CTCGATCAACCCTCCGGAGTAG	Y
NM_015001	23013	ENSG00000065526	SPEN	1	1208	ATTGTCATTGCCTCCGTGTGA	GGACTGACTGACTAGGAGGCAGAA	Y
NM_016270	10365	ENSG00000127528	KLF2	19	677	CACATGAAACGGCACATGTAG	GCAGCTGGCAGTCCCGGT	Y
NM_004431	1969	ENSG00000142627	EPHA2	1	1058	GTGGGGATCCCCATCTGA	CCCACCCCTGCTCCCAGG	
NM_020653	57336	ENSG00000141040	ZNF287	17	1719	GTTCATACAGGTGCCAAACATCGTAATTAA	ACCCAGTTATCATTGTTTAATGATTACTGC	Y
NM_001290	9079	ENSG00000169744	LDB2	4	1281	CCCCAGGCTTCCCAATAA	TTGGTAACCAGTGCACAGTGAACTTA	Y
NM_006387	10523	ENSG00000085872	CHERP	19	1450	GCCAGGGACGAGTGTAAGTAG	CGAGGGTAAGGACATGGACC	Y
NM_001190811	1618	ENSG00000092345	DAZL	3	2046	CGGGCAATGCTTAAATCTGTTTGA	TTGTACCTGAATTTGCTTTTATGACCTAAC	
NM_004831	9441	ENSG00000105085	CRSP7	19	1300	CCTTATGTCTGCTTGGACTGA	AGCAGGCTAACAGGTGGACT	Y
NM_003092	6629	ENSG00000125870	SNRPB2	20	947	GCTATGAAGATCACCTATGCCAAGAAATAA	AACAAATTGTCCACTTCTGGAGATACC	Y
NM_001159767	28969	ENSG00000136261	BZW2	7	615	GAATCCGAATCGGAAGGTGAGGAAAATTAA	CTGGGATTACAGGTGTGAGCAGAATAG	Y
NM_002893	5931	ENSG00000102054	RBBP7	X	563	CTGGAGGGACAAGGATCTTAA	ATATGTATGATAAACTTAACAGCTTCTGTG	Y

NM_001271606	10409	ENSG00000176788	BASP1	5	1125	ACCGTAACCGTGAAAGAGTGA	ATTACAGGCGTGATCCACCGT	Y
NM_003380	7431	ENSG00000026025	VIM	10	502	ACTTCTCAGCATCACGATGACCTTGAATAA	GTGAGGTCTATCAAAAATGACAAAATTAAG	Y
NM_001143942	221662	ENSG00000112183	RBM24	6	1929	CAGACAGACCGAATGCAATAG	CTTACTTTAAAGGCACAAAACCAGGACATC	Y
NM_004587	6238	ENSG00000125844	RRBP1	20	684	GAGGGCACCTCTGTCTGA	CCACGCTACGCGGAGCTG	
NM_005124	9972	ENSG00000124789	NUP153	6	1239	ATAAAGACTGCTGTTAGACGCAGGAAATAA	CAGATACTTTCAGATACTTTCCCTTTCTCT	
NM_025205	80306	ENSG00000118579	MED28	4	919	CCTCTGAAGCCAACGTGA	AAATGTTTCCCCTCCCAAAATGTAGAACAA	Y
NM_001005291	6720	ENSG00000072310	SREBF1	17	1453	ACTGTCACTTCCAGCTAG	GTAAACGTGTGTATTATATCTGGCCTCGTT	
NM_002478	4654	ENSG00000129152	MYOD1	11	803	CCGATATACCAGGTGCTCTGA	TTTGCACCCCTCCTTCCTTC	Y
NM_000215	3718	LRG_77	JAK3	19	2139	CACCACTCCCTGTCCTTTTCATAG	AGCCCCACCCAGTGGTCC	
NM_021220	58495	ENSG00000125850	OVOL2	20	664	GAGGAGGAGGAGAGGAAGTGA	GTGACAGAGTGAGAACTTGTCTCAAAAACA	Y
NM_003472	7913	ENSG00000124795	DEK	6	1730	GTTTCTCTTTCATTACAGCTAATTTCTTGA	TGAAGTCACTCCAAGAAAATGGTCCATTAC	Y
NM_006089	10389	ENSG00000102098	SCML2	X	2118	GAAAAGCTTAAAGAAGGAAAATACAGTTAA	TTAGCAGTAGAATCTGGGCAGGTTTTAAAA	Y
NM_015016	23031	ENSG00000099308	MAST3	19	2143	CCCACCGGAAGAGACTGA	ATCAGGAAGAATCTCAACAGCGAACT	
NM_001142307	2965	ENSG00000110768	GTF2H1	11	1354	CGTCTGATGAAGAAAACGTGA	GCATGTGCTACCATGCCTG	Y
NM_001131010	6304	ENSG00000182568	SATB1	3	1709	ACAGACATTAATACTGATTTGAAAGACTGA	TGAAAAAAAAAAGCTAAGGGGTTTTTATAC	Y
NM_005354	3727	ENSG00000130522	JUND	19	868	CAGGTGCCCGCGTACTGA	CGGGCCGCTCTCAGACAC	
NM_001252129	25804	ENSG00000130520	LSM4	19	1414	CAGGCGGGCAAACAGTGA	CATCAATCAAGTGCCGCTGTG	Y
NM_006292	7251	ENSG00000074319	TSG101	11	415	CTCAGTGACCTCTACTGA	TGGTATATCCCCATGTTAATTCACTTTATA	Y
NM_005406	6093	ENSG00000067900	ROCK1	18	1822	TTTTCCCCCTCCCTCAACAGTTAA	GAAAAGATAGTCAAAAGAAAGGTTGTCAGA	Y
NM_006532	8178	ENSG00000105656	ELL	19	2269	CTGCAGGCTTGGCCCTAG	CACACAACATCCAACACACACCG	Y
NM_001204148	9734	ENSG00000048052	HDAC9	7	2496	CCAGGATTTGTAATTAAAGTCATTATCTGA	AATGGTATTAGATGTAACTGCTCAAAATGT	
NM_182543	221078	ENSG00000241058	NSUN6	10	778	GCAAAATTTGTAAAATGCAAAAGCACATAG	TCTTTCTCACCAAGCATTTTCTAATAACTG	Y
NM_002911	5976	ENSG00000005007	UPF1	19	2247	GGGCTGTCCCAGTATTAA	GGCAGGGGTCGCCCTTCA	Y
NM_000474	7291	ENSG00000122691	TWIST1	7	1425	ATGTCCGCGTCCCACTAG	CTTGTGCCTGTCAGTAGCTGCTTTATT	Y
NM_003476	8048	LRG_440	CSRP3	11	820	CTTACACAACAAGTGGAAAAGAAAGAATGA	TTGAGCTGCTGAAACTTGCTGAAGA	Y
NM_006938	6632	ENSG00000167088	SNRPD1	18	1289	GGGGGTCCTAGGCGATAA	ATTTAAGCCACCCACATCCTCAGTT	Y
NM_024680	79733	ENSG00000129173	E2F8	11	775	CTGGAAGTCTCAACAGAGGATGTCCATTAA	AATGGGGGACAGAATGTAGAAACATTCTAT	Y
NM_003325	7290	ENSG00000100084	HIRA	22	919	GACATCCTGAGGGACAAGTAG	ACGCAGTATTAACCATGACAGGG	
NM_001001671	389840	ENSG00000180815	MAP3K15	X	871	GAAACCAAAGACAAGGCTTGA	AAGAACAATTCCTTGTATGCCTGTTTCC	Y
NM_145260	130497	ENSG00000143867	OSR1	2	970	GAGCTCAAAACCTCCAAGATCAAATGCTAA	GACGTTGCTCCACACATCTCAG	Y
NM_025245	80714	ENSG00000105717	PBX4	19	498	GGCAGCATCAACTCCAGTACATCTAATTAA	AAAATCAGAACAGAATGATACGGTCCCA	Y
NM_005992	6899	LRG_226	TBX1	22	747	CACTGCAAGGACACTTGA	CCCTACAGGGGAGGTCCCA	Y
NM_005257	2627	ENSG00000141448	GATA6	18	1885	GCCCTGGCCCTGGCCTGA	CACTGGCTATGGACACTGTCCC	
NM_021030	7561	ENSG00000105708	ZNF14	19	1060	GAAAGGACTCATATGGGAGAGAAAGTCTAA	AGTAAAAAATGCTGTTGCTGAATTGGAAAT	
NM_001099269	440515	ENSG00000081665	ZNF506	19	2021	GTTCCTCAACCCTTAATAAGCATAAGATAA	AAATGAAATTAAGTTCACACATCATCTAAC	
NM_021047	56242	ENSG00000256771	ZNF253	19	917	GTTCCTCCACTTTTAATTAGCATAAGATAA	TCTTTGTATTTGCACAATTTTTCTCAAGGA	Y
NM_031218	81931	ENSG00000184635	ZNF93	19	935	GAGATAATTCATACTGGGGAGAAACCCTAG	AAGTGTATAAAACCATACATATACATGTAT	
NM_033196	91120	ENSG00000197124	ZNF682	19	1767	TTTAATCACTGCTCAAACCTTACTACGTAA	TTAAAGTCACTAAGGATAAGAATCACTAGA	Y
NM_003884	8850	ENSG00000114166	PCAF	3	2059	ATTAAGGAAGCTGGATTAATTGACAAGTGA	TGATGACCTATGGATTTTTTATGAAACCTC	Y
NM_007138	7643	ENSG00000213988	ZNF90	19	1988	TACATAGTGAAGAACATGGCAAATCTTTGA	GGCAGGAGAATCGCTTGAACTTG	Y
NM_002997	6382	ENSG00000115884	SDC1	2	2149	AAACAGGAGGAATTCTATGCCTGA	CTCTGTCTTCCACGACAGGAGG	Y
NM_006410	10553	ENSG00000109854	HTATIP2	11	758	GGCTCTCTCAAGCCATGA	TTTATAAGAGGGTTAAAGCACAGATAACAC	Y
NM_002894	5932	ENSG00000101773	RBBP8	18	426	AAAGGCAAGGAGCAGAAGACATAG	AATACTAGACACTGATATGACCTAGAGAAG	Y
NM_004040	388	ENSG00000143878	RHOB	2	1564	AACTGCTGCAAGGTGCTATGA	ATGCTCTCAAAGGGACTTCATCCTCATTTA	Y
NM_182700	221833	ENSG00000164651	SP8	7	2195	CGCAACGGCCTAGAGTGA	AGCTTTGCCAAATAGATCTTTCCATCTAAC	Y
NM_145297	199777	ENSG00000188171	ZNF626	19	873	GAGAGCCAAAGTCCTCTTCATGACATATAA	TATGAAAACTTGAAGAGGTGTCTGCTCC	
NM_152584	86614	ENSG00000172468	HSFY1	Y	509	CATTTAAAAGATGAGTCGAATTACTCATAA	TACACATATGCTGAGTAGGGCACTCTAATC	
NM_001641	328	ENSG00000100823	APEX1	14	444	ATCACCCTATACCTAGCACTGTGA	GCTCAGTCTTTGGGGGAGGTTA	Y
NM_032409	65018	ENSG00000158828	PINK1	1	1000	TGGAGGGCAGCCCTGTGA	CTGTCTTTCTTTTAACTGTGAAATGATGGT	Y
NM_003831	8780	ENSG00000101782	RIOK3	18	2036	GGAGACCCACCACTACTATATGATGAATAG	GGCCATTTGCGATTGAAATCTAACAATGAG	Y
NM_001256172	7639	ENSG00000278091	ZNF85	19	1322	ACCCAGTCTGGAATGCAGTAG	ATTTATTATCAAAATAATGGTGCATAAAAC	Y
NM_002756	5606	ENSG00000034152	MAP2K3	17	1189	GAGATCCTGGGAGAAGACTCATAG	GATGTAGAAGGGCCTGGTAGGTT	Y
NM_001888	1428	ENSG00000103316	CRYM	16	443	ATCTATGATTCCTGGTCATCTGGTAAATAA	AGTGGATCTTAGAGTATGTGAGTTGTAACT	Y
NM_006767	8216	ENSG00000099949	LZTR1	22	1869	CTGGGCGCCGACATCTGA	CCTTCAACTCCCAGGAACCTGG	Y
NM_033176	644524	ENSG00000125816	NKX2-4	20	724	TATGGCAGGACGTGGTGA	GTATACAACCTTTCTTATGGTGAGCTGCAT	Y
NM_021269	7562	ENSG00000182141	ZNF708	19	2293	ACCAAAGAGAAACCCTACAAATGTAAATAA	ACATTACCAAATAGTATATTCTTACTATCA	
NM_014572	26524	ENSG00000150457	LATS2	13	2013	CAGCCTGTGTACGTGTAG	TTTTCCACACCCCAAGACATTCCT	Y
NM_001102454	51222	ENSG00000165804	ZNF219	14	670	GGGGGGCAAGAACGGTAG	TTAAATGCTAATTCTTGGGCCCCATCA	Y
NM_145326	284443	ENSG00000196268	ZNF493	19	1451	CTCTATCTCCTGACCTCGTGA	TTGTTCCACTGCACACTCCAG	
NM_001077443	3183	ENSG00000092199	HNRPC	14	2266	AATGGCGAGGATGACTCTTAA	ACCTGCTTTCCTAGAATAAAATGTATAGTT	
NM_005870	10284	ENSG00000150459	SAP18	13	1923	GGGCGCATGAGACCATATTAA	CGTCCCAGTTTTTGTACACCGTGAAA	Y

NM_001170629	57680	ENSG00000100888	CHD8	14	599	TCCAGTGAAGATGCTGATGACTGA	TACCCTTTAGAGGAGTACAGATGACTTGTC	Y
NM_001146706	8284	ENSG00000012817	KDM5D	Y	760	TACTCTGATGAGCAACAGTTGTGA	ACCTTGGCCTAAGTCTCATGTATGTTTTCT	
NM_018411	55806	ENSG00000168453	HR	8	1461	ACATTACAGGAGGCCAAATAG	AAGGACAAAACACAGCAATCCAGCTAT	
NM_005407	6297	ENSG00000165821	SALL2	14	1786	GATGACCCCACGATCCCATGA	AGGACAAGGCTGAATAAAGTAAATATTTCA	Y
NM_033468	113835	ENSG00000197134	ZNF257	19	1839	TCCTCAAACCTTACTAAACATAATTCATAA	CCTGGCCTCAATTGATCCGC	Y
NM_001802	NULL	NULL	CDR2	16	1209	AAATACCGATCACTCTCCTCTCATTCTTAA	AAGTTACACAAATCCAAGGATTCCCTGTTT	Y
NM_001001411	163223	ENSG00000196109	ZNF676	19	1029	AAGAAAATTCATACTGGAGAGAATCCCTAA	CTTTATTTAATATGAACTCTCTGGTGTTGA	Y
NM_022160	63951	ENSG00000176399	DMRTA1	9	742	TACTTCAGACCAAATCAGGACAATCCGTAA	CGATAGTGGCCAGGAGATGG	Y
NM_021784	3170	ENSG00000125798	FOXA2	20	1026	CCCATTATGAACTCCTCTTAA	TTGAAGTGGATTTAGAGAAATAAGATGGAT	
NM_005180	648	ENSG00000168283	BMI1	10	2123	TCAGCAACTTCTTCTGGTTGA	TTAAAGTTTTTAGCCTTTTAAAAATATTTT	
NM_015461	25925	ENSG00000198795	ZNF521	18	968	AATCATACAATGACCCAACACAGCAGTTAG	ATGCATTTGGCCCAGTGTGAAAA	Y
NM_020526	2046	ENSG00000070886	EPHA8	1	2033	CCCCGCCGGCACCTCTGA	GCACTAATCCTACCTCCCCAGACA	Y
NM_004442	2048	ENSG00000133216	EPHB2	1	1940	CAGATTCAGTCTGTGGAGGTTTGA	GGAGCTGTCGTTCTCCATGTGTTTT	Y
NM_007342	11097	ENSG00000136243	NUPL2	7	446	CCTCCACCTCTGGAACTTCTAAATGTTTAA	TCTTTGAGAGAATCCTGAACATGAAGTCAT	Y
NM_006547	10643	ENSG00000136231	IGF2BP3	7	2333	CCTCAGTCAAGACGGAAGTAA	TTCTTAGTTCATATGCAAGAGTATTATCAA	
NM_018107	55147	ENSG00000100461	RBM23	14	1265	TTCCCATTTACCTCTTCTTTCCACAGGTAA	CAGTGGGTAGCTGGGATGGAGATA	Y
NM_015013	23028	ENSG00000004487	AOF2	1	507	CAGTCCCCAAGCATGTGA	ACCATTTTTCTCTTTTATATGAACATCTTC	Y
NM_178161	256297	ENSG00000168267	PTF1A	10	526	CCACCATTTGAGTTTGTGTCCTGA	GAGGTCTAATCCTCTAACCTACCCAC	Y
NM_020227	56979	ENSG00000164256	PRDM9	5	1004	GTCTGCAGGGAGGATGAGTAA	TTTAGATTGTACGACCCGACCCCA	Y
NM_003430	7644	ENSG00000167232	ZNF91	19	1887	GCTAACACAGTGAAACCCCTTCTCTACTAA	CACCTAAGATTCATCTTAGACTAACAGATG	
NM_005637	6760	ENSG00000141380	SS18	18	2281	CAGTATGGAAATTACCAGCAGTGA	AACTTGGGCATGGCAAGAGAATTTTGATAA	
NM_005826	10236	ENSG00000125944	HNRPR	1	851	ACTTATGGGCAACAGTGGAAGTAG	TCAGTCTAATATCCACAACACATGTTGGAA	Y
NM_001261414	6660	ENSG00000134532	SOX5	12	2102	ATTGCAGGACAAGCCAACTGA	CAACACTAGCAAAAAGAGGCAGTTCAAATG	Y
NM_020834	57594	ENSG00000215271	HOMEZ	14	2120	GATGATGATGATGTGATCATACAAGACTGA	CACTTTTACAAGCTCTAGAATGGTGGAATG	Y
NM_005664	7681	ENSG00000179455	MKRN3	15	893	CTGGAAGAATATTTCAATTTGATTCTGTAG	AGATCTGCATCTGTCACATTTGGC	Y
NM_138330	171392	ENSG00000197372	ZNF675	19	500	GGAGAGAAACTACAGAACTGGAATGTGTGA	GGGTTTTTCTCTAGTACAAAATGTACATGA	Y
NM_002167	3399	ENSG00000117318	ID3	1	1210	AAAAGGAGCTTTTGCCACTGA	CTTCTCTCTCCACCCCATCCC	Y
NM_005640	6875	ENSG00000141384	TAF4B	18	1854	TACCTGGCCCTTCTGAAGTGA	GATCTGAAGTGACTTTCTGGAACCAAAATG	Y
NM_033400	85446	ENSG00000136367	ZFHX2	14	1287	ACGACTACCTCTACACTTCTAGCTTTATAA	CATCGGCCCTTCGAGGAC	Y
NM_021916	7621	ENSG00000187792	ZNF70	22	2395	CAGAAGATTCATTCTGGGGAGAAGCTATAG	GAGTGCTGGTTAAATGTTAAATGTTTCACA	Y
NM_005940	4320	ENSG00000275365	MMP11	22	952	GCCAACACTTTCCTCTGA	GCAGAAGGAGCCCTGAGCA	Y
NM_212535	5579	ENSG00000166501	PRKCB1	16	678	ACTAACCCAGAGTTTGTCATTAATGTGTAG	AAGTATCTTATTTTGTCATGTTCACAATGG	Y
NM_203282	9534	ENSG00000213096	ZNF254	19	2052	ACTCATTGGAGAGAAATCTTACAAGTATGA	AACTTATACTCACACAAACATACTTACTCC	
NM_006177	4901	ENSG00000129535	NRL	14	1309	TCCCACCTCTTCCTCTGA	GCCTGCCCCTCCACCCCA	Y
NM_005391	5165	ENSG00000067992	PDK3	X	533	GATGCTTCAAAATACAAAGCAAAACAGTAA	CCAGAAGTTTTTCATCTTTCCACCTGAAAC	Y
NM_006084	10379	ENSG00000213928	ISGF3G	14	549	ATTCTGTCCCTGGTGTAG	CCAGGCAGCATCTTAGCCAGAA	Y
NM_016614	51567	ENSG00000111802	TTRAP	6	991	CTTCTGTGCAACTTAGATATAATATTGTAA	TATATTCTTGGTAATTCTTGGTGGAGTGAG	Y
NM_021180	57822	ENSG00000158055	GRHL3	1	1010	AAAATTCAGATCATCCTTAAGGAGCTGTAA	GCCCTGCACGTCCCAGCA	Y
NM_006156	4738	ENSG00000129559	NEDD8	14	456	GGTGGTCTTAGGCAGTGA	GAACTGGTTCCAACACACCACTAAGTA	Y
NM_015895	51053	ENSG00000112312	GMNN	6	478	ACGGATGCAAAGCCATGTATATGA	AATGAGCTTGAGGTACAACAAAGTTTGG	Y
NM_001198965	4776	ENSG00000100968	NFATC4	14	2079	GAAGAGCCTCCTGCCTGA	TCTCTCTCCCTCTCTCTCTGTCTCT	Y
NM_004175	6634	ENSG00000100028	SNRPD3	22	744	CGTGGAAACATCTTTCAAAAGCGAAGATAA	CCTCCTGGGTTCAAGCGAATCTT	Y
NM_003743	8648	ENSG00000084676	NCOA1	2	2490	CTTCAGCAGCTACTGACTGAATAA	ATCTTCTGAGTCAAGAAACAGCAGG	Y
NM_005839	10250	ENSG00000133226	SRRM1	1	1175	GTGTCCCCACAGTCTTAG	TTCTAAATGTTCCAATCACATGCTGATTCC	Y
NM_139058	170302	ENSG00000004848	ARX	X	1154	GGCAAGGAGGTGTGCTAA	GATTCAAACCAGGGGCCTCTG	Y
NM_001256272	30813	ENSG00000100987	VSX1	20	1216	CTGATGACCTTCAGCTGA	TACTCAAAATCACTAAGGGGGTAGATTTTC	Y
NM_130839	7337	LRG_15	UBE3A	15	2068	GCCAAAGGATTTGGCATGCTGTAA	CTGTTAAAAGTCTACTTGATGTGAACAACT	Y
NM_000965	5915	ENSG00000077092	RARB	3	1495	CAGTCACCACTCGTGCAATAA	ATAGACTGCCGTGCATTAGCACA	Y
NM_015655	26152	ENSG00000130684	ZNF337	20	997	GTGGGTGAGGCTTCATCTTGA	TCAGCTGTGTTGGAGGGAGTTC	Y
NM_014238	8844	ENSG00000141068	KSR1	17	1937	CTTTCTTCTCTTTCCCTCTGTAGGTTGTAG	CAGACCTTCTGGGCTCCC	
NM_030762	79365	ENSG00000123095	BHLHB3	12	2220	GGAAAGGAAGCTCCCTGA	CCTTTGTGATCATCTATTTATACAAGACCC	Y
NM_017433	53904	ENSG00000095777	MYO3A	10	753	CTCGTCCAGCAGTCCTAA	ATTGTGTTTGTCTGAAGTGGCTGG	Y
NM_016231	51701	ENSG00000087095	NLK	17	1922	CCTCTGGTGTGGGAGTGA	GAGAAGATTTTTAAAAACAACCAATTTCCT	
NM_013375	29777	ENSG00000146109	ABT1	6	1584	CTTGTCAGGGACTCCTGA	TTTAAGACATCAATGATCTGAGGAAACAAT	
NM_005977	6049	ENSG00000127870	RNF6	13	1236	TTAGGGTCTAACATAGCAAACAATGGGTAA	CTAAGAGCAAATATGTCTTTTCCATGAAGC	Y
NM_005517	3151	ENSG00000198830	HMGN2	1	1674	GCTGGAGATGCCAAGTGA	ACCTTCAACAAGTTTCATCTGTGCCT	Y
NM_003593	8456	LRG_61	FOXN1	17	901	CCCGTGGCCCTGGCATGA	TTTCTGGAGGAGGAGTGAGATTGTG	Y
NM_002953	6195	ENSG00000281877	RPS6KA1	1	1008	CCATCCACCACCCTGTGA	CAGGCCAGCGGGATTCCC	Y
NM_002515	4857	ENSG00000139910	NOVA1	14	2256	AATCCTCAGAAAGTGGGTTGA	AACTAAGTGGGGCCCCAATCTTT	Y
NM_001260	1024	ENSG00000132964	CDK8	13	531	TCACATCAGACACATCGGTACTGA	TAAGACACTAAAAAGAAAGGTGTACCAAAA	Y
NM_001042426	1058	ENSG00000115163	CENPA	2	1488	GAGGAGGGACTCGGCTGA	ACACCCAAGAATGATCAATAAAAAAAAAGA	Y

NM_003170	6830	ENSG00000109111	SUPT6H	17	786	CTGGACGAGATGGATCGGTAG	GTCTGATGTAGAAGCTGCCTCTAGTCTATG	Y
NM_139135	8289	ENSG00000117713	ARID1A	1	1534	GATGTACTGTTTTTGATTGGCCAGTCATGA	TTTTGGAATATAGTAAATAAATGACAGGGT	Y
NM_005522	3198	ENSG00000105991	HOXA1	7	1625	ACTCTGACTACCTCCCACTGA	TATATCAGAACTCTGCCTGACAATCCAAAA	Y
NM_006735	3199	ENSG00000105996	HOXA2	7	552	ACAATCGACTTGCAGCATCTGAATTACTAA	TCCATTGAAGACTTTGAACATGCC	Y
NM_030661	3200	ENSG00000105997	HOXA3	7	1905	AAGCTCACCCACCTGTGA	AATTTGATTCCTTTCTCGAGGAATCCTTAA	Y
NM_002141	3201	ENSG00000197576	HOXA4	7	898	CCCGTTCCCTCCTCCATATAA	CATACATACACACACTCTCACACACAAATT	Y
NM_004264	9412	ENSG00000152944	SURB7	12	1468	AGCCAGTCTCTTCCAGACTCATAG	TAGTTTTCCCTCATTTGAAAAATGTCTTGA	Y
NM_019102	3202	ENSG00000106004	HOXA5	7	963	GGGGCCTTCCGTCCCTGA	AGGTAGGGAAGCGATGGGACA	Y
NM_006896	3204	ENSG00000122592	HOXA7	7	1370	GAAGAGGAAGACGAGGAGGAATGA	TAAAAAGATACATGACACCAATACATGGGT	Y
NM_152739	3205	ENSG00000078399	HOXA9	7	1345	GACCGAGCAAAAGACGAGTGA	GCGGACTGGTTGTGGCAG	Y
NM_018951	3206	ENSG00000253293	HOXA10	7	1488	GAGCTCACAGCCAACTTTAATTTTTCCTGA	CCAGGGCTATAGGGCCCAG	Y
NM_005523	3207	ENSG00000005073	HOXA11	7	1819	CAGTACTACTCAGCAAATCCACTCCTCTAA	CCAATTCCTGACTCAGTCACCTTCTC	
NM_000459	7010	ENSG00000120156	TEK	9	1122	GCTGAAGAAGCGGCCTAG	AGGAGACCTTTTCAATCTGATGAGCTTC	Y
NM_000522	3209	ENSG00000106031	HOXA13	7	1498	GTCATCAACAAACTGAAAACCACTAGTTAA	CTCCCTTTTCTTAAAAATTTGGTAGCAAAA	
NM_021969	8431	ENSG00000131910	NR0B2	1	541	ATGCTTTTGCTCAGGTGA	GAGGTGATGGGATTGTGAACTGATACT	Y
NM_199347	152110	ENSG00000163491	NEK10	3	406	CCTGCTCTGGCCACATAA	CCTGTTCAGTGTGTTATAAGAATTGCCAAA	Y
NM_013388	10113	ENSG00000138073	PREB	2	837	AGTGCCTTTCCAGGTTTCCTTTAG	CTTCCTGGAAGGGCTGCTC	Y
NM_007149	7738	ENSG00000096654	ZNF184	6	741	AGACTGCATCCTGGCATATGA	TAAGATACCTAGTCTATTGTAATTTAACAT	Y
NM_001172304	84930	ENSG00000120539	MASTL	10	563	CACCTGACTGTATCTGGATTTAGTCTGTAG	TCGGCCTCCCACAGTGCT	Y
NM_001201459	130557	ENSG00000163795	ZNF513	2	494	CACACAGACTCATCCTGA	TTGGTCTGGCCCAGTTCC	Y
NM_018492	55872	ENSG00000168078	PBK	8	864	ATTGTTGAAGCTCTGGAAACAGATGTCTAG	TTCCCAGGGGACTGTGGCTT	Y
NM_005442	8320	ENSG00000163508	EOMES	3	855	GGGTATTATGCTTTTTACACAACTCCCTAA	TTGCAAATTCTTTACAAAGCCAAAGCAC	Y
NM_001271286	84450	ENSG00000243943	ZNF512	2	1934	AAGACTAATCATAAACGAGGAAGGAAATAG	TGAGTCCTGCTGATCCCTGGT	Y
NM_175061	221895	ENSG00000153814	JAZF1	7	2406	GCTGAGATTATCAGGAAGATGCAGCAATAA	TAATCTAATCTAAGCAAAAATGTAAGGGAA	Y
NM_007266	11321	ENSG00000198522	XAB1	2	826	GCACAATACTGGAAGAGAAACAATAAATAG	GCACGTGGGAAACTTTAGTAAAATATCACC	Y
NM_001042747	2268	ENSG00000000938	FGR	1	804	CCCGGGGATCAGACATAG	CTCTGGGTGTTCTAAAACTCCACAC	Y
NM_002820	5744	ENSG00000087494	PTHLH	12	1203	GAGCTCGATTCACGGTAA	TTCTATTTTATAAAATGGGATTAATATTTG	
NM_001199480	7746	ENSG00000137185	ZNF193	6	488	GTGGCTGAGCTGGTCTAG	GAGAGTCACGTAGGTGTCAGAGAGAA	Y
NM_019110	387032	ENSG00000187626	ZNF307	6	584	CATGTAGGGAAAAAAACTCTTTCACAGTGA	GAGAATAATACATAAATTCAAATGGCATAA	Y
NM_001243241	64288	ENSG00000235109	ZNF323	6	1608	CAGAAAATCCACACTGGAGAGAGACCATAA	TCAGCTAATTGCTAATGTTCTATTGACAAA	Y
NM_001163391	NULL	NULL	ZSCAN12	6	1684	CACAAAGGAGAAAAATCTGTTTCTGTGTGA	TGGAGTTTGCTGTGGCAGG	Y
NM_001012455	222696	ENSG00000187987	ZSCAN23	6	1990	CCAGTGGCTGAATCAAGCTAG	CCACCCATCACTTCTCCATTTCTCTGT	Y
NM_001265	1045	ENSG00000165556	CDX2	13	1227	CCCACCGTCACCCAGTGA	GAGCCACGCATTCCAAGGC	Y
NM_005253	2355	ENSG00000075426	FOSL2	2	2381	ACTCTGCTGGCTCTGTAA	CAACACAGCTTGAAATCTGAACTGAAG	Y
NM_017846	54952	ENSG00000180098	TRSPAP1	1	1089	TCAGAGATCCCTGCCATGATGTAG	ACACTTCCTTTGCAGGACAATCTCTAT	Y
NM_024567	79618	ENSG00000147421	HMBOX1	8	1750	GCCCTGGATGATGACTGA	AAAAAGAAGCCGTGCTTAACATCCA	Y
NM_001135218	6883	ENSG00000120656	TAF12	1	622	GCATTGATCCGGAAAACAACCAAGAAATAA	TTATGGCCTCCCTTGGGGTAAAAT	Y
NM_024482	10691	ENSG00000162419	GMEB1	1	1010	AATGTGGAGATTGTGGTCTTAGAGGATTAA	TGTACCTTCCACTTATTACCACCTTCTTCT	Y
NM_001172828	51441	ENSG00000198492	YTHDF2	1	1003	GAACGTCAAGGTCGTGGGAAATAA	GTTAACCAGCCCTGGGTACAAAG	Y
NM_005080	7494	ENSG00000100219	XBP1	22	1166	AGCTGGAAGCCATTAATGAACTAA	AAAGCAGGCAGTAATTAAGGTGGAAAA	Y
NM_005249	2290	ENSG00000176165	FOXG1B	14	1708	GGGTCTTCTTCCAACCCTTTAATACATTAA	GGGAATCAAATAAATACCTCTACATTGTAA	
NM_004304	238	LRG_488	ALK	2	630	AACCAGCCTGGGCCCTGA	ACACTTCTGCTATACTACTGGAACTACTCA	
NM_005243	2130	ENSG00000182944	EWSR1	22	544	AGAGATCGGCCCTACTAG	GCCAGAGTCTGTTTCCATGTCACAA	Y
NM_001042539	4150	ENSG00000103495	MAZ	16	1621	CCACAGCCCACCTGCTGA	TGTATCTGTGCTGCCGCCA	
NM_002383	4150	ENSG00000103495	MAZ	16	1132	CCCTCCCAACCCTGGTGA	TGTATCTGTGCTGCCGCCA	Y
NM_004783	9344	ENSG00000149930	TAOK2	16	873	CTCAATGGTTCTTCCCACTTCTATTCCTGA	GAGATCTGGTCACACCTAAAGTCATTAAAA	
NM_002742	5587	ENSG00000184304	PRKD1	14	937	CGTGTCAGCATCCTCTGA	ACATACTTTTACTGCTTCAGAGTAGATAAA	Y
NM_004608	6911	ENSG00000149922	TBX6	16	611	TCCAAACCCATGTACTGA	ATTTTCCTGAAGAGGAGATGGGCCTA	Y
NM_019103	55954	ENSG00000100319	ZMAT5	22	449	AGAGTCCAGTGGGGCTGA	CGTGCTGAACCTCTGCTGGT	Y
NM_181353	3397	ENSG00000125968	ID1	20	857	AGATCCAGATCCGACCACTAG	TTAAAGACACCGGAAACACTCATTCAG	Y
NM_001191	598	ENSG00000171552	BCL2L1	20	1671	TCACTCTTCAGTCGGAAATGA	TCACCGTACAGGTGGATAAATTCAG	Y
NM_021253	NULL	NULL	TRIM39	6	1650	CCAACAGATTGGGAGTGA	CTCTGTCTCATAAATAAAATGTAGATGTCC	Y
NM_015355	23512	ENSG00000178691	SUZ12	17	2215	TCAAAACAGAGCAAAAAACAAAAACTCTGA	CTGCTGAAAGCAGGATTTCACATTAAGATT	
NM_152652	197407	ENSG00000180035	ZNF553	16	1181	GCAACAGGACTGGAATGA	GCAGCGCCGCGCGGTTTT	Y
NM_033118	85366	LRG_392	MYLK2	20	1080	ATGGCTCTGGGGGTCTGA	CACATCATGTGCTGGGCAGACT	Y
NM_016643	51333	ENSG00000179965	ZNF771	16	408	GAGTGTGAGGGCAGCTGA	TCACCTCCAACCAACACAAACC	Y
NM_004118	2307	ENSG00000179772	FKHL18	20	430	CCAGGAATGTTCTTCTTTGAGTAA	ACTCCCTGAGAAAGTCCCCAG	Y
NM_005516	3133	ENSG00000233904	HLA-E	6	1804	TCTGAGTCTCACAGCTTGTAA	GGGATTCACCAGGAAAACGGGAAA	Y
NM_001033566	55288	ENSG00000126858	RHOT1	17	1174	ATGTACAAAGCATTATTGAAACAGCGATGA	CCAAAGTGCTGGGATTACACGATTGA	Y
NM_033410	92595	ENSG00000169951	ZNF764	16	1610	ATATTCCAGGAGTGTGGGTGA	TGGTTTGGGATGAAAGGCAGTC	Y
NM_152458	146540	ENSG00000197162	ZNF785	16	2067	TTTCCAGATATATTTCAAGAGTGTGGGTGA	TTTTTTTGTATTTTTAGTACAGACGGGGTT	

NM_138447	115509	ENSG00000156853	ZNF689	16	1065	AGGGGCAACGCCCATTAG	TTTGCACTTCCTGCTGCTAAACC	Y
NM_001270710	NULL	NULL	NRM	6	879	CCTGGCTCACGGGCTTGA	GGGGGCGGGTCTGCGGGG	Y
NM_002110	3055	ENSG00000101336	HCK	20	515	TACCAACAGCAGCCATGA	TATTATAGGTGGGAGCCACAGCAC	Y
NM_005204	1326	ENSG00000107968	MAP3K8	10	1177	CCAACGCTTGAATATGGCTGA	CAAGAGCAATAGAAAAGGAGTGTATTAATA	Y
NM_006662	NULL	NULL	SRCAP	16	576	CGCAAGGCCAAGACGTGA	CTTGGGTAGATTTCTGCCTGGAACATATAG	Y
NM_001172432	5261	ENSG00000156873	PHKG2	16	1196	GGAAAGCTGATGGCTTGTGTATGA	TGAGATGGCAAGTTACAGCGG	Y
NM_001142544	1489	LRG_408	CTF1	16	1201	CCCGGGGGCTCGGCCTGA	GGGCTGGCAGAGGGCACC	
NM_014712	9739	ENSG00000099381	SETD1A	16	817	CGGGGCTCCCTAAACTGA	TCTCTGCAGGTCTGGGCTCT	Y
NM_015338	171023	ENSG00000171456	ASXL1	20	2161	GTATTGTGCCTTGTGGTGAGATAA	TAACCTCCCAAAAGAAGAGCTCCAAGTATA	Y
NM_014717	9745	ENSG00000198597	ZNF536	19	1074	AAATCTGCACATTTTCTTGCAGGTAAGTGA	AAAATGCAATGATCTGCCCCG	Y
NM_014699	9726	ENSG00000167395	ZNF646	16	600	CTCAGCTTCTCCCTCTGA	GGAAGGAACCTGCCTGGG	Y
NM_001122957	10295	ENSG00000103507	BCKDK	16	940	GGACCCATGCACGGGTGA	GGCTTGGGAAGCAGTTGGC	Y
NM_005881	10295	ENSG00000103507	BCKDK	16	706	GAAAGCTTCCGGATCTGA	GGCTTGGGAAGCAGTTGGC	Y
NM_007109	6941	ENSG00000224379	TCF19	6	1678	GCTGGCATTCAGACCTAA	GCCTGGGACACAGTAGATAGACAC	Y
NM_002701	5460	ENSG00000233911	POU5F1	6	444	TCTCCCATGCATTCAAACTGA	CCTGTACAAATAAACTTGGCTGCAATC	Y
NM_175848	1789	LRG_56	DNMT3B	20	1633	CCTCTGAAGGACTACTTTGCATGTGAATAG	TAAGCTCCAGTCAGCTTGGGG	Y
NM_014676	9698	ENSG00000134644	PUM1	1	1885	CCCCCTAATGGTATCATCTGA	ATTGGGCTCAAAGAGCCATTAGTTTG	
NM_005931	4277	ENSG00000234218	MICB	6	1395	TCCACTGAGGGCACCTAG	GTCAGTCATTGGGAACTAGCAGGG	Y
NM_015927	7041	ENSG00000140682	TGFB1I1	16	564	CTGAAGCTCTTCGGCTGA	GGAAGTGCAGGAGTGGCTAC	Y
NM_016733	3985	ENSG00000182541	LIMK2	22	1819	CGGGACTCACCTCCCTAG	TCTAGAGCAGATGGGTGTCTGCTT	
NM_014323	23598	ENSG00000100105	PATZ1	22	1267	GGGCCTGAAGGGAAATGA	ATCTACCTGTCAATCCCAGTGGAACT	Y
NM_032052	23598	ENSG00000100105	PATZ1	22	1652	ATGCTCACATCAGGATCCGATTGA	ATCTACCTGTCAATCCCAGTGGAACT	Y
NM_020856	57616	ENSG00000121297	TSHZ3	19	1782	CTGTATGTCTCTGAGTTAGAGAAGCAGTAG	AAACTCCCCCTGATTTACGCACA	Y
NM_016505	51538	ENSG00000121766	ZCCHC17	1	920	AAGAAGAAGCACAAGGAGTGA	AGTGCCTTTGGCTCTGTACCTC	Y
NM_025256	10919	ENSG00000227333	EHMT2	6	504	CCCCCTGTCAACACATGA	CATAGTGGCCCCCCACCT	Y
NM_181842	221527	ENSG00000234196	ZBTB12	6	489	ATTAACGTCCTCATCCGCTAG	ACTCCCTCACCCCAGGTCTATTCT	
NM_001265588	NULL	NULL	ZNF267	16	1007	CAGAGAAGTCATACTAGAGAAAAACTTTAA	CTGGTCTACAGCCTGGGAATG	
NM_032454	8859	ENSG00000234947	STK19	6	577	CGCCTGCCAGAGACATGA	CTCACCACTCCACAGGGAGAAT	Y
NM_001137674	344787	ENSG00000197385	ZNF860	3	938	AAGCGTGGCAAGGTCTTCAGTTAG	GCACCTGGCATTTTTTTTTTTTTAGCCT	Y
NM_004381	1388	ENSG00000168468	ATF6B	6	651	CTCTACCTCAATCATCCCTGA	GGGCAAGGGGATGACATGC	Y
NM_002586	5089	ENSG00000206315	PBX2	6	1829	GTTCACTCTGATACCTCCAACTGA	AGGCCAGACTGTTGTCTGCAA	Y
NM_005225	1869	ENSG00000101412	E2F1	20	1426	ACCCCCCTGGATTTCTGA	AGCCCACTGTATTTGTTACATGTTTACTAA	Y
NM_032819	84905	ENSG00000131061	ZNF341	20	932	ATCCAGGCCTCCGAGTGA	CCGTGCCCAGCCTTGCTT	Y
NM_024424	7490	LRG_525	WT1	11	1462	CTCCAGCTGGCGCTTTGA	TGAAGTTTGACAGAGAGAAATAAATTGTGG	
NM_176812	128866	ENSG00000101421	CHMP4B	20	987	AACTGGGCTGGATCCATGTAA	ACTTCTGGTGAGACAAGCTCTGC	Y
NM_002197	48	ENSG00000122729	ACO1	9	903	ATCCGCAAGATGGCCAAGTAG	CGTTATACCCGTACAGTTCTGATAGAGTTG	Y
NM_003253	7074	ENSG00000156299	TIAM1	21	2130	AGGAAACTGAACACTGAGATCTGA	TTGTTAAAACTTCAAGCGCTACAACAG	Y
NM_006559	10657	ENSG00000121774	KHDRBS1	1	1427	TACAGAGAGCACCCATATGGACGTTATTAA	ACTTAAAATAAAATCCCCCCCACCACTTTT	Y
NM_001130710	23658	ENSG00000106355	LSM5	7	2108	GGAGAAGGACCTGAAGTGTGA	ATTAGCCAAGGCAGTAGTACTGACAACA	Y
NM_001714	636	ENSG00000151746	BICD1	12	760	AAGCCTCCTCACCCCTAG	ATGTCCAGTGAAGAACTCTAGTTAAAAATA	Y
NM_005802	10210	ENSG00000197579	TOPORS	9	1023	TGTCTTGGTAGAGACTGTGATATGTCTTAA	AGGCAAAGGCAAACTGAAGGC	Y
NM_013960	3084	ENSG00000157168	NRG1	8	1882	CCCCATTTGGGCTTCATTCTCTAA	GTAAGGTGTTAGATTTACCCTGATAGCAAG	Y
NM_153809	NULL	NULL	TAF1L	9	825	AAGGATGGGCACGGAAAATGA	TACCTACTATGCACAGAACCGTATCCTAAG	Y
NM_005356	3932	ENSG00000182866	LCK	1	629	TACCAGCCTCAGCCTTGA	GGAGGGGTGAGGAGCACTG	Y
NM_004964	3065	ENSG00000116478	HDAC1	1	759	GAGGTCAAGTTGGCCTGA	AAACCTCAAGGGAGGAGTTAAGGC	Y
NM_001135178	84307	ENSG00000186812	ZNF397	18	1059	CGCCATCAAAGAGTCCACACTATAAAGTAA	AGTTCTAGGATAGCCTACAGGTTGG	Y
NM_001039111	131405	ENSG00000206557	TRIM71	3	648	AACAATCGAATCCTCGTCTTCTAA	AGGAAATTTCTCTGTTGCAGCATTTAAAAA	Y
NM_145756	252884	ENSG00000186496	ZNF396	18	1834	TCACTTTCAGGAGGCAGATAA	ACTGTTGTAAGGACAGTGATGAACATTTGA	Y
NM_001199456	6046	ENSG00000235307	BRD2	6	967	ACCAGTGATTCAGACTCAGGCTAA	CAGGAGAAAATTCACACATCCAGTGC	Y
NM_000059	675	LRG_293	BRCA2	13	1082	GACACAATTACAACTAAAAAATATATCTAA	ACAAAGCAAGACTCCATCTCCAAAAAAAAA	
NM_175069	54840	ENSG00000137074	APTX	9	1258	TTGGTATCTCATATTGGTTTTCCAGAGTAG	TCTCTCCAGAAGAAGCTTTTGTTAAGGG	Y
NM_001270401	6257	ENSG00000231321	RXRB	6	1277	CCCCATCAACTGGCCTGA	CGACAAAGCTTGCCTTGTGTC	Y
NM_002931	6015	ENSG00000235107	RING1	6	503	ACCAAGGATCCAAAGTGA	GCTAATTTGGAGTTTCCGACGACATATCTC	Y
NM_014071	23054	ENSG00000198646	NCOA6	20	728	TCCAAGCGAAGAAAATCCAAGTAA	AAGCCAAGAATGAGGTGAGGGA	Y
NM_002504	4799	ENSG00000086102	NFX1	9	1359	ATAATTGACTATTTTGACGTCCAGGACTAA	CCCTCCAGTCACCTGTTCCT	Y
NM_001048200	10114	ENSG00000110422	HIPK3	11	1005	ACAAAACTCAGCCAGTATCCATATATGTGA	ATCTACCCAAAGTCATGCCTACTGTAC	Y
NM_002636	5252	ENSG00000112511	PHF1	6	759	GTGCCCCCCACTCGATGA	GCCTTCTGACTTCCAGGTGATGA	Y
NM_152735	221504	ENSG00000236515	ZBTB9	6	1201	GCCCACTGGACTTACAAGTGA	TTCTTGGCACCATCCAAGGAATG	Y
NM_001128160	7342	ENSG00000153560	UBP1	3	2174	AGTGATGGCATCCACATAATTTTGAAGTGA	CAGCTTTCAGAAAATAAACTGAAAAGGAAA	Y
NM_004795	9365	ENSG00000133116	KL	13	2139	TCGAAGAAAGGCAGAAGAAGTTACAAATAG	TGGGTCGCCCTCCCACCC	Y
NM_152493	149076	ENSG00000160094	FLJ25476	1	1853	GTGCGAATCTCTCTCATCTGA	CTCAGTTCAGCACCTGGAGTGA	Y

NM_198040	1912	ENSG00000134686	PHC2	1	1422	AGCATGCTCAAGGACTCCTAG	ACCCTCAAATGACCCAGCTC	Y
NM_145238	7579	ENSG00000121903	ZNF31	1	1099	GGAGGGAAGGCGTCGTAG	AGCAGTATCTTATTGGAAGAAAAATTACTT	Y
NM_003895	8867	ENSG00000159082	SYNJ1	21	2416	TCACCCACACTGGACTTTACAGAAAGATAA	TCTGGAGAAATGAAAATAAGGTCTTGAATT	Y
NM_016631	94104	ENSG00000263141	C21orf66	21	1231	GAATTTAAGTCTTTGATCGAAGGAAAATAG	AAGATAAAATTAACAGAGCAAATACAGTGC	
NM_003487	8148	ENSG00000270647	TAF15	17	477	CGCAACCGACCATACTGA	CTGGGAACCACTAAACACAGTAGGA	Y
NM_145901	3159	ENSG00000137309	HMGA1	6	1527	TCGGAGGAGGAGCAGTGA	CATCAAGAGGCTTAAAATGACCTTCCC	Y
NM_001163120	201299	ENSG00000278023	RDM1	17	441	AGGCTGCCAGAACTTGACTAG	GTCACGTGAGGAGGGGCCAT	Y
NM_001242600	9584	ENSG00000131051	RBM39	20	1052	CTACTGGTTCCAAGTAGACGATGA	TAAAGGAATTCATGGGGGACAACGT	Y
NM_005806	10215	ENSG00000205927	OLIG2	21	1541	ACCTCCGACGCCAAGTGA	AGACCCGGAGGATTTGCC	
NM_002915	5983	ENSG00000133119	RFC3	13	1392	GGATTGGAAGGCATGATGTTCTGA	TCTCTTATATTGCTGGTAGAAGTGTAAAGT	Y
NM_138983	116448	ENSG00000184221	OLIG1	21	1540	GCGCAATTCTCCAAGTGA	CTACAGTTGCCTGTTCATTGGATTATTTTA	
NM_001422	2001	ENSG00000135374	ELF5	11	1603	CAGGAAGACAAGCTATGA	CTTGGGCAACAAGCAAGAACC	Y
NM_001252294	25803	ENSG00000124664	SPDEF	6	652	TTCGTGCACCCCATCTGA	CAATCAATTATCCCTTGCCCTTTAGGCT	Y
NM_003093	6631	ENSG00000124562	SNRPC	6	467	ATGACTCGACCAGACAGATAA	AACAAAAGCTCTGCAAGTGCTAGATTTAGT	Y
NM_024835	79893	ENSG00000278311	ZNF403	17	615	ACAACGGCTGGAGCAAATTAA	CAATTCTCATCAAAGGAAGATTTTTGTCTT	
NM_138927	6651	ENSG00000159140	SON	21	1262	AATTGTATGTTTTTCTTGAATAGGTATTGA	GTGTGTGTGTGTGTGTGTGTGTG	
NM_017613	29980	ENSG00000159147	DONSON	21	934	AGAGACTACATTTATAATTGGAGATCCTGA	TACAGTGGTATTCATATGCTATGTCTCTAA	
NM_021249	58533	ENSG00000129515	SNX6	14	1890	GCAGTGTTAAATGGAGACACATAA	AGGGGCCAAGGGATGCAC	Y
NM_018443	55900	ENSG00000089335	ZNF302	19	1340	CATACTGAAGAAAAACCGTTTGAAGTTTAG	TATGGACTCAATAATCCTCACCAAAATACT	Y
NM_013448	11177	ENSG00000198604	BAZ1A	14	957	GCGAAAAAGTCACGAATCTGA	TGAAATGGATCTTACTCCTCAAGGGTTT	
NM_001029997	339318	ENSG00000197841	ZNF181	19	952	GAGAAATCTTACAGAAGAGAAACTGTATGA	ACTCTGAATAATCCTCATCAAAATACTGCT	
NM_001007248	148103	ENSG00000153896	ZNF599	19	1140	CACCATCGAAAGATTCATACCAGAGTTTAA	TATAGTATAGGTGTTCACAGTACCATTTAC	Y
NM_003427	7629	ENSG00000065029	ZNF76	6	915	TCGGAGAGTGGCTGCTGA	TTCCTTCCCACCTGTGTCCC	
NM_005568	3975	ENSG00000273706	LHX1	17	1667	GAGGCGGCCGTGTGGTAG	TGGCTGGCATTAGCAAAAGGTTTC	
NM_006238	5467	ENSG00000112033	PPARD	6	2292	CTCCAGGAGATCTACAAGGACATGTACTAA	GTCCACCTGACTGACAAGCG	
NM_194325	90075	ENSG00000168661	ZNF30	19	514	CTGAGAAAACATATGAGTGTTATACCCTAA	CTGTAAGGTGATAAGTGTAGTGCCTAATAT	Y
NM_003214	7005	ENSG00000007866	TEAD3	6	1627	GTCTACAAGCTCGTCAAAGACTAG	GGAGGGAGTGAGGGAAATGTTGA	Y
NM_003322	7287	ENSG00000112041	TULP1	6	633	AAGCTGGCCTGCGAGTGA	TTTCGGTAGGACCCGCGA	Y
NM_001008494	91464	ENSG00000175329	ISX	22	1874	AGCATCTGTGCTACTTCAACATAG	CCTCAGCCTAGCAAAGACCC	Y
NM_183013	1390	ENSG00000095794	CREM	10	1740	AAAGATCTTTATTGCCATAAAGTAGAGTAA	TCTCCCACCACACACTGATCAGAT	Y
NM_006285	7016	ENSG00000107140	TESK1	9	479	CCTGGGGCACGCTCTTAG	CTGACACTTCCAGCCAGTCTG	Y
NM_002895	5933	ENSG00000080839	RBL1	20	1164	GATGTTGTCAGTGAAAGAGCAAATCATTAA	CATAATCATGGTGTTACCTTACTAATAGGA	
NM_005066	6421	ENSG00000116560	SFPQ	1	1036	CCAAACAAAAAACCCCGATTTTAG	TCTCATGATTTGCCCACCTCAG	Y
NM_001003681	10042	ENSG00000100281	HMG2L1	22	2336	ATCATGCCGGGACTGTGA	AAGAAAATGGCTAAGATTAGAAATTTAATC	
NM_006368	10488	ENSG00000107175	CREB3	9	462	TTGCAGGACAGATACTCAGGCTAG	CTGTGGGAGGGAAGTGCTAACA	Y
NM_003367	7392	ENSG00000105698	USF2	19	739	GAGGGCACCCGGCAGTGA	CCATCCAACCAACCACTGGGTAAT	Y
NM_003137	6732	ENSG00000096063	SRPK1	6	2450	CCTTGGCTTAACTCCTAA	AAACTATGAACCCCACAAAATGGCAAG	Y
NM_080600	4099	ENSG00000105695	MAG	19	1673	GTTTCTACCCTGGAATCTCACTGA	GGCCCTGGGTTCAAGGCTAAAAT	Y
NM_003995	4882	ENSG00000159899	NPR2	9	466	GGACCTCCTGGACTCCTGTAA	GAGGTCGGGAGGAAGTTAATTCTCAC	Y
NM_006739	4174	ENSG00000100297	MCM5	22	427	GTTCTCTACCGCCTCAAGTGA	ACTCAAGTCACGTGACTTACCCAGAA	Y
NM_020529	4792	LRG_89	NFKBIA	14	683	CAGCGTCTGACGTTATGA	CCTGCAAAGCTTTGCTGGAG	Y
NM_198291	6714	ENSG00000197122	SRC	20	2219	CCCGGGGAGAACCTCTAG	TGTCTCCAATCTGATGTCACCCCT	Y
NM_001165923	6928	ENSG00000275410	HNF1B	17	1121	TGTCCTCTACAAGCCTGGTGA	AGAGAAGGCCACCCACCTCT	Y
NM_005584	4081	ENSG00000180660	MAB21L1	13	1450	ACCAACCCGAAAAGTTTGGAAAAACTTTAG	ATGCACTGTATTTTATTTTAAAATAACTTA	
NM_178548	339488	ENSG00000116819	TFAP2E	1	830	AAGGATGCCAAGCATCGGAAATAA	CACACCTTTCTTCCCTCCCAAC	Y
NM_001122607	NULL	NULL	RUNX1	21	571	ACAGCACCCTGGAGATGTTAA	CACCAATACCTTTATCCACTCTGGG	Y
NM_014727	9757	ENSG00000272333	MLL4	19	501	CGTCGGTTCCTTAACTGA	CCGTAACAGACCCCATGACTGAC	Y
NM_001207035	51513	ENSG00000010030	ETV7	6	601	ATCTTCATATTCATATGGAGTTTCCAGTGA	TTCATGTCCTTTGCCCAATTTGG	Y
NM_016135	51513	ENSG00000010030	ETV7	6	631	AGGCCAGAAATCTCTCCGTGA	GGGCAGCTGTAATCCCAGCTA	Y
NM_007271	11329	ENSG00000112079	STK38	6	2072	ATACCTTCCTACATGAAAGCAGCAAAATAG	GGTTAAAAGCAGCCATCAGCACTC	Y
NM_152658	199745	ENSG00000161277	THAP8	19	635	CGGATCCCCAGTGCATAA	ACTGCAGGGGTTGTTCGTC	Y
NM_000389	1026	ENSG00000124762	CDKN1A	6	1719	ATCTTCTCCAAGAGGAAGCCCTAA	CTGTTGTTTTTTGCAGCAGTCTTTCTGTTT	Y
NM_001256693	9833	ENSG00000165304	MELK	9	526	GAAGACATCCTATCTAGCTGCAAGGTATAA	AAAATTATGCAAATGAATTCTTATCTGTTT	Y
NM_007145	7705	ENSG00000167635	ZNF146	19	1634	ATTAGACACCAGAAAATTCATACTCACTAA	TATGAGAACTAATGCCACCGCTGATC	Y
NM_017826	54937	ENSG00000120669	SOHLH2	13	1006	CAACAGTTTTGGGCGTATTAA	GTATCCTCAAAGAAAAGTTGTACAATTTGA	Y
NM_005119	9967	ENSG00000054118	THRAP3	1	1519	AATATACAGCCCACAACCGAGTAG	CCAGGAAGGACTGTGTCTGTCT	Y
NM_032017	83931	ENSG00000196182	STK40	1	2311	CGCTACCTGCGGAAATAA	GGGAGCAGGGAGGGGGCA	Y
NM_016734	5079	ENSG00000196092	PAX5	9	2206	GCCTATGACCGTCACTGA	GGTTGGGGGTCAGGAGCC	Y
NM_032881	84967	ENSG00000181817	LSM10	1	508	TTTCCCCCAAAAAACTGTAAGTGA	TGCAGCCAAGCTTGAGTCTG	Y
NM_007144	7703	ENSG00000277258	PCGF2	17	1506	GTGCCCCCCTTAACTTGA	ATTTTAACCTCTCTGTGGGCTGG	Y
NM_003317	7080	ENSG00000136352	TITF1	14	1059	TACGGTCGGACCTGGTGA	CCTGGCCTCCTTACCTCCTTAA	Y

NM_001079668	7080	ENSG00000136352	TITF1	14	1059	TACGGTCGGACCTGGTGA	CCTGGCCTCCTTACCTCCTTAA	
NM_014360	26257	ENSG00000136327	NKX2-8	14	1071	GTCTCCTGGAACTGGTGA	GGCGGCCCTCCCTCAGCT	
NM_001256838	84911	ENSG00000161298	ZNF382	19	1227	GTAGAAACCACGGGAATTCAGTAA	CCATAGGTAAAGACTATATTCCTCCCCACG	Y
NM_153257	92283	ENSG00000197808	GIOT-1	19	1452	CATCCTAGTCTAGCATCATGA	TGTGTTGCCCACGCTGGA	Y
NM_002648	5292	ENSG00000137193	PIM1	6	1517	CCGGGGCCCAGCAAATAG	TCAGGAAGATGACTATCCTAGAAATCCCAA	Y
NM_006194	5083	ENSG00000198807	PAX9	14	1534	ACGGCTTCCGCGCTCTGA	CTTCCTAAACAGAAGGGATTAAAAACTGTA	Y
NM_170676	4212	ENSG00000134138	MEIS2	15	1332	GTTATGGACATTCATGCCCAATAG	AATAGACATTGAAAGGCTATAGATGATTAC	Y
NM_152603	163081	ENSG00000189042	ZNF567	19	835	CACAAGGGAGAAAACATTGAAATGCAATAA	AGCCGCAAGGTTTCTTTTTAGTAGAAATTC	Y
NM_001242476	25850	ENSG00000251247	ZNF345	19	1458	AAACTCTGCGAATTGGAAACTATAAATTGA	AAAGGAACTGAGGCCAGGAATG	Y
NM_000538	5994	LRG_103	RFXAP	13	2030	CAGTTTCCAGGAACATCAATGTGA	GGCAAAAACAAGTCACAAGGCCA	Y
NM_017438	54093	ENSG00000185917	SETD4	21	1756	AGTTTGCAAACAGCTTTTACCTGA	GGACGCATTACATCACCCAAGCT	Y
NM_001204839	374900	ENSG00000198453	ZNF568	19	513	GGTGGCTCAGAACTTAGTTGA	GGCCTGCATTTATATTTTGAATCATCTGAT	Y
NM_144689	147923	ENSG00000197050	ZNF420	19	871	TTTAGTCATGGCTCACAAGTTTACATGTGA	AAAAGCAATACATTCAAGTTTTTAAAAAAT	
NM_199126	199704	ENSG00000196967	ZNF585A	19	1670	CAGAGCAGCCACGCTTGA	GCAATGTGGGAAATTGGGAATCAG	Y
NM_145203	122011	ENSG00000180138	CSNK1A1L	13	1163	AAAAACAAGAATAATGTGAAAGATAACTAA	TTAGTCAGTGGAAGAAGTAACAATACCAAG	Y
NM_018310	55290	ENSG00000104221	BRF2	8	790	GTCCCTAACCCTCCCTGA	AGACATATTAGAAGAAAAAAAAAAGCTTTG	Y
NM_006160	4761	ENSG00000171532	NEUROD2	17	1863	GAGGAGCTCAATGCGTTTTTTCATAACTGA	GGGTGGTTAGGGAGAGAATACTACC	
NM_181786	284459	ENSG00000181666	HKR1	19	860	CATCAGAGGACACACTCAGGATAG	CGACAGCCCTCTGCCTGG	Y
NM_004448	2064	ENSG00000141736	ERBB2	17	798	CTGGACGTGCCAGTGTGA	CCCCAACCCTGAAGCTGGA	Y
NM_152484	148266	ENSG00000196437	ZNF569	19	1619	GTTAGACACCAGAGAATTCATACTCATTAG	AATCCATTTTAAAATTATTTCTCCAGGCCC	Y
NM_025079	80149	ENSG00000163874	ZC3H12A	1	946	CAGCACCCCAGTGAGTAA	TTGTTAGAAATCTTCCTTCCAGAGAAGGGT	Y
NM_004674	9070	ENSG00000129691	ASH2L	8	1189	CCCCCATGGGAACCCTGA	TACAGGTGCCCGCCACCA	
NM_014462	27257	ENSG00000175324	LSM1	8	529	CCTCGAGCAGATACTCTTGATGAGTACTAA	TGAGATGACTGGAATAAAAATATTCCTTCC	Y
NM_016536	51276	ENSG00000180479	ZNF571	19	525	CTTACTCAACATACAAGGCTTCATAATTGA	TCTAAAATAAAGTATCTGTTGTTCTAAAGG	Y
NM_004496	3169	ENSG00000129514	FOXA1	14	1993	CCCGTCCTAAACACTTCCTAG	ATGCAAAATAGCGGCTACCCTAAAATGT	Y
NM_005069	6493	ENSG00000159263	SIM2	21	2297	ATCACCAACGGGAGGTGA	AAAGGAATTCCTTTCCCTGGGAG	Y
NM_152605	163115	ENSG00000196381	ZNF781	19	1596	CCTTCAAGCATAATGCACATCTAA	GAGAAATTGTGATGGTGGATTTCATTATAT	
NM_001267797	9862	ENSG00000008838	MED24	17	612	AAAGCCATTGCTGCACTCTGA	CCCACATAGGACCCTGAGC	Y
NM_001172677	84775	ENSG00000198182	ZNF607	19	1857	CTTGAAGTACATCAGAGAATTCATATTTGA	CGGAATGAAAGCCCTCTTATGGTTTTTACT	
NM_173641	284656	ENSG00000183317	EPHA10	1	1274	CGTGGGGTGGGAGTGTAG	CACCACTCTGTGTATCCCTCTGTTATAG	Y
NM_145011	219749	ENSG00000175395	ZNF25	10	2440	CACACAAAGAAGAGGAATGCTGAGAAGTAA	TTGTTATTGGGTTACGTTAGCTAAGTATGA	
NM_199334	7067	ENSG00000126351	THRA	17	761	GTCTTTGAGGATCAGGAAGTCTAA	TGGGGGCATTTCTCTCTGATGT	Y
NM_006941	6663	LRG_271	SOX10	22	1363	ACACTGTCCCGGCCCTAA	GTGGGGAAAGAGGGAGGGA	Y
NM_002699	5453	ENSG00000185668	POU3F1	1	1717	CCCGGCTCAGTGCAGTGA	TGCAGGGTCATCTTCCTCCAAGA	
NM_000964	5914	ENSG00000131759	RARA	17	1597	GCCACCCACTCCCCGTGA	GGCAGCTTGGAAGGGTGC	
NM_001067	7153	ENSG00000131747	TOP2A	17	1178	GAAGAGTCAGATGAAGATGATCTGTTTTAA	AGGTCTTAATGCCTATCTCTTGGACTTATC	Y
NM_001161573	23764	ENSG00000185022	MAFF	22	1812	CCGGCCTCCTGCTCCTAG	GTCTCTGTCGCCATCTCCCC	Y
NM_004647	8193	ENSG00000011332	DPF1	19	1234	GCTTACATCACCCTCACCTAG	CAAAGCCGGGGAGAGGCA	Y
NM_003079	6605	ENSG00000073584	SMARCE1	17	1241	CCCATACCAGAAGATGAGAAAAAAGAATAA	AGTTGGGTGAGACAAGGGC	Y
NM_001142650	92906	ENSG00000143889	HNRPLL	2	1176	CTTTGCTTTTCTACATCATCCCATTTATAA	AAACAAAATGGCCACATCAGTTTTTCTATT	Y
NM_000223	3859	ENSG00000263243	KRT12	17	663	CAAGTTCAGGAAATTGAAGAACTAATGTAA	CTTAATCCTCAAACCCTGACATCTTCATCC	Y
NM_198963	90957	ENSG00000163214	DHX57	2	745	ACAATTGTGAAACTTGTCACCACACAATAA	GATTCGTGTCTGGGTGAGTTGATAAGA	Y
NM_002913	5981	ENSG00000035928	RFC1	4	1492	AAAGGAAAAGGAAAAAGTTCGAAGAAATGA	AGAAGACCTAGGGAGACGTGACTT	Y
NM_005461	9935	ENSG00000204103	MAFB	20	2182	TCTCCCGAGTTCTTTCTGTGA	CAAGGAAGGGATTTGCTAGGGCTT	Y
NM_001533	3191	ENSG00000104824	HNRPL	19	514	GCTCAGCACGCCTCCTAA	GACTCAATTGCTAGCACCATTCTCAGT	Y
NM_012333	26292	ENSG00000214114	MYCBP	1	2360	GAGGAGAAGCGTGCTGAATAG	TTTAGGCCCAGCAGTTTGGGTATT	Y
NM_030772	81025	ENSG00000131233	GJA10	1	664	GTTCCCACAGATCTTCAGATCTAA	AAAACACGGGTATTTTAAATTTTAATCCTT	Y
NM_012237	22933	ENSG00000068903	SIRT2	19	780	AGGGAGAAACCCCAGTGA	GGGGTCAGAGCCACGAGG	Y
NM_001009565	344387	ENSG00000205111	CDKL4	2	799	CAGGTACTTCCGCTCAAAAGTTAA	ATTACCAACTCAGTTTATTATTAGGCAATG	Y
NM_003618	8491	ENSG00000011566	MAP4K3	2	1532	GGTCATGAAAACAGTTACTGA	GCTTCATGAAACTGAAACCCCATATCAAAT	
NM_001014835	10298	ENSG00000130669	PAK4	19	1007	CAGAACCGCACCAGATGA	TGCTATGACCTGAACTCCCCACT	Y
NM_001243432	2078	ENSG00000157554	ERG	21	554	TTTTGTATAGGTTGGACCCAATGA	CCAAAGGTATTTCAGAAGCAGAGAGAG	Y
NM_020862	57622	ENSG00000128011	LRFN1	19	996	CTGGAGAGTACCGTGTGA	ATCTTCCCATCTTCCTTCAGTCTCACTTAG	Y
NM_018028	55095	ENSG00000179134	SAMD4B	19	1550	GACAAAACCTCCACCATCTGA	ACCCCCTGGCTTCTTCCC	Y
NM_003407	7538	ENSG00000128016	ZFP36	19	893	ATCTTCAATCGCATCTCTGTTTCTGAGTGA	CTTTCAGGTGAACGGCCAGG	Y
NM_001123383	54880	LRG_627	BCOR	X	1043	GCCTCAGACAACTACTGGTGA	AGTATAACTCCTCCAAAATCCTCCAGTCTG	Y
NM_002230	3728	LRG_401	JUP	17	1317	GACCACATGCTGGCCTAG	CCCCCAACACTCCTGGCT	Y
NM_025264	80745	ENSG00000138050	THUMPD2	2	855	TATAAGAAGTCGCACTCTTCTGGACTGTAG	GGAAAACAGATTTTATGACTTCTGCTTTGG	Y
NM_001130824	6829	ENSG00000196235	SUPT5H	19	449	AAGCTCCTGGAAGCCTGA	AGATTTAGGTAGGGTCCAGTTATTTGC	Y
NM_003819	8761	ENSG00000090621	PABPC4	1	1065	GTTGCTGCTGCTACCTCTTAG	GGATCATATTTAACAGTTCTGTGTATCTTG	Y
NM_153232	163126	ENSG00000176396	EID2	19	742	GGTTGCGATAAGTTTATCAATAGAGAATAG	CTATTAGAGGTCATCGTTGGTACTGGATGG	Y

NM_032221	84181	ENSG00000124177	CHD6	20	2243	CTCAAAGACTCCAACAACGACACCAATTAG	AACATACAGATTCCATAAGGATAACAAGGG	Y
NM_003718	8621	ENSG00000065883	CDC2L5	7	2334	GGCAGAGGGTTACCATACTGA	ATGTCTCACAGTAGTGCTGTGTAAGGATAA	Y
NM_001256295	2114	ENSG00000157557	ETS2	21	2245	CCCGACACGGAGGACTGA	CTAAGTGACTTGTGTATAAACCAGTTAAAC	Y
NM_001195007	10450	ENSG00000084072	PPIE	1	1470	AAGGTTGAGAGCCACACCATCTAG	GGCTGTTTACTGGAGCATAAAGATGCT	Y
NM_004310	399	ENSG00000168421	RHOH	4	879	TTCTCCATCAATGAGTGCAAGATCTTCTAA	AAAGTGACACTCTCTTGCTTCTGTTC	Y
NM_006484	9149	ENSG00000281320	DYRK1B	19	545	ACAGCAGCCAGCTCGTGA	AGACTCATGCCTGCAACACG	Y
NM_020737	57497	ENSG00000156564	LRFN2	6	489	ATGGAGAGCACGGTCTAG	TCTTGTCAATTGGCAACAATTACCCACTAC	Y
NM_001033082	4610	ENSG00000116990	MYCL1	1	2128	AAAAGAATTGCATACCTCACTGGCTACTAA	ATGTCTGAAATGGCATTTCAGGAACC	Y
NM_003152	6776	ENSG00000126561	STAT5A	17	1453	AGAGGCTCCCTCTCATGA	AAACCACAGCAAAGCGGG	Y
NM_004229	9282	ENSG00000180182	CRSP2	X	2443	CCTGGTGGGCGTCCATAG	AAGTACTTTTATACATTCTGAAATGGCATT	Y
NM_001211	701	LRG_489	BUB1B	15	557	GCTTTGCTCTTTCAGTGA	CCACCTAAAGAAATAGTTGGCTACTCTGTC	Y
NM_178544	339327	ENSG00000281526	ZNF546	19	2006	ATTAGTGAGGAAGTCCTATGCATAATGTAA	GGTGTGAAGTTACTGGGGGTCAAAT	Y
NM_001145093	285267	ENSG00000177873	ZNF619	3	2176	AATCCTTTGTCTCACTCCCTGTAA	TTCCAGATTTTTCAAGCCTCTACCCATTAC	Y
NM_012232	284119	ENSG00000177469	PTRF	17	2418	AAGAGCGACAGCGACTGA	GCAATTATTGTTATCTCCCTGGCCTTC	Y
NM_001256168	253639	ENSG00000177842	ZNF620	3	1538	CAGAAGACACCTGTCCAAGCATAG	AAACCTATGAGGTAGATTAACTGTCATCCT	Y
NM_001128629	56924	ENSG00000259288	PAK6	15	1573	CAGACCTCCACCTGCTGA	CCACAGGCCACCAGGGAA	Y
NM_001142577	284323	ENSG00000280568	ZNF780A	19	1704	ACAGGTGAGAAGGCATCTTGA	ATTTGAGCGCCTACATTCATAGGATTTC	Y
NM_012421	6018	ENSG00000117000	RLF	1	654	ACAGATGAGCTTTGTGTAGGAAGTTCATAA	AAAAAAAAAAAAGAACCTTGTTCTGAATG	
NM_170607	6945	ENSG00000108788	MLX	17	1780	CAATTGAAAAACCAGCTTTACTGA	GAGGCAGAAGTACTGTAAGGAGTGGA	Y
NM_014952	22893	ENSG00000140320	BAHD1	15	2292	ATCCTTAAGAACCCCCAGTAG	TTCCCTCACTCTCTTTTCCCTCTG	Y
NM_198578	120892	ENSG00000188906	LRRK2	12	1699	GAAAAAATGAGACGAACATCTGTTGAGTAA	AAGGAAAAAACTTTAGTTGCACTTTCTTAT	Y
NM_020831	57591	ENSG00000196588	MKL1	22	1282	CACTGGGATTCCTGCTTGTAG	GCTGCAGAGCTGTATTCAGGTC	Y
NM_001991	2145	ENSG00000108799	EZH1	17	2437	AGGGAGACCGACGTCCTTTAG	ACTGGCTCTTGCCAGAACC	Y
NM_144685	147746	ENSG00000160396	HIPK4	19	496	GTCACCGGGCACCACTGA	CCTAGTGTGACAGCAGCCCA	Y
NM_032353	84313	ENSG00000131475	VPS25	17	710	GGCGTCAAGTTCTTCTAG	CCCACAGGGCCCTGGAGG	Y
NM_032387	65266	ENSG00000126562	WNK4	17	558	CTTCATCACTTTTTCTTTTCCCTCCAGTGA	CATTAACTGATGGCAGGAAGCCAGA	Y
NM_144508	57082	ENSG00000137812	CASC5	15	675	CAGGACTGCCATTTCTACCACTAG	CGCAAGCCACCATGACTG	Y
NM_198494	339559	ENSG00000187815	ZNF642	1	464	GCCTTCCGAAATAAGGTGTAA	TGACTTCTGGCAAGTGATCCTCCT	Y
NM_152373	127396	ENSG00000117010	ZNF684	1	889	CTTATTGTACATCAGAAAATTCATACATAA	TTGCAGTGAGCCGAGATCACA	
NM_133639	171177	ENSG00000104140	RHOV	15	1024	AAGAAGTTCTTCTGCTTCGTTTGA	ATTACACTGCCCCCACCC	Y
NM_007299	672	LRG_292	BRCA1	17	1669	AATTGGGCAGATGTGTGA	CAGGAAATACAAAAGGTATTTAAGCTGCCT	Y
NM_024876	79934	ENSG00000123815	ADCK4	19	686	TGGGTGGATCCCTCATGA	GGCGAGAAGGAGGTTCCC	Y
NM_019074	54567	ENSG00000128917	DLL4	15	1206	GCTTGCTCCCAGGTATAA	ACATGCCAAATCCAAGTGTCTGC	Y
NM_001142590	4802	ENSG00000066136	NFYC	1	955	CAGGTGACCGGCGACTGA	AGGTACACAGCATTAAGTTCCCTTAGCT	Y
NM_004596	6626	ENSG00000077312	SNRPA	19	405	AAGATCTCCTTTGCCAAGAAGTAG	TTGTAACCTGAGTTTGAACAATATTACTAC	Y
NM_017553	54617	ENSG00000128908	INOC1	15	1522	CCCTCTGGAGGACGGTAA	GAAACACCCACAGTCCACTCCTT	Y
NM_001098209	1499	ENSG00000168036	CTNNB1	3	1286	TGGTTTGATACTGACCTGTAA	AAGTAGGGCCCTCTCTATCGCTA	Y
NM_017886	54986	ENSG00000168038	ULK4	3	510	CAAGCCGTTGGGCACTAG	AGGTAAAAGGGGAGAAGACAAGAG	Y
NM_001031694	22955	ENSG00000010803	SCMH1	1	1175	AAGCAGGGCAAGTTCTGA	CCCACCTTGGCGTCCCAAA	
NM_172373	1997	ENSG00000120690	ELF1	13	1686	CTGCTGGAACCCAACTCTTTTTAG	TCTCAAAAACAAAACAAAACAAAAGTGTGT	
NM_001429	2033	ENSG00000100393	EP300	22	1301	CTCTCACAGAGTACACTAGACATACACTAG	CAAGATCTACTTGTGAATACATTATACGTA	
NM_004941	1659	ENSG00000067596	DHX8	17	645	GCTTTCCGACGGCGCTGA	AAGAGGCCCTGCGTTCTG	Y
NM_001079675	2118	ENSG00000175832	ETV4	17	856	AAGGGTGGCTACTCTTACTAG	GAAACAGTCAGGTGAACTGCCTTTTTT	Y
NM_005586	4188	ENSG00000112559	MDFI	6	849	CTCTGCTTCTCCTCCTGA	GGGCAGTTTCTGGGGTAAGAAAG	Y
NM_031488	83746	ENSG00000100395	L3MBTL2	22	1200	AAGCAGGAAACAGACGACTGA	ACAGGGAACTTGAGCTGATGACA	Y
NM_001167827	7942	ENSG00000112561	TFEB	6	801	GAGGGCGATGTGCTGTGA	TGAGGCGTGTGACAAGCGT	Y
NM_004527	4222	ENSG00000005102	MEOX1	17	1700	GCCTCTCCAAGTTCAGAGTGA	TGTACCCACCTTTTGAAAAACTAAAGGG	Y
NM_013999	4222	ENSG00000005102	MEOX1	17	1737	CAATGGGCAGGACCCTGA	TGTACCCACCTTTTGAAAAACTAAAGGG	
NM_003924	8929	LRG_513	PHOX2B	4	1905	TTAGTGAAGAGCAGTATGTTCTGA	TCCAGAGCCCAGTGAGGTTCT	
NM_001699	558	ENSG00000167601	AXL	19	2042	CAGGAGGATGGTGCCTGA	TTTTGTCTATCTGTGTGTTCATTTCTACCC	Y
NM_001135685	4058	ENSG00000062524	LTK	15	534	AATCCCACTTATCGCTCCTGA	TTCCGGTCTAACGTCTCACACC	Y
NM_032758	84844	ENSG00000100410	PHF5A	22	861	AAATACGGCTTCAAGAAGAGGTGA	CTATAATGAAAGGTATGGGTGGTTTTGAAT	Y
NM_006293	7301	ENSG00000092445	TYRO3	15	1242	CTGCCACACACAGTAGCTGTTAG	AATCCCAGCTACTCTGGTGACTGA	Y
NM_004275	9477	ENSG00000124641	TRFP	6	1898	GTGGCTGGGATTCGTTAG	ACCACGATCAACTTCTGGAGATTTTCA	Y
NM_001136017	896	ENSG00000112576	CCND3	6	1187	GATGTCACAGCCATACACCTGTAG	GAACTGACTATATTTGGGTCAGAGACCATG	Y
NM_014460	27254	ENSG00000172346	CSDC2	22	1951	CAGGTCGTGGGCTCCTAG	CAGAGAGGGCAGGGACTTG	Y
NM_001469	2547	ENSG00000196419	XRCC6	22	413	AAGCACTTCCAGGACTGA	CTGACCCGTCACACTCTCAC	Y
NM_005474	10014	ENSG00000108840	HDAC5	17	1803	CAGGAGCCTGCCCTGTGA	TACAGGACAGATCTTGCGCC	Y
NM_016276	10110	ENSG00000101049	SGK2	20	797	GATGATGACATCTTGGATTGCTAG	TTTCAGGTACTCTTAAGTGGAGGTAGTTAT	Y
NM_001042646	22906	ENSG00000182606	TRAK1	3	2333	TCCAAACAAACTAGCTTACGGTGA	TTCATAAAGGTATGTATAATTCACAGCAGG	Y
NM_014233	7343	ENSG00000108312	UBTF	17	2389	TCAGACTCTGACTCCAACTGA	AACAATGTGAAGGTAGGTTGCCTATG	Y

NM_138370	91461	ENSG00000162878	LOC91461	2	1020	TATGTGAAGGCCTCTGGCTGA	GGGTGGGAGCAGGGCCTG	Y
NM_004599	6721	ENSG00000198911	SREBF2	22	891	GCCATTGCCGCCTCCTGA	TGGGGTAGGGGGAGGAGG	
NM_002466	4605	ENSG00000101057	MYBL2	20	575	ACCCTCATCTTGTCCTGA	AAACAGGGTCAAGGGCTCAG	Y
NM_001040283	63946	ENSG00000142025	DMRTC2	19	813	ATCTCCCTCCTGAGCTAG	CTCAGTTTGCCAGGGTCCATCT	Y
NM_181492	6942	ENSG00000281897	TCF20	22	1508	CAGGTGAGACTGTGGAGATGA	TTTGCTTCCCGGCTGCGT	
NM_022752	64763	ENSG00000105732	ZNF574	19	451	GTCCAGATCAGTGGCTGA	GCCCTGCTATGAGAGCCATG	Y
NM_001207026	5452	ENSG00000028277	POU2F2	19	417	AACCCCAGCACGGGGTAA	CCATCTCCCCATCGGTCCC	Y
NM_199003	55145	ENSG00000131931	THAP1	8	1752	TTGATGCTGCTATTGGATTACTAA	CCCGCCCAACTTCAAGTAATCTTCTAAT	Y
NM_001098798	84969	ENSG00000124191	TOX2	20	1108	AAATCGCTCTACCTCACCTAA	CAGAGTGGTCCCATCACCCA	Y
NM_033114	85437	ENSG00000139168	ZCRB1	12	1161	TTCAGTGATGAGGAAGAACTTAGTGATTAA	ACAAACCAAAACTGTTGTGGTATTTGACTA	Y
NM_133444	116115	ENSG00000167625	ZNF526	19	1965	GACACGGCCTTCGTGTGA	CACAAGTTTCAGGTCTTAATCACTCAGAAT	Y
NM_019884	2931	ENSG00000105723	GSK3A	19	788	ACCCTCACTAACTCCTCCTGA	GCAGAGAAAGCCACGGTGTGA	Y
NM_006494	2077	ENSG00000105722	ERF	19	1080	GAGCACCGAGACTCCTGA	GCCTGGATTTGGCCCATTCTGATTA	Y
NM_015125	23152	ENSG00000079432	CIC	19	786	GCTGCCACAGGCAGGTGA	GGACCTGGCCTATGGGGTT	Y
NM_032237	84197	ENSG00000185900	FLJ23356	8	483	CAGGCAAGAGAGATGCTGTGA	TAGTGGCCCATGCCTGTAATC	Y
NM_032311	84271	ENSG00000100227	POLDIP3	22	2250	ACAGAATTCAAAATCAAGCTTTGA	CTAATTGGGAGGCTGAGGTGGG	
NM_173500	146057	ENSG00000128881	TTBK2	15	1631	GCCAGTAAACTCAGCAGATAG	GTTATATGCCTCAATAGTGTTTTGAGTTTG	Y
NM_001030004	3172	LRG_483	HNF4A	20	437	TTTGGGCAAGTTGCTTAA	TTTCTTCTTCTTGGAATATTCCTCTCTTGT	
NM_003131	6722	ENSG00000112658	SRF	6	2496	AGCACCAAGAGTGAATGA	ATAGAATTAAACAGCCCAAAGCACACCTTA	Y
NM_004559	4904	ENSG00000065978	YBX1	1	1514	CAGGGCGGGGCTGAGTAA	CCACTTAAAGGATTAGAGATTTTGAGACAT	Y
NM_003432	7690	ENSG00000172262	ZNF131	5	768	ACAGCTCTGCCAGTTTTAGAATGA	ACTGGTGGTTGTCATTATAACCAAAAATAT	Y
NM_015911	51058	ENSG00000164011	ZNF691	1	842	GGGAAAGATTCCAGCTGA	CTCAAAGGCCCCAGAAACTCAGT	Y
NM_003954	9020	ENSG00000006062	MAP3K14	17	1695	CTGGAGAACAGGCCCTAA	AGTGGTGGGAAAGCAGCC	Y
NM_006887	678	ENSG00000152518	ZFP36L2	2	2097	TCCATCTCCGACGACTGA	TGAGAGGACGCAGGCTCTTCT	Y
NM_003404	7529	ENSG00000166913	YWHAB	20	2262	GGGGAGGGAGAGAACTAA	GGATAACACCTTGACTTCTGGTCCTAAATA	Y
NM_001124756	80336	ENSG00000101104	PABPC1L	20	759	AAGGCGTACATGCACTGA	AACACTTCTCCTGCTCTTGTGTATATG	Y
NM_020630	5979	LRG_518	RET	10	930	AGAATTTCCCATGCATTTACTAGATTCTAG	TCGTCAGCACAAATGGAGTGTG	Y
NM_001171627	7422	ENSG00000112715	VEGFA	6	2120	GTTTTCCATTTCCCTCAGATGTGA	ACAACAAGGTGGGTCACCC	
NM_017993	55068	ENSG00000120658	PIG38	13	640	TGTGCCTTTGAAGGAATTAAAACTACCTAA	CCAATCATATTTTCTGCCTCTTCAATAAGG	Y
NM_001253357	7075	ENSG00000066056	TIE1	1	568	ACAGCTGAGGAGGCCTGA	CTCAAGCTCCTGACCTCGTGAT	Y
NM_004966	3185	ENSG00000169813	HNRPF	10	1200	AGCATGGGTGGCTATGACTAG	GTATGCTATATATAGAGCTGCCTGAATTTC	Y
NM_014276	11317	ENSG00000124232	RBPSUHL	20	1045	TTCCACCTCTTCATCCAGACTTAG	CCTCCTCTAGAAAACGTCCAAAAGGTA	Y
NM_002999	6385	ENSG00000124145	SDC4	20	2155	AATGAGTTCTACGCGTGA	CTCTGCCCCCACCCTCTG	Y
NM_174945	284346	ENSG00000176472	ZNF575	19	625	AAGGGGGAGAGAGACTGA	TATAGCGCTTCCTTTTTTCCTATGGCTA	Y
NM_145312	220992	ENSG00000198298	ZNF485	10	715	AATAAAGAAAGAGCCATGAAATGTAGTTAG	CAGCTACTCCAGAGACTGAAGTGG	Y
NM_001129	165	ENSG00000106624	AEBP1	7	479	ACAGTGAACTTTGGGGACTTCTGA	AAGCCTGAGCACTTGCTGATG	Y
NM_014663	9682	ENSG00000066135	JMJD2A	1	1328	TACCGGGCCATCATGGAGTAG	TGACAAGCAAAGGACCCACTCTC	Y
NM_000162	2645	ENSG00000106633	GCK	7	1045	TGTATGCTGGGCCAGTGA	ACTCCTCACACTCCCCTTG	Y
NM_004556	4794	ENSG00000146232	NFKBIE	6	1233	CTGCTGTGTACCGACTGA	AATGCTCTGCCCCCATCC	Y
NM_181845	284349	ENSG00000167637	ZNF283	19	437	GAGAAAATTGATACTGATGAAACCTTATGA	CACAGCAGTATGAATTCTCTGATGTGCA	
NM_003425	7596	ENSG00000124459	ZNF45	19	943	TCAGAGGATTCACACAGGAAAACTCGATAA	TTTTGTCAAATGTTTTCTCTGAATCTACTG	
NM_022095	63925	ENSG00000198026	ZNF335	20	480	ACCCTGGCCGATGACTGA	TCATTGCCAGGCCTCTGCA	Y
NM_018651	55888	ENSG00000281894	ZNF167	3	963	GGTCTGGCTTGGTCAGTTTCTTAA	AGACTTCCAGGAGTATATGTAAAAGATATC	Y
NM_173658	285349	ENSG00000280661	ZNF660	3	1056	AAACATAATGAGGAGAAAGAAACCTCATAA	AGAACATGGATATCTCCAACCCCAAAG	
NM_001144824	10780	ENSG00000263002	ZNF234	19	2370	GGAGGAAGTTCTACAAGGTGA	GCCCATATTTTTTCTTTATCCTGTCATTAC	Y
NM_001146220	7769	ENSG00000167380	ZNF226	19	576	CTTTTGCTCTATGACCTGGCTCAAACTTAA	TGTTGCCAGATTTGCCAACAAGAAA	Y
NM_001024855	10168	ENSG00000281709	ZNF197	3	2126	AGGAAATACAGGAGGCAGAGGAACAAATAA	ATGGGGCAGAAGCGCTTAG	Y
NM_020967	57727	ENSG00000124160	NCOA5	20	1493	GGATCTTACCAGAGGCATTACTGA	AAGGCAGGGTTCAAAGAGGTTTGA	Y
NM_003420	7584	ENSG00000281306	ZNF35	3	1024	CGACACCATAGAACCCATCTTGTTGAATAA	TCCACCTCCCCTCTCCCAT	Y
NM_001258280	115560	ENSG00000281661	ZNF501	3	2026	AGGCTTCATGCTGGAGAGTAA	CCTGAGAATTACCTTATAAATATCTTGCAC	Y
NM_181756	353355	ENSG00000159915	ZNF233	19	824	TTGTCTTCAGATTCATCAGAGAGTCCATGA	CCCGCCAAAGAAACATAGCACA	Y
NM_004234	9310	ENSG00000159917	ZNF235	19	1050	CACACTGGAGGGAATCTGTAG	CTGCCACTTCCTTTTAAGGGGATAAATGTG	Y
NM_174929	83637	ENSG00000122515	ZMIZ2	7	2437	CTTTCTCTGTTTGAGAACAACTGA	GCCACCACTGACCCTGAG	Y
NM_013380	7771	ENSG00000062370	ZNF228	19	1060	CACAGAAATGAAGATTCTGTTTTGTTTTGA	GAAGTATGGGAATGGAAAACTTGACAAAAT	Y
NM_173354	150094	ENSG00000142178	SNF1LK	21	2404	TTTGTCCTGGTGCAGTGA	TGAGGTGGAATGCAGGAGTTG	Y
NM_003758	8669	ENSG00000104131	EIF3S1	15	1834	TATGTACAAGACTATGAAGACTTCATGTGA	TTCCTATTTAAAATAGATTGATTTTAGATG	
NM_152354	26974	ENSG00000267508	ZNF285A	19	1006	AGACTACATGAGCAGAGAGAAACATTATAA	CTTGAAGATTTAAAAGTTTTGAACTCTGAA	Y
NM_014518	7772	ENSG00000278318	ZNF229	19	2232	GTGCATTTAGGCGAGAACCCTTATAAGTAG	AAACACCGTGGCTCCTAATTATCTAGACTG	Y
NM_013256	7733	ENSG00000167384	ZNF180	19	940	ACTGAAGAGAAACTCTATGAATGTAACTAG	TCAATGAGGAATGTTCCTAGGAATCTGTGT	
NM_001243799	8848	ENSG00000102804	TSC22D1	13	2002	GTTTTGCTTATTGTATATTTGTGTATGTAG	TAATCAGTTATATAATAACCCACAGGCCAA	
NM_001270497	55713	ENSG00000198185	ZNF334	20	1846	CTTTTACATCAGAAATCCCACAAGGAATAA	GTCTTGAAATTTGGTGTCTTATTGTGCTCT	Y

NM_005413	6496	ENSG00000138083	SIX3	2	1497	GACTCGGAATGTGATGTATGA	CCTGTTCTGGTCTTCCCAATGC	Y
NM_016932	10736	ENSG00000170577	SIX2	2	1165	GTGGACCTGGGCTCCTAG	ACCTAGCGACTGACACAAGTCG	Y
NM_005178	602	ENSG00000069399	BCL3	19	609	GCTCCAGGAGGCAGCTGA	AGAAGTCCCAGAGTGACACACAG	
NM_001004329	440097	ENSG00000185610	DBX2	12	1710	GTACTTACTGGGGCTGTCTGA	GCACATAGGAAGTACCCTATAAATGTAAAT	Y
NM_006963	7570	ENSG00000165512	ZNF22	10	1466	TCTGTGGCTGGTCTCCGTTAA	TGCACTCAAATGCAAGGCCA	Y
NM_001039360	201501	ENSG00000184828	ZBTB7C	18	2172	ATGTCCGAAGCCAACAACTAG	AAAAAATTGAAGCAAAACAAAAACACAACC	
NM_018079	55133	ENSG00000068784	SRBD1	2	810	GACCTCATTCGGGTGTTATGA	GAAAGGACACACTCTTCTATGCTAAATCAA	Y
NM_025077	114034	ENSG00000132773	TOE1	1	456	ACTTGGGGCAGTAGCTGA	GCTTCTATGTATTTATCAACATGTGATACA	Y
NM_007170	10420	ENSG00000070759	TESK2	1	1137	GGAAAGCAGGATGGGTGA	CAGAGGTATTTTGGGTCTCTCTAGCCT	
NM_005244	2139	ENSG00000064655	EYA2	20	891	CTGGAGCTGGAGTATTTATAG	TTCTCCTTCCTCCCTCCCC	Y
NM_013351	30009	ENSG00000073861	TBX21	17	933	GGACAGTTTTATAACTATTTTCCCAACTGA	TGCTGTAACTCAGCTGGAGTCATTT	
NM_000400	2068	LRG_461	ERCC2	19	418	ATTGCTCAGCAGCTCTGA	CTTGCCCCTCACCTCCCC	Y
NM_006663	10848	ENSG00000104881	PPP1R13L	19	736	AAGCCTCAAAGGAGTAAAGTCTAG	GTGCCCCAGCCTGGGCAC	
NM_001114171	2354	ENSG00000125740	FOSB	19	2347	TCCCTCCTCGCTCTGTGA	CCTGCATAGGTGTGTGTCTGTGT	
NM_003110	6668	ENSG00000167182	SP2	17	1313	CTGGTCACGAAGAACTTGTAA	GGGCATGTGGCTTGGGTTT	Y
NM_001195193	4678	ENSG00000132780	NASP	1	945	ACAGTTGAAAGCACTGCATGTTAA	AGCCTCCTGAGGCAGGATAATCT	Y
NM_003204	4779	ENSG00000082641	NFE2L1	17	2084	AAGGACCGGAGAAAGTGA	TATGAAGTACATGGTGTCTAATGCCTG	Y
NM_006807	10951	ENSG00000108468	CBX1	17	1563	GATGATGACAAAAAAGATGACAAGAACTAA	AACATGGTGTGACCACTTAAAGATTCAG	Y
NM_175875	147912	ENSG00000177045	SIX5	19	896	GAGCCCTTGGAACTGTGA	CTCAGTGCCACCCCCTCC	Y
NM_004339	754	ENSG00000183255	PTTG1IP	21	2176	AACCCGTATGCTAGATTTGAAAACAACTAA	GTTCTGACAGCCACAGGCATTTAGT	
NM_052854	90993	ENSG00000157613	CREB3L1	11	856	ACCACCATCAAACTCTCCTAG	CTACCTCTACAAAAAAAAAAAAATGCCTTC	Y
NM_001190417	641339	ENSG00000251192	ZNF674	X	2298	AGAAGTCACACAGGAGATAAAAACCTATGA	CAAACCTGTAAACTCAATCATATGGAAAAC	
NM_004497	3171	ENSG00000170608	FOXA3	19	919	TCTTTGCTTAATGCATCCTAG	CAAAGAGCCAAAGGTCAGGTCGAT	Y
NM_001012333	4192	ENSG00000110492	MDK	11	523	AAAGGGAAGGGAAAGGACTAG	CTGGAAGAAATGTTTGTAATTGTTCTGCTT	Y
NM_002516	4858	ENSG00000104967	NOVA2	19	530	CCCCAGAAAGTGGGATGA	AAAGCAGAGAGGTGGGGTGTT	Y
NM_005904	4092	ENSG00000101665	SMAD7	18	1699	GAGGTCATCTTCAACAGCCGGTAG	CTGTTCTAGATGTCTCTTTTGTGGCC	Y
NM_015070	23091	ENSG00000123200	ZC3H13	13	1823	AAACCCAAAGGTAGTTTCATTTTACTTTAA	TCTTTCATGTTGGTACACTTGAATAGACAA	
NM_002145	3212	ENSG00000173917	HOXB2	17	591	GACCTGCAGTTTCCCTAA	CACCTTTGTACCACCCGGACTT	Y
NM_002146	3213	ENSG00000120093	HOXB3	17	1644	AAATTAACACACCTGTGA	GACATATCAAGGGGGAGACAGGAA	
NM_207327	150383	ENSG00000205643	LOC150383	22	1259	CCCGGTTCTCGGACGTGA	TGGAGGCTGTCTCCACCT	Y
NM_018952	3216	ENSG00000108511	HOXB6	17	856	GAAGAAAAACAGGCCGAGTGA	CGCTCCAGCCTTTATCTCAGCT	Y
NM_004502	3217	ENSG00000260027	HOXB7	17	789	GAGGAGGAAGAGGAAGAGTGA	CCAGAGCAGGAGGCACCAA	Y
NM_024016	3218	ENSG00000120068	HOXB8	17	1036	AAGGGCGACAAGAAGTAG	CCTGGGCCGAGGGACTCG	
NM_024017	3219	ENSG00000170689	HOXB9	17	1923	AAAATGAATAAGGAGCAGGGCAAAGAGTAA	GAGCTCTGCAGGGGACCTT	Y
NM_006361	10481	LRG_771	HOXB13	17	2205	AAGAACAGCGCTACCCCTTAA	CCCAGGCCGCTGGAAGCC	Y
NM_172225	127343	ENSG00000197587	DMBX1	1	1900	GATACGCTGCCCAACTGA	AAAACTTCTGTGTTACAAGGTTGCGA	Y
NM_007241	11267	ENSG00000159210	SNF8	17	558	AGAGAAGCCCTCCCCTGA	TTTTCTTTCCTCCAATTTAGCCACGATTGG	Y
NM_014159	29072	ENSG00000181555	SETD2	3	865	AAACCCAAAGAGGACACTGAATTAGAGTGA	ATGTCTGCCCCAGTCATAGTAGGA	Y
NM_001251935	10062	ENSG00000025434	NR1H3	11	517	ATCTGGGATGTGCACGAATGA	GATCGCAGGCTTCCTCCTCTATTA	Y
NM_153380	7592	ENSG00000147124	ZNF41	X	1448	AAACGCTATAAAGCCAGTGACTGA	GCTTCTGGGGTGCTGGTCAT	Y
NM_020528	54039	ENSG00000183570	PCBP3	21	928	GGGATGGGCACGCTGTAA	TGTTGTGGAAAGGGGGAATGAAATG	Y
NM_001080547	6688	ENSG00000066336	SPI1	11	549	CGCCACCCGCCCCACTGA	TGTGTCATTGGTCCTCATGTTCC	Y
NM_001654	369	ENSG00000078061	ARAF	X	644	GCCCGCCTTGTGCCTTAG	ATGTGGTCCTCGTTCATCGTG	Y
NM_002634	5245	ENSG00000167085	PHB	17	1114	CTCCAGCTGCCCCAGTGA	CACAAAGTGTTTTCTGAAAACAAAGCATTT	Y
NM_005229	2002	ENSG00000126767	ELK1	X	1489	GGGCCCCAGAAGCCATGA	GATATGCAGTCCCTACTATTGTTTCTCACA	Y
NM_015168	23211	ENSG00000130749	ZC3H4	19	2346	TCCCCCTTTTGCCAGTAG	CAAATTGTCCCTCAGTGTCCTCATGTTA	Y
NM_001204141	4152	ENSG00000141644	MBD1	18	886	TCCGTCCTGGTTCCCTGA	AAAGCTCTAATTACAATTCCAGTTGACTTT	Y
NM_006962	7569	ENSG00000147118	ZNF182	X	1496	AAGTCAAAGTTCATGGCACATTAG	GTGCCTCTGGAATTGATTGAATAACTGTTG	Y
NM_152995	152518	ENSG00000170448	NFXL1	4	1102	TACATCACCCATGATGTCAATTAA	AACATATCTACTTTTTAGATACTTTTAGGT	
NM_021035	57169	ENSG00000124201	ZNFX1	20	1545	GAGGAGATCCAGGGGATGATGTAG	TAAAAATGATTCATTCACCAGCTAAATAAA	Y
NM_012186	2301	ENSG00000186790	FOXE3	1	957	CTGGAGCGCTACCTGTGA	CCTGCTGGAGAATCACTTCCTAATC	Y
NM_004474	2306	ENSG00000186564	FOXD2	1	1248	AGTGGCTGCCACTTCTGA	CTTGCATCCTCTAAGCTCAAACCC	Y
NM_001190255	57232	ENSG00000221994	ZNF630	X	470	TATAGGAAAGACACCTTGTATATATGCTGA	GCCTGGGTAACAGAGCGAGAC	Y
NM_001934	1748	ENSG00000108813	DLX4	17	1196	TCGCCTCAGATGATGTGA	ACAGAGCTAGTTAGTGGCCAAGACAA	Y
NM_005220	1747	ENSG00000064195	DLX3	17	1692	CCTGGGGCTGTGTACTGA	GGCCAGGCCCAGCCCAGC	
NM_005635	6756	ENSG00000126752	SSX1	X	1237	CCTGAGGAAGATGACGAGTAA	GCGCTCAGCACGAGGACA	Y
NM_003215	7006	ENSG00000135605	TEC	4	1813	GTTGAATGTGAAGAAACTTTTGGAAGATAA	GTGCAGCAAACCACCATGG	
NM_015401	51564	ENSG00000061273	HDAC7A	12	1297	GTGGAGGAGGAAGAACCTATGAATCTCTAA	AGGGCAGATTGTTAAAGGGGCT	Y
NM_001257359	201191	ENSG00000167100	SAMD14	17	1764	GAGGCCAAGAAGAGCTAG	TCCGGCCCAGCTCAGGAC	Y
NM_001789	993	ENSG00000164045	CDC25A	3	1947	TACAGTCGTCTGAAGAAGCTCTGA	TCTTGCAAATCAATCTGAACCACAAGTTTT	Y
NM_005636	6759	ENSG00000268009	SSX4	X	1554	CCTGAGGAAGATGACGAGTAA	GTTCTCAGCACTAGTACACACTCATTAGTT	Y

NM_002747	5596	ENSG00000141639	MAPK4	18	2152	TTCTCCAAAGAAAGGTGGTGA	ATTACCAATCTCTGTAGATTGTCCCCTC	
NM_198479	284355	ENSG00000178928	TPRX1	19	712	TCAGGCCCCAGGTTATTACTGGATTTATAG	CATTGTGTTTTACAACATTATCAGTCTGCC	Y
NM_016089	51385	ENSG00000164048	ZNF589	3	2383	AAGCCTTATGTGTGCAGAGATTGA	AACTAACAACTCCAGATGCGCTG	Y
NM_006579	10682	ENSG00000147155	EBP	X	439	AAAGCCAAGAGCAAGAAGAACTGA	TCTGGCGGGGGGTCAGTG	Y
NM_001006610	6477	ENSG00000196470	SIAH1	16	1225	GGCATCAATGTAACTATTTCCATGTGTTGA	CCCAGTTATTTGCTGTGATTAGAGTTAAGT	Y
NM_003173	6839	ENSG00000101945	SUV39H1	X	1625	TGCCGCAAATACCTCTTCTAG	AGATGATGGTGTAAGTGATATGCTGTG	Y
NM_005985	6615	ENSG00000124216	SNAI1	20	1007	TCAGGATGTCCCCGCTGA	GTGACACCCAGGACTCCAAAG	Y
NM_001270629	29079	ENSG00000136146	MED4	13	1591	AGCAGTAGTAGTGAGTCTGATTGA	TGAGCCAAGTTTGTGGTAATTTGTTACG	Y
NM_006044	10013	ENSG00000094631	HDAC6	X	538	GATATGCCCCACCCACACTAA	TTCAGTTAGTTTTTTGGGGGCAATGGA	Y
NM_001081640	5591	LRG_162	PRKDC	8	1245	TGGGAGCCCTGGATGTGA	TGTACGCCATTGTGCCTGG	
NM_016626	51320	ENSG00000176624	RKHD2	18	1981	GTTACTCAGGCAATCCAAATTCACTCTTAA	AACCAAGAGCACACACACACAAAATAACTA	Y
NM_006875	11040	ENSG00000102096	PIM2	X	1129	TGGTCCTTGCTACCCTAA	GTGTGTGTGTGGTGCAGCTTC	Y
NM_005194	1051	ENSG00000172216	CEBPB	20	799	TCCTCCGGCCACTGCTAG	CAGAAAAACCAGCTGCAACACC	
NM_006521	7030	ENSG00000068323	TFE3	X	1607	AGCATGGAAGAGGAGTCCTGA	TTTGCATATTGTCTTCCTCCATGCC	Y
NM_005914	4173	ENSG00000104738	MCM4	8	1561	ACCGTGCGCTTGCTCTGA	TCCTGACCTCATGATCCAGCCAT	Y
NM_000321	5925	LRG_517	RB1	13	1999	ATGGATACCTCAAACAAGGAAGAGAAATGA	GAAGAAATAAATTTGGAAATCTCTAGCATA	
NM_014009	50943	LRG_62	FOXP3	X	1078	CCTACACCTGGCCCCTGA	CCCAGCCTCAGGCAGGGA	
NM_001664	387	ENSG00000067560	RHOA	3	1243	AAATCTGGGTGCCTTGTCTTGTGA	CCAGATTAGCTGCTGGGTGG	
NM_018132	55166	ENSG00000031691	CENPQ	6	1012	GAAGCCTATAAGAAACTGGATGCATCTTAA	TGAGCTGCCCCAGGGTCC	Y
NM_015339	23394	ENSG00000101126	ADNP	20	1239	AGCAGCCAACAGGCCTAA	AAAGCATTCTATTTGCAGATGACAGATCTT	
NM_015069	23090	ENSG00000102935	ZNF423	16	851	AGCCAGCACGCACAGTGA	CGATTCTTTTCCAAGGGGAGCAG	Y
NM_003068	6591	ENSG00000019549	SNAI2	8	1307	TGCTGTGTAGCACACTGA	TTTTTGTTAAAGTGGTATGCTAACAAACCA	Y
NM_001256659	8463	ENSG00000074219	TEAD2	19	908	CGCCTGGTCAGGGACTGA	CCCTGGGGACCCACGTGG	Y
NM_024046	79012	ENSG00000164076	CAMKV	3	1509	AGGGAGGAGGCCAGCTGA	TCACCCTCAGCCCAAGAAATC	Y
NM_014577	23774	ENSG00000100425	BRD1	22	1124	GACCTCAGTGACATTGACTGA	ATATGATCAGGTGAAATAACAAATAATGCC	Y
NM_002070	2771	ENSG00000114353	GNAI2	3	1844	GACTGCGGCCTCTTCTGA	TGAAGCTCAGAGCGTGGG	Y
NM_001001852	415116	ENSG00000198355	PIM3	22	1125	AGCAGCGAGAGCTTGTGA	TGGCACCCAGCTGGGCCT	Y
NM_007254	11284	ENSG00000039650	PNKP	19	225	CAGTTCTCCGAGGGCTGA	TCATGCCAGGTCCCAGCT	Y
NM_001193357	23636	ENSG00000213024	NUP62	19	1592	TTCCGGATCACCTTTGACTGA	TTCATCGCATACCCCTATGTCTATGCA	Y
NM_001193646	22809	ENSG00000169136	ATF5	19	1024	AGGACCCGTAGCTGCTAG	GTATTTTCAGTAGAGATGGGGTTTCACCTT	Y
NM_139071	6602	ENSG00000066117	SMARCD1	12	1891	CTGGGAATCCGGAATACATAG	GCAGACAGCAATCTCTTAAGGTAAGAC	Y
NM_005254	NULL	NULL	GABPB1	15	1620	CGTCTTCAGACTAATAAAGAAGCTGTTTAA	ATCAGGCTGACAGACCATAGACC	Y
NM_001164106	54456	ENSG00000073146	MOV10L1	22	418	CATCAGGAGCCCAGCTGA	CTGGCAGCCTTGCCTGGA	Y
NM_001164105	54456	ENSG00000073146	MOV10L1	22	404	CTGATCTGCAGTGGCTGA	CTGGCAGCCTTGCCTGGA	Y
NM_001144775	1996	ENSG00000162374	ELAVL4	1	870	AAAGCCCACAAGTCCTGA	AGTATGAACGCGACAAAGTTGTCAATTTCC	Y
NM_001243926	7867	ENSG00000114738	MAPKAPK3	3	1431	GGCTGCAACAACCAGTAG	CCTGGCGAGGACTCTGGC	Y
NM_002202	3670	ENSG00000016082	ISL1	5	1299	GCCAGTCCTATTGAGGCATGA	ACCCAAACATGCAAACTCGACCTTAA	Y
NM_002969	6300	ENSG00000188130	MAPK12	22	679	AAGGAGACGCCTCTGTGA	GGCACCAGCTTTGTGGCC	Y
NM_002751	5600	ENSG00000185386	MAPK11	22	1415	CTGGAGATTGAGCAGTGA	CCAGAAGATGGCCGCTGGT	
NM_199426	55734	ENSG00000020256	ZFP64	20	1048	GACTCCATTCCAGATTAG	TATTTAATGTGACTGTGGAGGAGGTAAGAT	Y
NM_032110	63950	ENSG00000142700	DMRTA2	1	1294	GCTCCGGAGAAGCAGTAG	TCTCCTCTCCCAGTAGCCC	Y
NM_001256647	7376	ENSG00000131408	NR1H2	19	606	TGGGACGTCCACGAGTGA	ACTTTCACAGCAGGCGACACA	Y
NM_006575	11183	ENSG00000012983	MAP4K5	14	1706	ATCTTGGCTGGACATGAAAATAGTTACTAA	GATTCAGTAAGAAAAGGAGGATTCTGGCTT	Y
NM_002968	6299	LRG_674	SALL1	16	1317	AAGGAGATCGTCACGAGTTAA	CGCAGGCCCATGCAAAGC	Y
NM_005171	466	ENSG00000123268	ATF1	12	1561	AAGGATCTTTATTCCAATAAAAGTGTTTGA	TCATAAAGTGCTGCCAAGTCAACAGA	
NM_001171904	9730	ENSG00000145041	VPRBP	3	3082	GATGACATCATCTTATCTCTGAATGAGTGA	GATTAGAGGCTGGGGCTCACT	
NM_001262	1031	ENSG00000123080	CDKN2C	1	544	GGAGCCACAAATCTTCAATAA	ATCATCAAGAGAGTATGTCTAGTTTTCCAG	Y
NM_001173452	7024	ENSG00000135457	TFCP2	12	1665	AATGATAGCTATCATATCATACTGAAGTAG	TACGTCTCCTCCTCAAAATGGAGACA	Y
NM_001174100	57060	ENSG00000090097	PCBP4	3	787	CAGAAATTCTCCCCCTACTGA	CCCCCAAAGGGTCCTGAAG	Y
NM_007147	7728	ENSG00000105497	ZNF175	19	1451	TATTCTGTGAAAGGCTTTACCAAGCAATGA	GTGTTTGTGTGTGTATCATGTGCTG	Y
NM_002388	4172	ENSG00000112118	MCM3	6	754	GGCATCATCTTCCTCATCTGA	CTCTAGAGAAGACATGACCCCCAGA	Y
NM_002748	5597	ENSG00000069956	MAPK6	15	1445	TACAGCAGCATTCTGAAACATCTGAACTAA	TTCCATTCTAATATGTGATGGTTTAAGTCA	
NM_001135590	84765	ENSG00000161551	ZNF577	19	1414	TTGTATCTTACAGATATTGTATCAGAATAA	ACGCTGTAGTGCAGTGGCAT	Y
NM_023074	65251	ENSG00000198093	ZNF649	19	1563	GCCCACATCCTGCATTCATGA	AAAGTACCCAAAGATAAATGGAGTTTGGAA	Y
NM_001202233	3164	ENSG00000123358	NR4A1	12	743	GACACGCTGCCCTTCTGA	TATTATCCGCCTTTTACAAATGAGTACACT	Y
NM_021632	59348	ENSG00000256683	ZNF350	19	694	GTCTTATTTTATGTTACAGAAAACCCATAG	CATTTCACCTAATGTAGATATTTGCACCGC	Y
NM_001080430	27324	ENSG00000103460	TNRC9	16	1399	CAAGTATTATCGCAGGTCAGTATTTTCTGA	TCAAGGATCTTCATTTCAGGCTATGCAATG	Y
NM_198480	284370	ENSG00000197619	ZNF615	19	1726	CTTGTTAAACACAGGAGAATTCACAGGTAG	CTGAACCTTTCTATATATTGTTGGTGAGCA	Y
NM_014650	9668	ENSG00000256087	ZNF432	19	476	ACTCATAATGGAAACAAACCCTGA	GCTGCTCTACACTGCCTATATGCAT	Y
NM_178523	90317	ENSG00000204611	ZNF616	19	598	ACAACTAAACTCAATGTGACAAGGCCTTAG	TTTCTCCAGGATGTATACATCCTGGAATAT	Y
NM_199289	341676	ENSG00000197168	NEK5	13	823	GTGCTCATCCTGATGTGA	GGGAGGTGTACCCAACAGCT	Y

NM_001146099	4752	ENSG00000136098	NEK3	13	679	GGCCTGTGCGACAGATAA	CCAAGCTTCCCAGGGCTGA	Y
NM_003157	6787	ENSG00000114904	NEK4	3	1177	AAATTTTTTGAAGAAAACATGAATTTTTGA	GCACACGCCTGTGGTCTCA	Y
NM_006350	10468	ENSG00000134363	FST	5	966	CACAAGAGCGCTTTTTATCTAATTTCAGGA	AGTCAAGGTTCCTTGTAAACAAAACCC	Y
NM_001010851	90321	ENSG00000196214	ZNF766	19	1705	AGTGTTCATGAGAGAGTCCTTACAAACTGA	AAACCTGCATGTTGTGGACATGTACA	Y
NM_001145434	400713	ENSG00000221923	ZNF880	19	659	ACTGGAGAGAAACCGTACAGATGA	TCAGCCTCCCAAAAGTACTGAGATTACA	Y
NM_032423	84436	ENSG00000167555	ZNF528	19	1845	CAGCATCAGAGAGTTCATTCATGA	TAACCAAAGGACATGTTTCATTTCACAACC	Y
NM_003643	8521	ENSG00000137270	GCM1	6	1424	CTGAACCTGTGTCCTTTGAGATGA	TGCAGAGAACATTTCTAATATCTGGAATAA	Y
NM_004498	3175	ENSG00000169856	ONECUT1	15	579	TCAAGCACTTGTACCAAAGCATGA	TTGCGGGCCTGGCAGGTT	Y
NM_001039886	388558	ENSG00000198482	ZNF808	19	602	GCAATTCATGGTATAGGGAAATTTGATTAA	GGCCTGAGCCACCACACC	Y
NM_006121	3848	ENSG00000167768	KRT1	12	637	TCTACCACTTATTCCGGAGTAACCAGATAA	GGTGAAATGTTGCCTGGTTGG	Y
NM_001105554	NULL	NULL	ZNF83	19	829	GCCGGAAAGAAATCTAACACATGTAATTAA	TGGTTGGCTAGGATGGTCTCAATCT	Y
NM_030972	81856	ENSG00000213020	ZNF611	19	2304	CATAGAATTCATACTGGAGAGAAACCTTAG	TGGAGGAGCGTTCTATGCCAAT	Y
NM_001146702	8242	ENSG00000126012	KDM5C	X	1603	GTCTTCCCTCCCTGGTAG	CATGTAAACAATCTGTTGATGGTCCCTGAT	Y
NM_004187	8242	ENSG00000126012	JARID1C	X	1826	CCTCAGCAACAGTTGTGA	CATGTAAACAATCTGTTGATGGTCCCTGAT	Y
NM_212539	5580	ENSG00000163932	PRKCD	3	631	GAGCACCTCCTGGAAGATTGA	GCCTTAGGCCCAGGGTCC	Y
NM_006969	7576	ENSG00000198538	ZNF28	19	2460	CATAGACTTCATAGTGGAGAGAAACCTTGA	ACCACAGTGGAAATAAACAATCAGGGAAAA	Y
NM_199132	NULL	NULL	ZNF468	19	2373	CATAGGCTTCATAGTGGAGAGAAGCCTTAA	ACCACAGCAGAAATAACCAATCAGG	Y
NM_001417	1975	ENSG00000063046	EIF4B	12	2166	GAAAATGAGGGAGAAGATTATGCCGAATAG	GTAACCCCACCCTCTAATCTTGGACAATAT	Y
NM_015235	23283	ENSG00000177613	CSTF2T	10	2393	AAATCCACTGGAGCGTCTTGA	AATGGCTCTAAGTGAGTTCCATCCATT	Y
NM_005611	5934	ENSG00000103479	RBL2	16	1528	GACCGTGGTTCCCACTGA	GAAAAAGTGTTACAACGGTTTCTAATGAAG	
NM_001083617	9821	ENSG00000023287	RB1CC1	8	1504	AAAGCCGTATCATGGAATAAGAAAGTATAA	GAAGGGTACCAATTAAGTCAAAATGGCCTA	
NM_033288	90338	ENSG00000170949	ZNF160	19	1643	ACCGGAGAGAAACCTTACAAATGA	TATAGGTTTCCAAAACTGCGTTATTTGATT	Y
NM_000966	5916	ENSG00000172819	RARG	12	1290	CTGAAGTCCCCAGCCTGA	TGTCCCCACCACAATCCAG	Y
NM_018355	55786	ENSG00000170954	ZNF415	19	678	ACTAAGGAGAAACCTTATAAAAGAAATTAA	TCCCAGACATTGCCATGAAAGG	Y
NM_006418	10562	ENSG00000102837	OLFM4	13	1470	GTCTTGCAGAAGCCCCAGTAA	TTATAAAAGTTATTGATGTGATCTGTTGTT	Y
NM_001172675	84671	ENSG00000197937	ZNF347	19	1784	AAACCTTGCAAGCCATCACAGAATTCATAG	TGAAAACTGCACTTTCTATGGGATTTTCAT	Y
NM_152860	121340	ENSG00000170374	SP7	12	1750	AGCAACTTGCTGGAGATCTGA	CCCGGCCGATAAACTATTTTACCG	Y
NM_182609	342926	ENSG00000197928	ZNF677	19	1767	ACAAAAATTAAGTATTCAAGCTGTACCTAG	AAATTGTATACTGTTTGAACATAACATGGA	Y
NM_138374	91664	ENSG00000213799	ZNF845	19	1461	CATAGAATTCATACTGGAAAGAAACATTAG	TAAACCCGAAAGGCAGAGGCT	Y
NM_001128913	5094	ENSG00000197111	PCBP2	12	1900	GGCATGGGGAGCAGCTAG	GAATCTCACTCCATTTCTACTCTTTCTCAC	
NM_033067	63948	ENSG00000143006	DMRTB1	1	1003	CAGGAGCAGTCCGACTAG	GAGGGCAGGAACTAAAAATAGGAACTAAGC	Y
NM_001206683	11016	ENSG00000170653	ATF7	12	535	TGCTTTGGGATAATTTTCTTAATTGGTTAA	AAAAGTTCCCGAAGTGACATAGTTTATAGT	Y
NM_147193	148979	ENSG00000174332	GLIS1	1	566	TCCATCTACACAGACACCTGA	GCCACTGGTTCCCATCCTC	Y
NM_001004301	126017	ENSG00000198346	ZNF813	19	2386	TTTAATGAGTGTGGCAAAGCTTTTAATTGA	CGAACTCTGGATCTCAGGTGATCC	Y
NM_001079906	55422	ENSG00000130844	ZNF331	19	2497	CATCAGAGGATCCACAACAGTTGA	CTCTGGGCCCTGACCCCG	Y
NM_001143682	57658	ENSG00000012822	CALCOCO1	12	1005	CCCTTCACCTTTGAGTGA	CAGGACAGTAACTGCCCAGCAT	
NM_024336	79191	ENSG00000177508	IRX3	16	566	TTATCGGCTCTCTCCTCATCCTAG	ACTTCTGCGCCTCCTCAGCTAT	Y
NM_017410	3229	ENSG00000123364	HOXC13	12	1468	CATCTCCACTCCACCTGA	AGTGGAGCAAAACAAGGGTTTGGAA	Y
NM_014212	3227	ENSG00000123388	HOXC11	12	1186	TTCTCGGGAAATCCTCTGCTGTAA	CGGCTAGCACCGGCCTAT	Y
NM_017409	3226	ENSG00000180818	HOXC10	12	1012	GAACTGACCTCCAATTTTAATTTCACCTGA	TTATGGTCTCTCACTTACATTAAACCCG	Y
NM_006897	3225	ENSG00000180806	HOXC9	12	842	ACCGACAAGGAGCAGTCCTAA	CTGCAGAAGCAGACGTTTGC	Y
NM_022658	3224	ENSG00000037965	HOXC8	12	1560	GAGGAGGAAAAGGAAGAAAACAAGGACTAA	GGCTTCTCTCCACGTGAGTTTCT	Y
NM_002739	5582	LRG_669	PRKCG	19	932	CCTGTGCCCGTCATGTAA	CTCTGCTTGCTCCCCTGCA	Y
NM_130850	652	ENSG00000125378	BMP4	14	475	GGATGTGGGTGCCGCTGA	TACTTCCTGTCCCTACAACTTAACCAC	
NM_153693	3223	ENSG00000197757	HOXC6	12	1041	GAAGAGGAGAAGCAGAAAGAGTGA	GAAGGCCGGGGCCGGGCG	Y
NM_018953	3222	ENSG00000172789	HOXC5	12	1048	TCCAAAATGAAAAGCAAAGAGGCTCTTTAG	AATTTCACCTCCCTCTACTCACTG	Y
NM_153633	3221	ENSG00000198353	HOXC4	12	1005	GCAGAGGACATTACCAGGTTATAA	CCTTGCTTGTTCTTCTAAGGACATTGGAAG	
NM_031157	3178	ENSG00000135486	HNRPA1	12	1113	TATGGCAGTGGCAGAAGATTTTAA	TGGAGCGGCATTAGCCAG	Y
NM_018070	23648	ENSG00000157216	SSBP3	1	789	AGCATGACGATGAGTGTGTGA	AAGCGTGCCGCTGCTGTT	Y
NM_001009955	23648	ENSG00000157216	SSBP3	1	789	AGCATGACGATGAGTGTGTGA	AAGCGTGCCGCTGCTGTT	Y
NM_201437	6917	ENSG00000187735	TCEA1	8	1728	ATATTTTTTCTTTTTTGACAGTTCTGTTGA	CTGCTGAATACTAGTTTTTACTGAAGACCA	
NM_005853	10265	ENSG00000176842	IRX5	16	790	GAATTGAAGAAAGGTATGTCCGACATTTAA	AAACAGCCCGCATCAGTGATC	Y
NM_198988	94059	ENSG00000278312	LENG9	19	471	GAGATCCGCCTGGAGTGA	CTCTCCGCATTCTTCCCTTGGTT	Y
NM_001242903	8165	ENSG00000121057	AKAP1	17	1200	GACAGCTACTACACAAGCCTTTGA	CAGATAGAAATTTCTGGCCAAATTGTCTTC	Y
NM_003488	8165	ENSG00000121057	AKAP1	17	1205	GACAGCTACTACACAAGCCTTTGA	AAGGCCAGATAGAAATTTCTGGCCAAAT	Y
NM_003222	7022	ENSG00000087510	TFAP2C	20	1449	CTGGAGAAAATGGAGAAACACAGGAAATAA	AAACTTCAGGTTCCTCCCCAACA	Y
NM_005228	1956	LRG_304	EGFR	7	1901	AGCAGTGAATTTATTGGAGCATGA	GTCACGTTGTTTTAAGGTTAGCATCTATGA	Y
NM_024335	79190	ENSG00000159387	IRX6	16	635	TAATCAACCTTTCCTCTCTCAGCAGGTTAG	CCTTGTCTGTTACCTTGTCAGTTTCTTTCC	Y
NM_022454	64321	ENSG00000164736	SOX17	8	1081	GTATATTACTGCAACTATCCTGACGTGTGA	AACTGTTTCTTCTTGATAAAGTAATGGTGA	Y
NM_000222	3815	LRG_307	KIT	4	2338	GTGCACGACGATGTCTGA	CTGTTGAATCTCGAAGCATGTAAATCCTAG	Y
NM_198451	139628	ENSG00000189299	FOXR2	X	1721	GAGTTGTTGACCTCTCTCTTTGATCTTTGA	TGAAAGCTTGCCTTGTTTGAGAAGTTTACA	Y

NM_170721	124540	ENSG00000153944	MSI2	17	1532	GTTTCACAAGACATAATTTTTATCAACTAG	GAGGGCTGGGAACAGGCG	Y
NM_032430	84446	ENSG00000160469	BRSK1	19	647	GGGACCCCTCTGCCCTGA	AGCTGGTGGCAGCCAGCA	Y
NM_032701	84787	ENSG00000133247	SUV420H2	19	852	GGCGGTGAAGAGCTGTGA	CCTAGTCCATCACGCTCCCAAAT	Y
NM_033109	87178	ENSG00000138035	PNPT1	2	2354	ATTTCACAGTCATCATCTAATTCTCAGTGA	TGAAAAAGAAAACAGAAAATTTAAGAAAAA	Y
NM_002253	3791	ENSG00000128052	KDR	4	1862	CTGAGCTCTCCTCCTGTTTAA	TCAAAATAGTTGTGGTGATATTATACCCTC	Y
NM_183425	55544	ENSG00000132819	RBM38	20	1963	CTTGGCCCCACAGGCTGA	GCTACTGAGGCTCAGCGG	Y
NM_017495	55544	ENSG00000132819	RBM38	20	1664	CCTGACAGGATGCAGTGA	GCTACTGAGGCTCAGCGG	Y
NM_001269046	140690	ENSG00000124092	CTCFL	20	1562	CTCCTCAACACGATGGATAAGTGA	TTTTTTTTTTGAGAAACTGCCATACTGCTT	
NM_032836	84922	ENSG00000179943	FIZ1	19	1259	CACCGGGGCATGGACTGA	AGCCTAAACCGACTCTAATTTAAAAGGG	Y
NM_001195605	100507290	ENSG00000261221	ZNF865	19	1923	GCCGGGAAGGATGCCTGA	TCTGTCTCATGCTGGGTCTTCA	
NM_203374	147808	ENSG00000179922	ZNF784	19	1190	GTGGAGGCCGACCAGTAG	TGGGGCCTACTCACCCCC	Y
NM_006213	5260	ENSG00000164776	PHKG1	7	951	GCCGAGGAGGACTACTGA	TATTTAAAGCACAGTTCAGTCCTAAAAGGG	Y
NM_052947	115701	ENSG00000198796	HAK	18	753	CCAGGCGAAAAGAAAACCTAA	GTTGTAATGTAAGAATGAGGGGAAACCACA	Y
NM_016202	51157	ENSG00000213015	ZNF580	19	623	CACGTGCGCCTCCACTAA	GGGAAGGAAGGTTGGGGC	Y
NM_016535	51545	ENSG00000171425	ZNF581	19	638	CGGTGGAAGCATCCATGA	CAGATGACCCAGGTCTTCAGGA	Y
NM_001798	1017	ENSG00000123374	CDK2	12	1339	GTACCCCATCTTCGACTCTGA	TCACCGTCAGCACTAGCCAAAAT	Y
NM_003168	6827	ENSG00000213246	SUPT4H1	17	1248	AAATCCAGAGACACAGCTATAAAGACCTAG	CAACACCAGGTGGCTGAGACA	
NM_017763	54894	ENSG00000108375	RNF43	17	1446	TGTGAACAGGCTGTGTGA	TGCCAAGCCCTCTACTCTACC	Y
NM_001982	2065	ENSG00000065361	ERBB3	12	1632	GCTAATGCCCAGAGAACGTAA	GTTGTTCTAAAGAAATAGAAGTAATAGTAG	
NM_001080439	124535	ENSG00000176160	HSF5	17	2372	TTTCCAAAGGAGGAAGAATTAAAAGAGTGA	CATTGCTATTCAAGTACAAATGAAAAAAAA	
NM_006191	5036	ENSG00000170515	PA2G4	12	1202	GAAGAAAATGAAGCTGGGGACTGA	ATCAAAGATTCTAAGATTTGTGTCTTCTGC	Y
NM_032786	84872	ENSG00000135482	ZC3H10	12	807	ACGGCCATGCCACACTGA	GTTCTGGGTCCCTACCCTTCTTC	Y
NM_001130420	6601	ENSG00000139613	SMARCC2	12	2017	GTGCCACCTCCACAGTGA	GCCCTGTACTAGCCATCTGG	Y
NM_018181	55205	ENSG00000074657	ZNF532	18	2191	AGGATGAGCTCAGCCGAGAAATAG	CCTCTGGGCATTTTTTAAAATTTTCTTGTA	Y
NM_018337	55311	ENSG00000167685	ZNF444	19	872	CCCTGGCCCTTGGGTTAG	CACAGCTGTGCCCAGACTC	Y
NM_024303	79149	ENSG00000131848	ZSCAN5	19	445	AAAACACATCCAGAAGCTACTTCTCAGTGA	CTGATACTAAAGAAAATTGTTTATCTGAAA	Y
NM_198332	6773	ENSG00000170581	STAT2	12	1971	TTGATGCCTTCTGACTTCTAG	AAGGGAAGAAGAAATATGACAAGAGGAATG	Y
NM_024831	96764	ENSG00000137574	TGS1	8	923	CTAATTCGAAGACCAGCCTCTGAAACCTAA	TTAATGTTTAACACGCTCATATTATTAAAA	
NM_144690	147948	ENSG00000018869	ZNF582	19	764	GGGGAAAGCCCATTAGCCTAG	CAATAACAAGGGGAATTGGGTATGGCT	
NM_017661	NULL	NULL	SUHW4	15	1502	GACCTGGAAGACGAAAAAGAAAGAAGTTGA	TTAAGTCCTAATTTAAAATAATGTATCAGC	
NM_001159861	147949	ENSG00000198440	ZNF583	19	843	AGACCTGTAGGTTTCATCTCCTGA	AAATATCCAGTGAATGCTGATACAAGCCAC	Y
NM_013435	30062	ENSG00000134438	RAX	18	2149	CCGTGGCAGGCCCTCTAG	GCTCAAGGTCACAGGTGCC	Y
NM_022103	63934	ENSG00000198046	ZNF667	19	2018	CAGAATACACATTCTGAAGAAAAAGCCTAA	AATTTGGATTGTTGTTTTATCAATTGTGGT	
NM_001031623	26036	ENSG00000112200	ZNF451	6	2011	GCTATTAGAAGAAGTCTTGAGGAAATGTAA	ATGTGCTCCACAGAGGGGTAA	
NM_004282	9532	ENSG00000112208	BAG2	6	1204	CAACAAAATGCTGAAAGCAGATTCAATTAG	TTTGTATATCAATAGGACTAGTTACTTTGA	
NM_003146	6749	ENSG00000149136	SSRP1	11	602	GCGTCAGGATCCGATGAGTAG	AATGCTCGTCCAGCCGGG	Y
NM_021216	58491	ENSG00000197951	ZNF71	19	1599	CTGCGGATTCACACCTGA	ACAGCTATGGCTCCAATGATGAGA	Y
NM_001005850	90485	ENSG00000127903	BC37295_3	19	1113	GGACCAGCAGAAGATTAA	AAATCTTTTGTAGAGGAGAAGCACTCCCTT	Y
NM_033273	90827	ENSG00000185177	ZNF479	7	401	ACTGGAGAGAAACCCTACAAATGTGAATAA	GTGGTGGCACACGCCTGTAAT	Y
NM_001270523	5015	ENSG00000165588	OTX2	14	1208	TGGAAATTCCAGGTTTTGTGA	TCTATTTTATGCATAGATTAGCAAAAAAAA	
NM_005967	4665	ENSG00000166886	NAB2	12	935	GAGGCCAGCCGGCAGTGA	CCAGGGTGAGAGTCTGGGTC	Y
NM_001178080	6778	ENSG00000166888	STAT6	12	1348	GCCAACCCCAGTTGGTGA	CCCCAAGTCCTTTGCAATTTCTTC	Y
NM_001145460	84525	ENSG00000171476	HOPX	4	979	TCTCATTTCTCTCTTCCAGAAATGGTTTAA	CTGATGAGGCTGGTCCTGGAA	Y
NM_052882	114026	ENSG00000141946	ZIM3	19	1002	CAGAAAAGAATTCACTCCAGATAG	GGCTGAGTGCCATGGCTCAT	
NM_001252226	10769	ENSG00000145632	PLK2	5	780	CTGAACATGCTCTTACAAAGATGTAACTGA	GACTTCCTAGAAACTGTAGTTAGAAAAAAT	Y
NM_006635	10794	ENSG00000197714	ZNF460	19	2018	TTCATAGTGGAAGAAACCCTACCATTGTAA	GAGTCTGAGACCAGCCTGAATAACAC	Y
NM_213598	125919	ENSG00000178229	ZNF543	19	1691	AATATCACCACTGAAGAAAATCTGTGGTGA	AAAAAAAAAAAAAGGCTCTGAGGAGGGAAG	
NM_020657	57343	ENSG00000131845	ZNF304	19	2228	CCTTTAGCTGCATCTCTTAAACTTGTTTAA	TGAGTTGAAAACATCTCCACAGAAAAACTA	Y
NM_173631	284306	ENSG00000152433	ZNF547	19	1552	CTCAGACATCAGAAAGTCCACACTGGATAA	AGGTAAATTGCTGTATTTTGTGACCTGACT	Y
NM_004083	1649	ENSG00000175197	DDIT3	12	401	GTGAATCTGCACCAAGCATGA	AAACATTGTCCGAGAACTGAAAGCACAA	Y
NM_006959	7565	ENSG00000186272	ZNF17	19	638	CACCAGAAAGTTCACACCAGATAA	AGCCTACAGTATTCAGTACAGTAATATGCT	Y
NM_001023561	388567	ENSG00000186230	ZNF749	19	518	CAGATAATTCATACTGGAAAAAGGCCTTAG	TTAAGTTACCTGTAATTTGCCACAATTATA	Y
NM_001098494	79744	ENSG00000105136	ZNF419	19	770	CACACTGGAGAAATGCAGTGA	TGAAATGCTAACCATTTAGAACACAATTTG	Y
NM_198542	374928	ENSG00000152439	ZNF773	19	898	CGAAGGATTCACACTGGAGAAATACAATGA	AGAGGGCTATTTTCTCAGGAGGAACAAT	Y
NM_017879	55659	ENSG00000083817	ZNF416	19	733	CCCTACAGCCCAAGATCTAACATTGTTTAA	CTAAAACCAGAACAAGAACTACTGTCATTT	Y
NM_001010879	284307	ENSG00000171649	ZIK1	19	1295	ATACATCACCAAAAATGTCATAACACATAG	TCTTTATCCTCCTTTTTCTGCCTCTACA	Y
NM_020880	348327	ENSG00000183647	ZNF530	19	1124	CACAGAAGAGTTCATGTGCAGTGA	ATGGAGCAAAATCTATATTCTGTGAGCATA	Y
NM_003435	7693	ENSG00000213762	ZNF134	19	1045	ACTGCAGGCAGGCTTTAG	CCCAATCCCAATTACTCACACTCAATATTT	Y
NM_000075	1019	LRG_490	CDK4	12	978	CTACATAAGGATGAAGGTAATCCGGAGTGA	TTCCTCTGTCCATATACTGGATCACCT	Y
NM_006385	10520	ENSG00000121417	ZNF211	19	778	CAGAGAGTTCACATTGGAGAAAAGCCTTAG	GGCCAAAGCTGGAAAGACAGTTGTAT	Y
NM_152677	201516	ENSG00000180532	ZSCAN4	19	427	TCCACACCAGAAGCTTCCTAA	ACTCCAGTCTGAGCGACAGATCAA	Y

NM_138347	90233	ENSG00000204519	ZNF551	19	1693	GTTCACACTGAAGAAAGGCCTTAA	CTGAGTCACAAAAGCCACATCCCT	Y
NM_001085384	7710	ENSG00000179909	ZNF154	19	1373	ATTAAACATCAGAGAATTCATAGTCGATAA	CCTTGAGGTGAAAATCCCAGAGG	Y
NM_024833	79891	ENSG00000083814	ZNF671	19	910	GTTCATGCTGGAGAAAAGCTTTAA	TAGGTCAAAGCTGTAATCAGAGCTGTTCTC	Y
NM_001206897	8854	ENSG00000128918	ALDH1A2	15	1953	AAGATCCCCCAGAAGAACTCCTAA	AGTTGCCTTAATGAATTCTGTAAAAATACC	Y
NM_017652	54807	ENSG00000083828	ZNF586	19	1000	GTTCATACTGGAATGAGGCCTTATAAGTGA	AAACCACAAATCCCGTGGACC	Y
NM_024762	79818	ENSG00000178935	ZNF552	19	1138	CAGAGAGTTCACAAAAGAAAGGGCTTATGA	AAACACAAGCAGCAACTCAAATATCCATAA	Y
NM_025027	80095	ENSG00000166704	ZNF606	19	1408	AGAAATCACAGTGAAGAGAAACTGAATTGA	TTATCCTTGGGGTTGCAGGCA	Y
NM_016284	23019	ENSG00000125107	CNOT1	16	1192	GGGACAGGTGCCAGTTAG	AGTAGGAATGACCTATCAATCAATCAATCA	Y
NM_007134	7694	ENSG00000176293	ZNF135	19	1461	CACCAGAGAACTCACACTGGATAA	GACTATCTGCAACATGGTCTCCC	Y
NM_024620	79673	ENSG00000181894	ZNF329	19	1730	GAGCAACCCATGGAAACATAA	CTTACAGCTTCCCTGTTGCCCT	Y
NM_133502	10782	ENSG00000171606	ZNF274	19	598	AAGAAGAAACAGCCTACCTCATAG	ACTCACATAGTATCTTGTGCCCAAGG	Y
NM_014480	27300	ENSG00000198131	ZNF544	19	1068	ACACATACTGGAGAGAAACCTTAG	GAGACAGTTTTGCCATGTTTGCCA	Y
NM_021089	7554	ENSG00000278129	ZNF8	19	532	ATGTTATTTGACATCAGAGAATCCACATAG	AAGGTCACGTTCCACATGGGAA	Y
NM_173548	201514	ENSG00000171574	ZNF584	19	721	GGGAAGGTCGTTAGCTGCTAG	ACACCTGTAGTGCCAGCTACTCA	Y
NM_003433	7691	ENSG00000131849	ZNF132	19	689	CTTGCACAGCATAAAAAGATTCATACCTGA	ACACAGCAATGCAAAGTAGGCTACAA	Y
NM_207395	388569	ENSG00000249471	ZNF324B	19	1433	AAGCCAGCGAAGGTCTGA	CGGAGGTTGCGGTGAGCC	
NM_014347	25799	ENSG00000083812	ZNF324	19	1604	CAGCCAGCGGAGGTCTGA	CTCTGTGGCCTCTTGAGGGTA	Y
NM_017908	55663	ENSG00000083838	ZNF446	19	684	CGGCCGGAGGTTCCATGA	TGGGGACCTGGGACCTGG	Y
NM_001267033	7593	ENSG00000099326	MZF1	19	1423	AACGCAGCAACCTGCTGA	ATGCTAGGAGCCACCTCTCTCA	
NM_001013843	79811	ENSG00000137776	SLTM	15	1134	CCTCCGCGACGATTCTGA	CTACTGAGGTGAAGAGTCTAAGCTAGATTT	Y
NM_002228	3725	ENSG00000177606	JUN	1	1464	ACGCAGCAGTTGCAAACATTTTGA	TTCTAGTTTGACTCTTCCTAAATTCTTTGC	Y
NM_173557	220441	ENSG00000176641	RNF152	18	961	ACTGTGATATCCTGTGGCTGA	TCAAGATGTCCAAGAAGTGGCCA	Y
NM_005994	6909	ENSG00000121068	TBX2	17	1140	CGGGAGTCGCCCAAGTGA	CGCCCTGCCGCCGTGCCG	Y
NM_018488	9496	ENSG00000121075	TBX4	17	967	GAGAACTGGACTGACGGATGA	GCTCCAGTGTGGCCTCCT	Y
NM_004492	2958	ENSG00000140307	GTF2A2	15	1250	CTAGATACTGGCTCCAATACTACAGAATGA	CATTTCATCCATTAAAACCAGTTTTCATAT	Y
NM_003338	7321	ENSG00000072401	UBE2D1	10	2168	AGAGAATGGACTCAGAAATATGCAATGTAA	TTGTTAGAGTTAAGCAAAAATAATATACCC	
NM_003185	6874	ENSG00000280529	TAF4	20	1550	CTCTACAAAGCATTCCTTAAGTGA	CCTCACAGAGAAAGTTCTTTCTCCC	Y
NM_018014	53335	ENSG00000119866	BCL11A	2	1588	TCGAGAGCCCTTAAGTTCTGA	TCTCTTACTGATGTGGCCTCTGG	Y
NM_007374	4990	ENSG00000184302	SIX6	14	735	AGCGAGTGCGACATCTGA	GTTTAAAACATATACCAGGAAGCACCAAAG	Y
NM_080473	140628	ENSG00000130700	GATA5	20	1519	GCGCTGGCCTTGGCCTAG	AGGGGGAGATGGGAGACC	Y
NM_005982	6495	ENSG00000126778	SIX1	14	1764	GTGGACTTGGGGTCCTAA	GAACAGAAATAGGAAAGAGTAAGAAAGAAA	
NM_002908	5966	ENSG00000162924	REL	2	688	TCCTTTCCATATGAATTTTTTCAAGTATAA	TGGCCCATGGGCCTCAAAAATAAA	Y
NM_006602	10732	ENSG00000101190	TCFL5	20	1040	TCGATGGAGATCAAGTGA	AAGGTAGGGTGTGTATATATAAAAGGTTGT	
NM_022105	11083	ENSG00000101191	DIDO1	20	968	GGAGTTGCAGCTTTGTGTTAA	TGCTTGCCTGCATGAGTG	Y
NM_017780	55636	LRG_176	CHD7	8	2274	GAAATAGAAAACAATGAAAATGATGAATAA	CCCCAACAACTCAGGGGTC	
NM_017798	54915	ENSG00000149658	YTHDF1	20	1458	CGGCAGAGTCGAAACAAACAATGA	AACAGCTCCCTCTTCCAAAGC	Y
NM_001098426	6603	ENSG00000108604	SMARCD2	17	1037	GGAATTCGCCTGACCTAA	CAGTGAGAAAAACATGTCCATCAAGAAATT	Y
NM_006255	5583	ENSG00000027075	PRKCH	14	1329	GTGTCTCCAGAATTGCAACCATAG	TACTTCAGAGAATCACAGAAGAACTTACAT	Y
NM_006430	10575	ENSG00000115484	CCT4	2	745	TTTTTCTCTTATCAGGTAAACACTCGATAA	TAAAGGGAAGAGACTAGTTACAGATTTGAC	Y
NM_001433	2081	ENSG00000178607	ERN1	17	1138	ACTCCAGACGCCCTCTGA	CTTCAAGTTTAGCTTACTTATGCTGACATA	Y
NM_181054	3091	ENSG00000100644	HIF1A	14	1500	TTAGGTATCTCTTTTGTTTTTCAGATTTAG	TACATTAAGGTGATGGCACTAAGATAAATG	
NM_003082	6617	ENSG00000023608	SNAPC1	14	1625	TCCAAGAAGAGGAGAAAACACTGA	AGTGGGGCCAATAATGGATCTACA	Y
NM_018008	55079	ENSG00000153266	FEZF2	3	591	ACTAGGACAGTGCAGAGCTGA	CAGCAAGAACACCGGTTTGG	Y
NM_001195654	84619	ENSG00000197114	ZGPAT	20	403	CACAAGAAGATGACTGAGTTCTAG	ACCCTGGCCTCCGATCTG	Y
NM_152688	202559	ENSG00000112232	KHDRBS2	6	1183	GAACACCCCTATGGTAGATATTGA	GCGGGGAAACTCAAAGGTTGAAGA	Y
NM_004396	1655	ENSG00000108654	DDX5	17	1847	TATCCAATGCCAACAGGATATTCCCAATAA	TTCTTCTTTACAAGTGTATACAAGGGTTTA	Y
NM_024784	79842	ENSG00000185670	ZBTB3	11	1307	TTTGTATTGGCCCCAAAAACTAACATCTAA	CGGAGCTGGCAGTGAGCC	
NM_001786	983	ENSG00000170312	CDC2	10	1057	GATTTGGACAATCAGATTAAGAAGATGTAG	CCTGTTAACGAGGAGGAAAAATAGAGGAGA	Y
NM_018419	54345	ENSG00000203883	SOX18	20	620	GCGTGCATCTCCGGCTAG	GTCAGTGTGCAGAGGAAAGGC	Y
NM_004535	4661	ENSG00000196132	MYT1	20	1985	AGGGGCATCCAGGTCTAG	GCTAAGCTGACACCAGAGGGG	Y
NM_001199770	5013	ENSG00000115507	OTX1	2	1695	CGGTTCCAGGTCTTGTGA	TCTCTTGCCAAGTCCTCCC	
NM_017490	2011	ENSG00000072518	MARK2	11	1963	ATAGCCAACGAGCTGAAGCTTTAA	GCCCTGTGGGTAAGTGGG	
NM_173587	283248	ENSG00000167771	RCOR2	11	824	CCAGCACCCTCACTCTGA	GTGGGCAAGATGTGGCCTAG	Y
NM_001170905	728927	ENSG00000234444	ZNF736	7	672	CATACTAGAGAGAAGCTCCACAAGTGTTAA	CTATTCAGACAAGCAAATGCTGAGAAAATG	Y
NM_013280	23769	ENSG00000126500	FLRT1	11	1061	GACATAGACTACTCCTACACATGA	AGCTGCGACGCCTCCCTA	Y
NM_178558	340252	ENSG00000173041	ZNF680	7	1464	AAATGTGACAATAATTTTGATAACACCTAA	ACAGCATATTAGTTGAAATAAGCCAGGCAC	Y
NM_004451	2101	ENSG00000173153	ESRRA	11	952	GAGGCCATGATGGACTGA	GCTGAGTGAAGAGGAAACTGAGAGTTG	Y
NM_014326	23604	ENSG00000035664	DAPK2	15	1664	AGGAGCAGCACCTCCTAA	TCATCCGTCTTTTTCCTCTCCCTTCT	Y
NM_001160183	7697	ENSG00000197008	ZNF138	7	2341	TTGCCCAAGACCTTTGGCTAG	AACCAAGATTGCACCAGTGCACT	
NM_006524	7697	ENSG00000197008	ZNF138	7	1625	GAATGTGGCAAAGCTTTTAACCTATCTTAA	AACCAAGATTGCACCAGTGCACT	
NM_004630	7536	LRG_617	SF1	11	1394	CCTCCACCACAGAACTAG	TAATTAACCACCCACAGTCTCACC	Y

NM_004579	5871	ENSG00000168067	MAP4K2	11	580	CACCAGAGCACCTACTAA	CTGGGGGCCATGGCCTCC	Y
NM_130800	4221	LRG_509	MEN1	11	1000	CAGCGCAAAGGCCTCTGA	GGCGGAGCCTACGTCCTT	Y
NM_001136178	1959	LRG_239	EGR2	10	1391	CGGACCCGGACACCTTGA	CAGGCTAGCAAAGAAGATCTGGAGA	Y
NM_017525	55561	ENSG00000171219	CDC42BPG	11	463	GAATTGGAGAGCTCTCCTTGA	AGAGACGGGGACATGCCC	Y
NM_001083592	4919	ENSG00000185483	ROR1	1	891	ATCCCAGCGTGCGGTAAATAG	GCTTTTCCAGCAGTGACATCCTGA	Y
NM_001214902	2100	ENSG00000140009	ESR2	14	1037	AAGCAATTCATTCATTTGAAGTTATCTTAG	GGTTTCGCCATGTTGGCCA	
NM_016213	9325	ENSG00000103671	TRIP4	15	419	GGGTTAATGAAGCAGAATAAAGCTGTCTGA	AAACTTTAAAATCTGAGAGGAACCCTCAAA	Y
NM_007139	168374	ENSG00000146757	ZNF92	7	1390	AATTATACTAAAGAGAAACTACAAACCTGA	AAATAATATACTGTTACCATCTTTTACCCC	Y
NM_013254	29110	ENSG00000183735	TBK1	12	918	CGCAACGTTGACTGTCTTTAG	AACTTCATACTAAATTTAGAATTAGTGGGT	Y
NM_014950	22890	ENSG00000126804	ZBTB1	14	1973	TCTAAACCTGTGGAGAAGTGA	ACCTGAGAACATCATAATTAGCTATATCAG	
NM_194272	348093	ENSG00000166831	RBPMS2	15	1464	TGGAAGTACCGTCAGTTCTGTTAG	CCTCCCCCACCACCTTTACA	Y
NM_006268	5977	ENSG00000133884	DPF2	11	1401	TACCAGAACCAGAACTCCTCTTGA	CATATTGGGGTTCCACAAAAAAATTTTAAT	Y
NM_018211	55225	ENSG00000162437	RAVER2	1	2426	ACTTACTTAAAAAAGAAGCGAGTATACTGA	GAGCCCAAATTCAGGATTGGTTCAG	
NM_002227	3716	ENSG00000162434	JAK1	1	1519	ATTGAAGGATTTGAAGCACTTTTAAAATAA	AAGAAGTGCAAATCAAGTGGTGTTTGTTTG	Y
NM_001145138	5970	ENSG00000173039	RELA	11	962	CTGAGTCAGATCAGCTCCTAA	ACTGGACGGTAACTCGGGGTT	Y
NM_182710	10524	ENSG00000172977	HTATIP	11	605	AAGAGGGGGAAGTGGTGA	CGATCCCTTCACAGCAGTAAACCA	Y
NM_152414	27319	ENSG00000180828	BHLHB5	8	1878	CAGTGCACGGAGAAGCCTTAA	TAAACAAGTAAATTAGGGCTACTTCTAATT	
NM_145112	4149	LRG_530	MAX	14	1532	CGGATGGAGGCCAGCTAA	GCAACTCTGGCTTATGGCTGCT	
NM_001167614	23592	ENSG00000174106	LEMD3	12	2216	ACCAATTCTCAAGGAAGTTCCTGA	AGGAAAAATAAAATGCTGAGGGGAAGGATA	
NM_005438	8061	ENSG00000175592	FOSL1	11	845	ACCCTCCTCGCTTTGTGA	GCGGATGTAGCCCCACTTGT	Y
NM_004459	2186	ENSG00000171634	BPTF	17	2270	AACAACAAACTGCAGTCTACAGCTTCTTAA	CTTTATACCGTTAAATACACATATGAAATT	
NM_178864	266743	ENSG00000174576	NPAS4	11	897	GGGGAACCAACGTTTTGA	GAATGAATGAATGAATTCACAAGTGGATGA	Y
NM_004480	2530	ENSG00000033170	FUT8	14	1891	ACATATCCTGAGGCTGAGAAATAA	CAATTTAGAAGGAAAAGAGACTAACTTAAT	
NM_003484	8091	ENSG00000149948	HMGA2	12	577	AACAGTACCAAAAGGAGTCACTGA	CTGGCAGAAGTCATGGCAGC	Y
NM_021150	23426	ENSG00000155974	GRIP1	12	1768	GAGACACGAGAACCCACTAATACATTATAG	GGCAGGGGTTGCAGTGAG	
NM_002755	5604	LRG_725	MAP2K1	15	1109	CATGCTGCTGGCGTCTAA	CCCATTAGTCCAAAGCACTGAACTC	Y
NM_001619	156	ENSG00000173020	ADRBK1	11	1288	AGTGCCAACGGCCTCTGA	CACCTGGAAGATGGCCTGG	
NM_001142861	NULL	NULL	SMAD6	15	644	ATCCTCCTCAACAACCCCAGATAG	AGAGTAAATGTGACTTTCCTCCCTTTTCAA	Y
NM_022845	865	ENSG00000067955	CBFB	16	2457	GGTGATGACCTCAAACTTCGTTAA	CATAATATACACTGGGGGATTTGTATACCT	Y
NM_001950	1874	ENSG00000205250	E2F4	16	975	GTGCCTGTTCTCAACCTCTGA	CCCGCCCCACACCAAGGT	Y
NM_005995	347853	ENSG00000167800	TBX10	11	482	GGCCAGGACTCCCAGTGA	CTTTTAATATTACTGTGTGCTCCCAGGGAT	Y
NM_001077702	57708	ENSG00000198160	MIER1	1	2326	AGTTCAAGAGCCAATGCCTTTTTAAAATAA	GCTTGTCATCATTGACATTTAAATGTGAAA	
NM_002758	5608	ENSG00000108984	MAP2K6	17	756	TCTTTTGTAAAACTGATTCTTGGAGACTAA	TTTTTATGTTTTCCAAAGTTGGGAAAAAGA	Y
NM_006565	10664	ENSG00000102974	CTCF	16	1493	AGCATGATGGACCGGTGA	CCCGCCAAGATCATATCGTCC	Y
NM_018448	55832	ENSG00000111530	CAND1	12	1958	ACTAACTTGGAATCAATGGACACTAGTTAG	AAAAACAAAGCAAAACAAAACAAAACATGC	Y
NM_020457	57215	ENSG00000168286	THAP11	16	876	AAGAAGCACGGAATGTGA	GTGCTTAGGGCCCATTTTTGGAAAAA	Y
NM_198443	123904	ENSG00000188038	NRN1L	16	289	CTGAGGCCTCTGGCCTAG	TGGGAAATGAGTGTTTGTAAGGAGGAAGAT	Y
NM_017635	51111	ENSG00000110066	SUV420H1	11	1828	GAAGATCAGTCTTTAAGGCTTAATGCCTAA	CAAGGATGAGACTATGTAAGACTGCAGAAG	Y
NM_006742	5681	ENSG00000159792	PSKH1	16	2216	CAGCAGCAATACAATGGCTGA	GGGTCCGACTCCCTACTTGGA	Y
NM_024939	80004	ENSG00000103067	RBM35B	16	1859	AAGGAATGGGTGTGTTTGTAG	CCCCTGGTGTAGCTGCCT	Y
NM_020143	56902	ENSG00000115946	PNO1	2	1340	AGCAGATCAGCAGATCGATTCTGA	AAGCATTCTTTCATCATTTCACCTTTACTA	Y
NM_016166	8554	ENSG00000033800	PIAS1	15	411	ATCATACCAGACATTATTTCATTGGACTGA	TGTTATTGCAAATAAAATAATGCCATCGAA	Y
NM_022909	64946	ENSG00000153044	CENPH	5	738	CAGCTTGAGAAGAATGTTGACATGATGTAA	ATGTTCAACAGCTCCAAATTAATAAAGTGT	Y
NM_016283	NULL	NULL	TAF9	5	515	GAGCAGTGGATCAAAGATCATAACTCTTGA	TAAAGGGAAGGAACCTGGGGT	Y
NM_173545	NULL	NULL	C2orf13	2	2320	GAAGCAAAAAGGTTTATGAAAAGAAAATAG	GTATAGAACAGCAATTAATTTAAAAAATCA	
NM_001244698	677	ENSG00000185650	ZFP36L1	14	2058	TTCAGCAGACTTTCCATCTCAGATGACTAA	GCTATAGGCTTAGCTTATAGGGATGAGAGA	Y
NM_016101	51388	ENSG00000132603	NIP7	16	1638	CATGAAGAGACGTTGACTTAA	ACAGTGAAAAAGTTCTGAAGAAGTATGTAT	Y
NM_005652	7014	ENSG00000132604	TERF2	16	1517	ATGAAAAGACTTGGCATGAACTGA	AACTTCCATATCATCTGTTTGACATAGCTT	Y
NM_001142498	23411	ENSG00000096717	SIRT1	10	1977	GACATGAACTATCCATCAAACAAATCATAG	ACCATCAGGGTTTTGCAAGTACTG	
NM_173215	10725	ENSG00000102908	NFAT5	16	497	AACAACTTGACTGGCTCCTTTTAA	CTTTTGACTATGACCCTATCATATGAGGTT	Y
NM_006530	8089	ENSG00000127337	YEATS4	12	660	GAAGAAGATGACCAAGCAAAAGACATATAA	AGATATAATCTTTACCATCAAAGAGCTTCT	Y
NM_001184968	4286	LRG_776	MITF	3	879	GCCTTTCAGTTTATGAAGCAGTGA	GTGTTCATCCTCAGGCTGCCAA	Y
NM_145178	220202	ENSG00000179774	ATOH7	10	804	TTCCAGATGGCCACCTAG	TCTCATCAGCTTTTTGAGGAACTACTACAA	Y
NM_012207	3189	ENSG00000096746	HNRPH3	10	1322	TGGCGTGGGATGTACTGA	AGATGAATCATAGCTTCAGTCAAGAGGCTC	Y
NM_000346	6662	ENSG00000125398	SOX9	17	2212	ACACAGCTCACTCGACCTTGA	GTCTTTTCCTCAGCTTGTTTTCTAGGATTG	Y
NM_006196	5093	ENSG00000169564	PCBP1	2	568	GGCATGGGGTGCAGCTAG	TTCCTTTGTTGGGGCACAGAG	Y
NM_005938	4303	ENSG00000184481	MLLT7	X	1630	TTTCTTCTTCCCACAGATCCCTGA	CTCCACCCCCACACTCCA	Y
NM_001105192	7090	ENSG00000140332	TLE3	15	2073	AAGGCCACAGTTTATGAGGTCATCTACTAA	TGTGAGATGAGAAGAGCTCACATCGAT	Y
NM_201599	9203	ENSG00000147130	ZMYM3	X	1472	GAGGAAGACCTGGACTGA	AAAAAAACAAAAAAAACACCACATTCCCAG	Y
NM_003096	6637	ENSG00000143977	SNRPG	2	440	ATCATGTTAGAAGCCTTGGAACGAGTATAA	TGTAGATGTACTTGGGAAAGTCAGGCTA	Y
NM_001145410	4841	ENSG00000147140	NONO	X	1272	AACAAACGTCGCCGATACTAA	TTGATGCACCTCATCCCCAC	Y

NM_018237	55749	ENSG00000060339	CCAR1	10	466	GAGAATGGTGCCAGTGTATGA	AACTTGCAAAAATTAAGCATCATGCTATTT	Y
NM_138923	6872	ENSG00000147133	TAF1	X	2139	AGTGACTTGGACTCTGATGAATGA	TCCTTCTCTACCCACAAATGCCAA	Y
NM_024504	63978	ENSG00000147596	PRDM14	8	606	TCCCACATGAAGTTTCATGAAGACTACTAG	AAGTAGCTGGTATTACAGGCATGTG	Y
NM_006540	10499	ENSG00000140396	NCOA2	8	1780	TTATCTGTTTTCCTACAGAAATATTGCTGA	ATCAAAAAGATATCATTCTGAAATTTAGGT	Y
NM_005466	10001	ENSG00000133997	MED6	14	753	GAAAAACGGATGAGACTTCAGTGA	AAATGTCAGCAACTGTTTATTTTAATACTG	Y
NM_012476	25806	ENSG00000116035	VAX2	2	421	AGCTGCAAGAAAGCTAACACTTAA	TGACCGACACATGTGAGTCC	Y
NM_020147	56906	ENSG00000129028	THAP10	15	1292	GCTGTACAGGTGAAAGAAGAAACATGTTAA	TACATTCCAGCCTGGGCGATAGA	Y
NM_001012505	27086	ENSG00000114861	FOXP1	3	201	CCTAACCCTTCCCCATTTTGA	CTCCGCCTGTCTTTCTATGTATTTATTTCA	
NM_020999	50674	ENSG00000122859	NEUROG3	10	544	CTGGCTTTCTCAGATTTTCTGTGA	TGAGGCGCCCTGAAATCC	Y
NM_145911	7571	ENSG00000167377	ZNF23	16	673	CAGAGTGTCCATAGTGAAGGAAAATCCTAA	TTTTAAACTTTAAAACAAAAACATACAGGA	Y
NM_006961	7567	ENSG00000157429	ZNF19	16	1277	CCAGAATTTTTTACCCCCTTTTACTGGTAA	AAGGTGTTTACTCAATTCCCCAAGTCTATC	Y
NM_203350	9406	ENSG00000132485	ZRANB2	1	1963	GGTTCCCGTTCAAGTTCAAAAAAGAAATAA	AATTAAAAATTCTGAGGTTGCAGTGATAAT	
NM_002732	5568	ENSG00000165059	PRKACG	9	684	AAGTGTGCCAAGGAGTTTTCTGAGTTTTAG	CCCCAGAGCCTCCTTCCAAA	Y
NM_001252613	27332	ENSG00000075292	ZNF638	2	434	GCTGAAGAAAGAAGCTCTAGGTGA	TGGTTGAAGCAAAGTAAAACACTGTCAT	Y
NM_001166448	55869	ENSG00000147099	HDAC8	X	1460	CCAAATCCAGGCCTGTAG	CTCGTATTTTCTTGCAAGTATGCCTTTTGT	Y
NM_152625	167465	ENSG00000178175	ZNF366	5	529	GAAAAACAAGCAGTGCTTTTAGGTATCTAA	CTGTGCCATTTCTGACATTTTTGGTTATAG	Y
NM_001201556	55565	ENSG00000102984	ZNF821	16	1170	TAACCATCCAAAATGTTTCCTTTCCAGTGA	ATAAGGACCTCTGCTTGGACAG	Y
NM_005169	401	ENSG00000165462	PHOX2A	11	852	CTGAAGACCAATCTCTTCTAG	GTTTGCTTAGTTCTCACTGTCTCCTG	Y
NM_014249	10002	ENSG00000278570	NR2E3	15	752	AAGCTCCTTTGTGATATGTTCAAAAACTAG	CTGGGTGGTTGAATTCTATGGGAGATTTTC	Y
NM_172058	2138	ENSG00000104313	EYA1	8	2087	TTGGAACTGGAGTACCTGTAA	TGCAAGTTCAGTCCCCTAAAGGAC	Y
NM_001042506	645974	ENSG00000184388	LOC645974	X	1647	GAGGCCACCTTGCGATGA	TGGCTTGCATAAGAAAACAAAATGAAACAA	
NM_001012977	340529	ENSG00000186288	LOC340529	X	1626	GAGGCCACCTTGCGATGA	GAGATATAGACATAGATACATATGTACACA	
NM_001146189	55628	ENSG00000215421	ZNF407	18	525	GGTAAGGGACAGAAACTGTGA	CCCAACCTAGGGCCAGCC	Y
NM_000281	5092	ENSG00000166228	PCBD1	10	620	CAAGTAGCAGTGTCCATGACATAG	AATGAATCTGAAGGCAAGGTGGTCT	Y
NM_004472	2297	ENSG00000251493	FOXD1	5	887	AACTTTACTGCTAGGATTTCCAATTGTTAA	TTCAAGTGAAAAATCATGGCATGATGCT	Y
NM_005098	9242	ENSG00000178860	MSC	8	1299	CTATGTGGAACCACCGCTTAA	TAAAGAATGACGTTTGGAAATGGAGTGATC	Y
NM_017757	55628	ENSG00000215421	ZNF407	18	1384	GAACTCCAGGAAGCATGA	AGAAACAGTATCGGGCTGCAG	Y
NM_001207	689	ENSG00000145741	BTF3	5	565	TCCAAGAATGAGGCAAACTGA	GAATATCAGGGAGAAATGTCTGTTTATCCA	Y
NM_032408	9031	ENSG00000009954	BAZ1B	7	1978	GGACGAAGACAGAAGAAGTAG	GGAGTACTTCTTTCTGTGGTTAAGAACACT	Y
NM_005786	10194	ENSG00000179981	TSHZ1	18	1489	ATCTATGTGACTGAGTTGGAGAAACAGTAG	AAACGCAATACTTATCATTTGAATGAAACA	
NM_032953	51085	ENSG00000009950	MLXIPL	7	851	ACCCTTGGCAAACCTTTATAG	ACCACTACATCTGATCTTGTCCCAC	Y
NM_004097	2016	ENSG00000135638	EMX1	2	1117	ATCGATGTCACCTCCAATGACTAG	GGTCTGGATGTCGTAAGGGAAAGACTTA	Y
NM_006937	6613	ENSG00000188612	SUMO2	17	789	ACGGGAGGTGTCTACTGA	CTGTTATTCTTTGTTTGGCATCAGTATCAG	
NM_178441	53349	ENSG00000165861	ZFYVE1	14	1611	AATAAAAAGCCCGGTGACCTTTAA	GTAAAAGACTGCAGTATTTGCAGGTAAAAT	Y
NM_001134462	344022	ENSG00000214513	NOTO	2	463	TCAGGAGTGGACGGCTGA	GTGTTGATTGTTGTGGGGTGACA	Y
NM_001965	1961	ENSG00000135625	EGR4	2	708	TCCTTCGCTTCTCTCTGA	CGCAGTTCCTGGCAGGTGT	Y
NM_001204426	3984	ENSG00000106683	LIMK1	7	1404	CCTGAGGTCCCCGACTGA	TTAAGGGATCGTCCTGTCTCAGC	
NM_021239	58517	ENSG00000119707	RBM25	14	1743	GAAGCCAAGAAAATTGGTCTTGTGAAGTAA	AAATAAAAGAGACATTTCCATAGATGTATA	
NM_022170	7458	ENSG00000106682	EIF4H	7	1971	GTCGTTCAAAAGGAGCAAGAATGA	TTTCTAATCATCACCAGCTTTTAAAATAAC	
NM_001730	688	ENSG00000102554	KLF5	13	1832	ATGAAGAGGCACCAGAACTGA	CAGAGCGAGACTCCGTCTCAAAAAATAAAT	Y
NM_001080419	85451	ENSG00000132478	UNK	17	1568	CACACCCTCCAGTCGTGA	GGAAGTTTCCAGCCCTCTCCA	
NM_001198798	51008	ENSG00000138303	ASCC1	10	1482	TGTGGACAAATTGACTTCTCCTGA	AAAAAAAAAAATCAACCTTGCAGGTACTAA	Y
NM_198889	26057	ENSG00000132466	ANKRD17	4	1626	CATATGAACCAGCTTGGCTGA	CAGATGGAAGAAATCATTTGATTTACTTGA	Y
NM_017489	7013	ENSG00000147601	TERF1	8	1795	ATTTCCTCAGACAGCGAAGACTGA	TAGATTTGATGATCTATAGCAGGTGTTTGG	
NM_001258	NULL	NULL	CDK3	17	756	CAGCGATTCCGCCATTGA	AAAAAATCATGGACTAGCCCCTGTCT	
NM_001025290	340168	ENSG00000203909	DPPA5	6	438	CTAGGCCCTTGGATGAAGTGA	CTCCACCCTTTTTGACACTAGCCAA	Y
NM_018665	55510	ENSG00000080007	DDX43	6	1520	GGAAGGCCCAAGAAGTTTCATTAA	GGTAAACTGTTGTTGCATATTTTGACCTTA	Y
NM_001454	2302	ENSG00000129654	FOXJ1	17	1199	GTGGGGGCCTTCTTGTAA	GTCATGGGGAGTTGGTTAGTGCT	
NM_001163636	2969	ENSG00000263001	GTF2I	7	2034	CCAGACCCCACGTGGTAG	TCTGAGAAACACCGCAAGTGG	Y
NM_172037	157506	ENSG00000121039	RDH10	8	2429	AACAATAATGAAGCAAAAAATGGAATCTAA	AGGAATTCCATAGTTCTAAACCTGCATTAA	
NM_033244	5371	ENSG00000140464	PML	15	2079	AGGAACGCGTTGTGGTGA	ACCTCTGATGCCCATGAGTGT	Y
NM_001242928	57862	ENSG00000119725	ZNF410	14	959	GATTTGCTTTCAATCTTTTACAGTTACTAA	GCTTAAGAAATGAAGATGTTCTTGTTGTTT	Y
NM_001142620	64220	ENSG00000137868	STRA6	15	1394	AGACTAGTCAGAGCCTGGATTTGA	CCCGGAGCATTAGGAACAGTTCT	Y
NM_001003795	389524	ENSG00000174428	GTF2IRD2B	7	700	GTACTCCACATCGCAACGTGA	ACCAAAAAGCAAATATACACGTCTCTCTCC	Y
NM_001142497	197259	ENSG00000168404	MLKL	16	829	TTAAAGAAACTCTCCACCTTTTCTAAGTAG	TGGAAAGTCAGGTTGGAAAGTATGGTAT	Y
NM_001009812	85474	ENSG00000179528	LBX2	2	590	GAGATACAGGTGGACGATTGA	TGTAATTATTACCTACCTTAAAGGGTTGCT	Y
NM_182894	338917	ENSG00000119614	CHX10	14	1999	CTGGAGGACATGGCTTAG	CAAGTGATCTGCCCACCTAGG	
NM_001195427	6427	LRG_640	SRSF2	17	2226	GAAGGAGCGGTGTCCTCTTAA	AGCATTGCTTTTAGGTGTTCAACTG	
NM_006465	10620	ENSG00000179361	ARID3B	15	2537	ACCAGCTGGTCCCTCTGA	TTGAAGAAGGCGTGGGTGAAG	Y
NM_001130028	1198	ENSG00000179335	CLK3	15	498	CGCAACCCAAGCAGATGA	TCTGGGGCTGAGAGGTACAGAA	Y
NM_001127190	1445	ENSG00000103653	CSK	15	865	CACGAGCTGCACCTGTGA	CCCGGAGGGCAGACACAA	Y

NM_001005	6188	ENSG00000149273	RPS3	11	2228	CCAGTCCCCACAGCATAA	AGATTGATGCACAATGAGAATATAGAGCAG	Y
NM_001099436	25989	ENSG00000140474	ULK3	15	1290	GCTTGCACCCTTCAGTGA	ATGTGGCTATCATTCTCAGCATTCC	Y
NM_001156	310	ENSG00000138279	ANXA7	10	844	CTGGCTATTGTGGGCCAGTAG	CTGAACACTTAAAATTTGCTAAGGTAGTAG	
NM_153688	162239	ENSG00000184517	ZFP1	16	2080	ATCGGGGAGAAACCCTGA	CTTCTCTCATTCTCCAAGTCATCCTTTCAT	Y
NM_001080408	283571	ENSG00000119608	FLJ36749	14	2279	ATATTCAAATCTTCCAGCTATCCCCAGTAG	ATATACCTTAGTGGAAAAAATGACCAGATC	Y
NM_000689	216	ENSG00000165092	ALDH1A1	9	726	ACAGTGAAAATCTCTCAGAAGAACTCATAA	TTGGAAAATACAGCATGTTTCTTTACCTAT	Y
NM_001256114	431707	ENSG00000162624	LHX8	1	818	ATGACACAACTGCCAATAAGTCATACCTAA	TGTTTTTTTTTTTTGAATAATATGTTTCCT	
NM_005252	2353	ENSG00000170345	FOS	14	990	ACGCTGCTGGCCCTGTGA	TAAAATCAGCTCTATAGTTTCCTTGTCCTC	Y
NM_004705	5612	ENSG00000137492	PRKRIR	11	1084	GATAATTCCGAAACTGTGGAAAATACCTAA	TAACACTTTCTTAATTGTTTTTTTTCTGTA	Y
NM_032367	84327	ENSG00000132846	ZBED3	5	647	ATCACAAAGGTCCTCCTGTAG	AAAAAAGCGGGCTCTGGGACT	
NM_171999	27164	ENSG00000256463	SALL3	18	1827	GAGGATAACAAGGAGATTGGTATCAACTAG	TCCTCTTACAATGATTGTTGATGCTGCT	Y
NM_032109	23440	ENSG00000171540	OTP	5	1732	GAGCACACAGTCTCTATGAGCTTCACTTAA	CCCAGAAACCGAACCCAAGC	
NM_004452	2103	ENSG00000119715	ESRRB	14	1296	CATCTCTGGCTCACCATGTAA	TTTTAAAATGACACCATGGGACTTCCCA	Y
NM_001128620	5058	ENSG00000149269	PAK1	11	1464	TCCACTGATTGCTGCAGCTAA	AGAATACACATTCAGGTATATAGTGGTAAA	Y
NM_001321	1466	ENSG00000175183	CSRP2	12	416	CTTGTTCATGCCCAGTAA	AGATCCTTTTCAACAGCTGTAATGCAAACA	Y
NM_006162	4772	ENSG00000131196	NFATC1	18	1973	ACTTTCTCAATTTTTCTTTTCTTACAGTAA	CCCTTCCAACGGCGAATG	Y
NM_006914	6096	ENSG00000198963	RORB	9	1763	GCCACCGGCTGCAAATGA	GGAAACTTTACAAAGCAGGGGTTTGTATGT	Y
NM_016578	51773	ENSG00000048649	RSF1	11	868	GATTATGTCTGTAACAGTGAACAGTTATAA	AAGAAAGACCACATTCATTTCACTAATAAC	Y
NM_015975	51616	ENSG00000187325	TAF9B	X	2042	GAAGATGATGATGACAATGATATTATGTAA	TGCTTCACTATGATGGTGTCACATG	
NM_020649	57332	ENSG00000141570	CBX8	17	438	GACCAAGGCTTTTTTAAAGAGAAAAGATGA	CAGGCCAAGCCCCTCCAG	Y
NM_003655	8535	ENSG00000141582	CBX4	17	983	TTCAAGGAGTACGTGACGGTGTAG	CGCCTCTCGACTTCCCAG	Y
NM_014913	22850	ENSG00000101544	ZNF508	18	1716	CATAGATTGAACTTTGAATATGAACCATAA	TAATTAGACCTTTTGGGTTATGTTCTTATT	
NM_015534	26009	ENSG00000036549	ZZZ3	1	1315	AACTACTTTCCAGCAAACAGATGA	ATAATACATTCCTTATTCTTCATAAATGAC	
NM_012245	22938	ENSG00000100603	SNW1	14	667	GGCAAGAAGAGGAGGAAGGAATAG	TTTGCAGACATTGCCTTTCCTCC	Y
NM_001142545	57143	ENSG00000063761	ADCK1	14	743	TTCCCTGCTCCACTCTGA	GGCCTTCTGTTCCCTAGATCTCC	Y
NM_003902	8880	ENSG00000162613	FUBP1	1	1040	TTATGTATCTCTTCTGCACAGGGCCAATAA	AAAACAAAGCTTATCTATACTGCATAAAGA	Y
NM_004378	1381	ENSG00000166426	CRABP1	15	433	ACCAGAATTTATGTCCGAGAGTGA	CCAGCTTGCAACTGCTGACGTA	Y
NM_001080395	9625	ENSG00000181409	AATK	17	1286	GGTGAGAGTAAAGAGGCTTGA	CCTCCACCGGGGTGTGCC	Y
NM_006237	5457	ENSG00000152192	POU4F1	13	2500	ATGAAATTCTCTGCCACTTACTGA	AATATAGAATTCATTTTATTTAGAGCACCA	
NM_001265603	10933	ENSG00000185787	MORF4L1	15	852	TACCATCGGAAAGCTGTGTGA	ATGGATTAGCGCTACGTACCAAAGCTA	Y
NM_001109879	50945	ENSG00000122145	TBX22	X	838	TGGTATCCAGCAATTAACCATTACCTTTAG	ATTCAAATGTATTCTCCCTCTTGTAATCAT	Y
NM_032567	84654	ENSG00000164299	SPZ1	5	513	AAAATGAGGTCAGCTAGCAGCCTAAGATAG	GCTCAGGTTTGTCTCAAACTCCTG	Y
NM_005360	4094	ENSG00000178573	MAF	16	792	CGTGTACTTACCAGTGTGTTCACAAAATGA	GTCAGATACATTGTAAAAAATTATTACATG	
NM_002069	2770	ENSG00000127955	GNAI1	7	2096	AATAATCTAAAAGATTGTGGTCTCTTTTAA	CCAAATGTGGCATCACATCTCATAGCT	
NM_022118	64062	ENSG00000139746	RBM26	13	836	GACAATGAATCTCGTTCTTGGAGAAGATGA	AATAAGTTAGTGATTATAGCTCCTTTTGTG	Y
NM_001271007	201254	ENSG00000169689	STRA13	17	682	CAGCTCCTGGACTTCTAG	CCTGGGGGTGGGGCCGAG	Y
NM_002583	5074	ENSG00000177425	PAWR	12	822	GGTCAGCTGACCAGGTAG	CCAGGCCCGAAGAATAAAAAACTTTTAAAA	Y
NM_002439	4437	ENSG00000113318	MSH3	5	1133	GAAGAAACACAGACTTCTCTTCTTCATTAA	GTTAATGAACAGAAAGGTCCCAAATGAACA	
NM_002072	2776	ENSG00000156052	GNAQ	9	1228	TTGAACCTGAAGGAGTACAATCTGGTCTAA	AACAATGTCATCTTAAGGACAAAGAAAAGA	Y
NM_001040708	23462	ENSG00000164683	HEY1	8	1358	ACGGAGATCGGAGCTTTTTAA	CAACGGCACGAATTCACACAGTTTAGA	Y
NM_003318	7272	ENSG00000112742	TTK	6	505	ACTTTTGAAAAAAAAAGGGGAAAAAAATGA	TTTGGAATTCAAACCCTTCAGGATTTGTTT	Y
NM_001100625	55839	ENSG00000166451	CENPN	16	590	CTGTTTGTCCCATTGTATCCAAGATGTTAA	AGGTGGCTGGCTTAGTACATGAAT	
NM_002469	4618	ENSG00000111046	MYF6	12	697	GAGGAAGTGGTGGAGAAGTAA	AGGAAAGCCAAATTTCCTTTCAATTGG	Y
NM_005593	4617	ENSG00000111049	MYF5	12	797	AGTTCCAGGCTTATCTATCATGTGCTATGA	CCTAAACTGGGTACATGAGAATGGTAAATA	Y
NM_001099403	56978	ENSG00000152784	PRDM8	4	976	CACATGACCTCGCATAATTGA	TAGAGTACATAAATGCAAATGGTGCCAG	Y
NM_017429	53630	ENSG00000135697	BCMO1	16	745	GGGGCTCCTCTGACCTGA	CTCTTGCAAAGAGGAGTCCCACA	Y
NM_006259	5593	ENSG00000138669	PRKG2	4	1205	TCAGGCTGGGATAAAGACTTCTGA	TCCTACTTATTATTTGGGAGAGTGCAAGGA	Y
NM_032246	84206	ENSG00000183496	RKHD3	15	1562	GCCATCCGCATCTTTTCTTAA	AACCAGACACAACCCTGGC	
NM_007005	7091	ENSG00000106829	TLE4	9	1777	AAGGCCACAGTTTATGAAGTTATTTATTAA	CCACATTACTTTAACAACAAAAAAAACCAC	Y
NM_022550	7518	ENSG00000152422	XRCC4	5	698	AGCCCAGAAGACCTCTTTGATGAGATTTAA	ATAAATCTTTTATCAATAATCCCTGAAAAA	Y
NM_000307	5456	ENSG00000196767	POU3F4	X	537	GACACATCTTGCCATGATCTCTGA	CGACCAAACAAACCTTACTCTGAGTAAACA	Y
NM_031369	3184	ENSG00000138668	HNRPD	4	2200	GGTCATCAAAATAGCTACAAACCATACTAA	TATCACTTAGACTCTTCAGTTTCACTTTCT	
NM_024672	79725	ENSG00000168152	THAP9	4	1387	CTAAGTAACGATGGATATCCATTCAAATGA	CAGACATTTGTATTTTAGTCAAATGTAACA	Y
NM_001717	646	ENSG00000169594	BNC1	15	1719	TCTCCAAGTCACCTCCAGTAA	TGTCCACCAAACTCTCCCCC	Y
NM_005077	7088	ENSG00000196781	TLE1	9	695	AAGGCTACAGTCTATGAAGTCATCTACTGA	ATGCAATGGGAGCCTACCAC	
NM_001243158	9013	ENSG00000103168	TAF1C	16	1274	CCTCGAATGGGCTTCTGA	GGGAGAAAGCACAGGTCACC	Y
NM_021998	7552	ENSG00000147180	ZNF711	X	1714	CACAAAGAGGCTCTTATGTAA	TCATCGTAACTGTTTAAATTCCTAGTTCAT	
NM_017894	54993	ENSG00000176371	ZSCAN2	15	490	GAGGTGACCAGTTTGTAA	CCACTGACTAACCAGGCCTGG	Y
NM_001080508	9096	ENSG00000112837	TBX18	6	2426	TCATCTCAAGTATCTGCACATATGGTCTGA	TTACATTTAACACATGAAACATGAACAGCA	
NM_031283	83439	ENSG00000152284	TCF7L1	2	1106	ACCAAGTCTGCCCACTAA	TTCAGGTTTTCTAAGGGTGTCAGG	Y
NM_006982	8092	ENSG00000180318	CART1	12	488	AATATTTCATGGGCCATGTAA	AACTCCCCAGAACACCCTGATTCAATA	Y

NM_003921	8915	ENSG00000142867	BCL10	1	2030	TTAAGATCACGTACTGTTTCACGACAATGA	GTAACTTTTTCTGTGCTGCATTAATGAACT	Y
NM_001100392	138046	ENSG00000184672	RALYL	8	1112	TTATTTTCTCAGTTTCTACAGATAAAGTGA	GAGTGTGGGCATGTAGAACCTATGAT	Y
NM_002163	3394	LRG_294	IRF8	16	1503	TTCAGAGAAAACCAACAGATCACCGTCTAA	CCTTAAAATCCCAGTGCGATATTGTTTCAG	Y
NM_152991	8726	ENSG00000074266	EED	11	928	GAAGTAGAAGATCCTCATAAAGCCAAGTAA	TTCACTTTAGTTCTAGAGCCTATACTTAAT	Y
NM_001139515	117154	ENSG00000126733	DACH2	X	627	CAACAGTTGTATTCAGCCTGA	AACTGTGTTCCTATCTGCTTGAAAATAACA	Y
NM_001083589	1875	ENSG00000133740	E2F5	8	836	CTGTTTGATGTCCAGATACTAAATTATTAG	TATTGTTGTCTGTGTTTTTAAATATAATAC	
NM_006372	10492	ENSG00000135316	SYNCRIP	6	961	ACTTTTGGGCAACAGTGGAAGTAG	ATCTGAGTTTTAGGTTAGTTAGGATAAACT	Y
NM_001451	2294	ENSG00000103241	FOXF1	16	1559	ATCAAGCCTTGCGTGATGTGA	AAAAGAGGAGGATTTGCCAAAAAAGAAAAA	Y
NM_001159378	64779	ENSG00000103248	MTHFSD	16	2015	CAGCAGAGGGACAAGTGA	CTTATCACGTGTGAGTGACACAATCC	Y
NM_002140	3190	ENSG00000165119	HNRPK	9	1504	AAGCAGTATGCAGATGTTGAAGGATTCTAA	GTAATGAGGAAAGCTCTGGCTGACTATAAG	Y
NM_005250	2300	ENSG00000176678	FOXL1	16	2117	ACGGTACTCCACTTCCAGTAA	TCTCCACTCCTTCCCCTCCAT	Y
NM_018433	55818	ENSG00000115548	JMJD1A	2	777	GAATCCAGTTTTGGCAAACCTTAA	TAGCTTTGTAATAATCACTAAGAAAATCAC	Y
NM_021145	9988	ENSG00000135164	DMTF1	7	1372	GATGTCGAAGATTTGGTAAACTGTCATTAG	GACATATCCTAGATCACTTTGCTTTTTCTT	Y
NM_002753	5602	ENSG00000109339	MAPK10	4	2416	GCACAGGTGCAGCAGTGA	CAGTAAAGAATTAGAGATACAGAGAGGAGA	Y
NM_001122757	5449	ENSG00000064835	POU1F1	3	441	ATTTCTAAGGAACATCTTGAGTGCAGATAA	GTTAATTTTTGAATTGGATATTTCTGGTGA	Y
NM_022818	81631	ENSG00000140941	MAP1LC3B	16	1857	TTCGGGATGAAATTGTCAGTGTAA	CAATGCTCATCCACGTGAGTGC	Y
NM_007013	11059	ENSG00000123124	WWP1	8	1236	ACAGAGGGATTTGGACAAGAATGA	TGCTAAAAGTAGCATTAATATCATTCACAT	
NM_015021	23036	ENSG00000188994	ZNF292	6	2067	GGCAGAGGTCAGTACTGA	TAGGGCAGTTTTGCCAAGTGC	
NM_017869	54971	ENSG00000172530	BANP	16	837	GCCATCCAGATTCAGTGA	GGAAACAGAACTGACTGTCCCG	Y
NM_004329	657	LRG_298	BMPR1A	10	1649	ATGGTTGAATCCCAAGATGTAAAAATCTGA	GATGTGATATAATCACATTTATTTTATGTT	
NM_004836	9451	ENSG00000172071	EIF2AK3	2	1175	AGCCCTTTGCCAAGCAATTAG	GAAATGATGAAAAAGATACCTGTCTGAAAT	Y
NM_001080487	390748	ENSG00000205022	LOC390748	16	596	AAATTCTCACCATGGTTTTCACCGTATTAA	TGTGTCCCGTCCCACGGG	Y
NM_000297	5311	ENSG00000118762	PKD2	4	2265	GGGAGTTCTAATGTCCACGTATGA	TGGGTACCTGAATTGTGTAGCTCG	Y
NM_001160103	79882	ENSG00000100722	ZC3H14	14	1938	AATTTTTGGTTCTGTTCATTCAGCGAATAG	TTGTGGTCCCTTTTTGTGGTGATCATTTTA	Y
NM_001160367	8558	ENSG00000185324	CDK10	16	852	AAGCGCTGTAAACCCTGA	GAGGGCAGGGTGCATCCA	Y
NM_006813	10957	ENSG00000146278	PNRC1	6	1144	TTAAAAACGCTCCTCAAAGTTCAAACTTAG	CTTCTAAATGAGCTTCCCTTCCATTTGTGA	Y
NM_001098173	11105	ENSG00000126856	PRDM7	16	1903	AAGAGATCAAAGAAAGGTCCAAACTCTTGA	GTGTAGCTGTGGCAGGCCA	Y
NM_052996	NULL	NULL	PRDM7	16	1965	GAATCAGGAGCGGCAATATTCTGA	GTGTAGCTGTGGCAGGCCA	Y
NM_018670	55897	ENSG00000166823	MESP1	15	457	CCTGAGGAGCCCAAGTGA	TAGATGCTGAGCCAGGGCT	Y
NM_001039958	145873	ENSG00000188095	MESP2	15	597	CTGGGCATCTTCTACTAA	CAAATGAGGGCCCACCCAAA	Y
NM_004938	1612	ENSG00000196730	DAPK1	9	1450	AGCTCTGTTGTATCCCGGTGA	TTTGAGTAATAATCGGCATCTCAATAACCC	Y
NM_012115	9994	ENSG00000118412	CASP8AP2	6	807	AACTTTAGCATTTTCTTACATTACAGATAA	CTCAATCTACTCAGAACCATTCAGCCA	Y
NM_004755	9252	ENSG00000100784	RPS6KA5	14	1431	CAGTTCTCGGACTCAGTAGCTTAG	ATAAAATAAACATGTTGGGCATTATCTCTT	Y
NM_032186	84146	ENSG00000122482	ZNF644	1	1678	CTAATGGCCGAAGCAGCTTCATAG	ACATACATTATATTGACCAATGAGGTGATT	Y
NM_001134420	8317	ENSG00000097046	CDC7	1	1509	CATCCATTTTTTAAAGATATGAGCTTGTGA	TCCTCTTTAATTTCTGAATATATGTTAACA	
NM_004239	9321	ENSG00000100815	TRIP11	14	1954	GTTGTGCTGAAAGACCTTTTAAAGCAATAG	AAACTAGTATTTCATTTCTCCACACTGAAA	Y
NM_014391	27063	LRG_379	ANKRD1	10	946	ACCTCTCGCATAGCTACATTCTGA	TGTATACATATTAAATACTGGGAGTGCTGT	
NM_005654	7025	ENSG00000175745	NR2F1	5	418	ATGTCCATCCAGTGCTCCTAG	CCTGTACAGAATATATCCACATCCGTCCAC	Y
NM_005263	2672	LRG_63	GFI1	1	1448	CAGCATGGGCTCAAATGA	AAACCCGAGCAATTGGATTTTACTCTTCTG	Y
NM_024832	79890	ENSG00000100599	RIN3	14	917	GAGCCCAACTTCCTGTGA	AGGGTGGCTGAGAGTATGCC	Y
NM_024116	79101	ENSG00000166012	JOSD3	11	411	CAGAGAGGCCTGAAAATGTGA	ACAGCATTTCAAATCCCCTCTTCAAGAT	Y
NM_004268	9440	ENSG00000042429	CRSP6	11	1446	GCACTTAGCCCTTGTCTACTATGA	ACCAAGGCAAATTTTGCAGGCAA	Y
NM_001164391	22823	ENSG00000143033	MTF2	1	2234	GGAGCAACTGCATCCTGA	ATGAAGAAAATGAAAGCAAAGTTTTATAAA	
NM_003972	9044	ENSG00000095564	BTAF1	10	1570	CTGGAAAATTTTATGCATTCTCTCAAGTAA	TAAAAAAATATTCAACTCAAATATACTGAA	
NM_003348	NULL	NULL	UBE2N	12	1907	ACTAGGCTATATGCCATGAATAATATTTAA	ATTTTTAAAGTGAGATTCAATTTCCTTAGC	
NM_005384	4783	ENSG00000165030	NFIL3	9	481	ATCTCTGCTTCAGACTCTGGGTAA	AAAACAAATTATTTGCTGAACTTAGCACTT	Y
NM_004560	4920	ENSG00000169071	ROR2	9	1246	GTCCAGCTGGAAGCTTGA	GGGATATCCCCATTAGCATGGG	Y
NM_203390	389677	ENSG00000183808	RBM12B	8	2082	CCCCGAAAAGTTAAGTTAACTTTGCTGTAG	CCAAATGAACCATCTATACAATCTTTGCTC	Y
NM_000783	1592	ENSG00000095596	CYP26A1	10	760	CATTTCCATGGGGAAATCTGA	CCTTTCCTAATACTGTTTCCATATGCAACT	Y
NM_017948	55035	ENSG00000198000	NOL8	9	658	GACGCAAAAAGGAAAATGAAACCAAAATAA	TTGGTGTACATCTAGATGTTGTTCCTTAAT	Y
NM_014057	4969	ENSG00000106809	OGN	9	1833	AAAAGATTACCGATAGGGTCATACTTTTAA	AATTTGAGAGTTATTTGATGGTGTTTTGCT	
NM_001128429	56916	ENSG00000163104	SMARCAD1	4	1938	ACATTACTAAAAACATCAATGGGCCTGTGA	AGAATACATCCTAAGTAGTAAGTTCCAATT	Y
NM_002612	5166	ENSG00000004799	PDK4	7	2324	GCAAAAGAAGTGGCCATGTGA	TTAAATTTCCAGAAATATGCAGAAACATAT	Y
NM_173849	145258	ENSG00000133937	GSC	14	448	TTGGACTCGGACAGCTGA	ATTTCTTGTTTGTTTGTTTGTTTAGGTAGA	Y
NM_006744	5950	ENSG00000138207	RBP4	10	419	GATGGCAGATCAGAAAGAAACCTTTTGTAG	TTCAGGAAGTTGGGAGAAGCCAGTATTTAA	Y
NM_007084	11166	ENSG00000125285	SOX21	13	1774	TACGCTGCCGCGCTATGA	ACAATGGCTTGATTTAGATTTCCTAGTAAA	
NM_001012267	401541	ENSG00000188312	CENPP	9	2186	TGTGCAGAGGAGAACAACTAG	CCTCGTGGTCGCGCAGAT	Y
NM_001127362	7181	ENSG00000120798	NR2C1	12	586	TTCACAAGACGAGCAATCACTGATTTATAA	GACTCAAATTTTGGTAATAGGATTTTATTT	
NM_001256427	10611	ENSG00000163110	PDLIM5	4	2381	TTGATTTGGTTTTTCTCTTTGTTTCCAGAA	CTGAGACATTTTGATAAGCAAATATCATAC	
NM_001261458	83744	ENSG00000127081	ZNF484	9	1377	CAAGGCCAACTTTCTTCTATCTAG	GTGGGTTAATTTAAATGGATATTGACTCAA	Y
NM_032427	84441	ENSG00000184384	MAML2	11	852	CTTGATGAAATCTTGGGGAACAATTCCTAA	CAATGTCCACTGGGTTTTTTAAAAAAGTGA	Y

NM_001122825	54845	ENSG00000104413	ESRP1	8	1715	ACTTTTTTTTTTTTTTTTTTTCAGTGTTTGA	CCAGGCCATCTTGAGTAGATTTATCACT	
NM_032788	84874	ENSG00000144026	ZNF514	2	1807	AGAAGTCATGCTGGAAAAAAAACCCTATAA	GTTTCTGAAGATCTGCCATTTCTGGACATT	Y
NM_001017396	7549	ENSG00000275111	ZNF2	2	2390	GCCAAACAGGGAATAGACTGA	TAAAAAAGATGTCCATTGGAATCCTAAATT	Y
NM_057749	9134	ENSG00000175305	CCNE2	8	1587	GAAAAACCACCAGGAAAACACTAA	ATTTTTGACACACCTCTAATAACATCACTT	
NM_013434	30818	ENSG00000115041	KCNIP3	2	2208	CAGCTGTTTGAGAATGTCATCTAG	CTGAGAATTTGAGCGGCATTCTCT	Y
NM_003095	6636	ENSG00000139343	SNRPF	12	569	GAGGAAGAAGATGGGGAAATGAGAGAATAG	TCAGTTCCCAATTTCTGTTGTGCC	Y
NM_018063	3070	ENSG00000119969	HELLS	10	657	GAAGATTCCAGTCCTGAATGTTTGTTTTAA	AACACAATGTCCACTTGAAATCTCTATATG	Y
NM_018343	55781	ENSG00000058729	RIOK2	5	2374	GCCAGCTTTTGGGGAGAATAA	TAAAAGAATGATTATCTCGAGCTGCACTAA	Y
NM_005222	1750	ENSG00000006377	DLX6	7	1173	CAGAGACCACAGATGATGTGA	TGATGTTCATATATATACATATGACTTCCC	Y
NM_005221	1749	ENSG00000105880	DLX5	7	526	GCCTCCGGGACACTCTATTAG	TACATATACAGTCTGCATGCATAGAGCC	Y
NM_005230	2004	ENSG00000111145	ELK3	12	828	CTGCTTTCTTCAAACTCTCAGAAATCCTGA	CCTGGAATAGCATGAACTTGATGTAGCTTT	Y
NM_002595	5128	ENSG00000059758	PCTK2	12	2161	AAGAACAGAAGACAGAGCATGCTCTTTTAA	TCACTGCAGTGTTTTCAGGATCTGAAT	Y
NM_021570	56033	ENSG00000131668	BARX1	9	717	AGGAGCCGCGAGGACTGA	GGCTTGATGGTGAAGAGCACA	Y
NM_020992	9124	ENSG00000107438	PDLIM1	10	537	ACTGTGTTCCCCAAGTGA	TGTAAGAACAGAGCTTGTTAAGATGCAAGT	Y
NM_001170807	9457	ENSG00000112214	FHL5	6	1044	GGAATGGACACTGACATCTAG	ATGTCTGCCCATTAATCATTTATGTAATTT	
NM_194320	169841	ENSG00000175787	ZNF169	9	1819	CTACCCCAAGAGGTCTTCTGA	AAGGTTCTGGGCTGGGTGT	Y
NM_212481	10865	ENSG00000196843	ARID5A	2	501	CACCTCAACACCAAGCTGTAG	AGGCTGGGGTTTGCTTTGG	Y
NM_021190	58155	ENSG00000117569	PTBP2	1	1824	AGAGTGTCTTTCTCCAAGTCAACAATTTAA	AAAACAACAAGAACAAAAGAGATAGAATTA	
NM_003384	7443	ENSG00000100749	VRK1	14	586	TCAAGAACCAGAAAGAGAGTCCAGAAGTAA	AGATTTTCTCTCTCATGGATACGCTTCCTC	Y
NM_173655	285220	ENSG00000080224	EPHA6	3	427	CTAACACTTAACCTCTGCTATTCTGCATAA	CTTTTTTCTCGCTGCAAACTTTTTACACCA	Y
NM_001258441	29760	LRG_21	BLNK	10	454	AGACTGAAGTATGCAGTTAAAGTTTCATAA	TAAAATTTTAACCAAGACTTAATTGTCAAG	Y
NM_001270	1105	ENSG00000153922	CHD1	5	1356	GAGCATACCTGGAGTAGTCGGAAAACATAA	TAATGCTACACATTACAAATATTCAACTGC	Y
NM_207519	7535	LRG_126	ZAP70	2	542	GAGGCTGCCTGTGCCTGA	CCCTAGGGTGGAGAGTGGGA	Y
NM_178861	140432	ENSG00000139797	RNF113B	13	558	TTTTTTGTTCTTTTAGGAAAAAAGAGATAA	TTTCATTTAGCAAAACTCAAGCTCTGTACC	Y
NM_006693	10898	ENSG00000160917	CPSF4	7	1053	TTTCTCAGTGGACAGTGA	CCCAGACTTGACCAGCTGCTT	Y
NM_145102	23660	ENSG00000196652	ZFP95	7	1753	AGTGTAGAGGGGTCTCTGTTGTAG	ATTAACAGTGATGCGGGTCTTCACT	Y
NM_153695	195828	ENSG00000165244	ZNF367	9	2474	GCGCCACTCCGACAGTAG	TACGCAAACATGTACTGAAATAATACTGAT	
NM_001085367	79027	ENSG00000197343	ZNF655	7	826	ACTCCTTTTCCACGATTGTGA	GTGTAAAAGTGTGTATGCATGTCGACT	Y
NM_014282	22927	ENSG00000130956	HABP4	9	1478	GATTTCCCTGCGCTGTCTTGA	AATAAATAAGAGACCAAAAGTAGGCTATCA	Y
NM_001001662	158431	ENSG00000196597	ZNF782	9	1112	GCCCACCCAGGGGATTAA	GCCATGGTCATTCAGGATGCTGT	Y
NM_145914	7589	ENSG00000166529	ZSCAN21	7	603	GAGGGAGAAGCACCGTAA	CTTCCAAGGTGCTGGGATTACAG	Y
NM_032924	7551	ENSG00000166526	ZNF3	7	1352	TTAAATATCAGAGAGTCCACGTGA	TCAGGCTGGTCTAGAACTCCAGA	Y
NM_006833	10980	ENSG00000168090	COPS6	7	590	CGCGGGCTCTTTTTCTGA	GCTGCCTCTCCAAAATATGTAGTAAACG	Y
NM_001142462	116039	ENSG00000164920	OSR2	8	577	CCGCGGCAGGACTTCTAG	AAGTTGCAGAATGAGAATTCTGAGAAATCC	Y
NM_016316	51455	ENSG00000135945	REV1	2	946	ACTTATGGAAGCACATTAAAAGTTACATAA	TTCCTGATATTTGGGCTTAGTGCTTCTAAA	
NM_021620	59336	ENSG00000112238	PRDM13	6	999	GGGGAGCGCGACTTGTAA	TGGTATATTTCCCTGTTATAATACAACACT	Y
NM_030935	81628	ENSG00000166925	TSC22D4	7	620	AATGGGCCCTCCGTCTGA	CGCTGGCTCCCTTCTCCA	Y
NM_000673	131	ENSG00000196344	ADH7	4	1067	CGAACGGTCCTGACGTTTTGA	GCATTGGAAGCCTCAATTTCTGCTATG	Y
NM_001195479	10342	ENSG00000114354	TFG	3	616	CCTGGACCTGGTTATCGATAA	ACCAAATGGCACTGTTTTCTCTGAAT	Y
NM_001168474	54457	ENSG00000102387	TAF7L	X	1120	TTGCAGCGTTTTCTGAAGAAGTGA	AAATTTCTGTTGTTTATATTTACCCAGTCT	Y
NM_000061	695	LRG_128	BTK	X	618	ATTCTAGATGTCATGGATGAAGAATCCTGA	CAGAGGCAGGAGTTAACAAGAGG	Y
NM_004473	2304	ENSG00000178919	FOXE1	9	1859	TTCGTGTCCGCCATGTGA	AGAGAAGCTGTAAGTGTAAAACACACTCCA	
NM_007129	7546	ENSG00000043355	ZIC2	13	1263	AACTTCAATGAATGGTACGTGTGA	GGCTTGGTTTCTATGCAGACACAC	Y
NM_003403	7528	ENSG00000100811	YY1	14	1047	AAGGCCAAAAACAACCAGTGA	TCAATAATCACTGTAATTGAGTGCAAAATA	
NM_003729	8634	ENSG00000137996	RTCD1	1	1445	GGAATTGGGATGACAAATCCAAATCTATAG	AATTACACACTCTATTGTAGTACAACACAT	
NM_005068	6492	ENSG00000112246	SIM1	6	1667	ACATCTGTTATAATAACCAACGGAAGCTGA	TTTGGGCACGGGACTCCTA	Y
NM_001206977	9971	ENSG00000012504	NR1H4	12	558	ATCTGGGACGTGCAGTGA	GAATGACTATTCACCAGGACTTTTCCAGAG	Y
NM_145285	159296	ENSG00000119919	NKX2-3	10	982	GGCATCCGGGCCTGGTAG	AGCCTGGGGCCTGGCTCA	
NM_014415	27107	ENSG00000066422	ZBTB11	3	1907	GAGGTAGCACATATTTCAGGAGGAGAATGA	ATGTAAGAGTAAGAAGTTTTAATATTGTGT	
NM_000693	220	ENSG00000184254	ALDH1A3	15	2032	GGCGACAAGAACCCCTGA	GCACTGGGCCCGACTGAC	Y
NM_024652	79705	ENSG00000154237	LRRK1	15	1444	TGCACTCGCAAGAGAAGGTAA	GTCTCCACCCATGGAAGCTG	Y
NM_002518	4862	ENSG00000170485	NPAS2	2	1423	CAGCAGCCGCCCCGATAA	CCGATGCACATATTGCAGCTCAAAAA	Y
NM_002568	26986	ENSG00000070756	PABPC1	8	1562	ACCGGTGTTCCAACTGTTTAA	ATTGTCTTGTGCCTTTTACATAAAACCAAT	Y
NM_018445	55829	ENSG00000131871	SELS	15	766	TCTGGCGGATGAGGCTAA	TTCTTTATCCCAGTAATTCGGCAACTCG	
NM_152323	121599	ENSG00000166211	SPIC	12	406	TACCATGAGCTAAATCACCATGATTGCTAA	GGCTGAGACATCTTTAAAAAATAATAAAAT	
NM_001202546	1523	ENSG00000257923	CUX1	7	1048	GGTGACTTGTGGCAGTGA	TGTACTCATGGAATTCCTGGAGATGAGAAG	Y
NM_001278	1147	ENSG00000213341	CHUK	10	1411	AATCTTGATTGGAGTTGGTTAACAGAATGA	TACTACCTTTACCATAAGTCCTATTATTCC	
NM_001142530	80823	ENSG00000198908	BHLHB9	X	1982	GAAGTTAAAGAGATTATTGAAACAATGTAA	TAAGTGTTAAGAGGAAAAGGCAAGTCTCAA	
NM_001177948	50511	ENSG00000139351	SYCP3	12	459	CGGAAGTCTCTTCAATCCATGTTATTCTGA	TGTCATTAGAATCGGTTGTACAACTTAAGT	Y
NM_058170	118427	ENSG00000118733	OLFM3	1	1847	CATATCATCAAGACAGAGGATGACACATAG	GAATCTCTTAAATTTTCCAACTCCCAGTAG	
NM_003989	5076	ENSG00000075891	PAX2	10	2438	CACATCGTCCCCGTCTGA	CAGCTGCCGCCAAGGGGG	

NM_173199	8013	ENSG00000119508	NR4A3	9	707	TTATGGCTACTAGTAATAAGAGTTGATTGA	CAGACTGATGGGATTTTACTATATCCCATA	Y
NM_001195517	3195	ENSG00000107807	TLX1	10	1298	GGTGCTCCTGGCAGGTAA	TTCCTTTCCTCTCTCCAGAGCCTTA	Y
NM_001142428	9643	ENSG00000123562	MORF4L2	X	843	CACCGCAAAGCCCTGTGA	CCTTGAAGGATCCAGTAGAATTACATGATT	Y
NM_006562	10660	ENSG00000138136	LBX1	10	474	GAGATCGACGTGGACGATTGA	AGCTGCCACTCCCCTAGAC	Y
NM_001204453	5701	ENSG00000161057	PSMC2	7	555	GAAGAAGGGATGAGAGTGGGGTAA	TTTATCCCCAAAACCATACTTTAAGAAAAC	Y
NM_002803	5701	ENSG00000161057	PSMC2	7	1521	GCTACTCCTCGTTACATGACATACAACTGA	TGGCCGATTAGATTTTCCTTATGGACCTAA	Y
NM_004316	429	ENSG00000139352	ASCL1	12	1741	GACTTCACCAACTGGTTCTGA	TGGATGGGACTGGCCATAGC	
NM_006035	9578	ENSG00000198752	CDC42BPB	14	1513	CCGGCCTGTGACACCTGA	TTGGGTGACCTCCGAGTGAGTTA	
NM_003998	4790	ENSG00000109320	NFKB1	4	888	CAGGAAGGACCTCTAGAAGGCAAAATTTAG	AAACACTTTCCTTTTGATAATTATTTGTTC	
NM_001032282	7071	ENSG00000155090	KLF10	8	1535	CCTGCTCCCACACAGTGA	TCTCTAAATGGATTCATGTTAAAGACATAC	Y
NM_002553	5001	ENSG00000164815	ORC5L	7	687	ATAATAAAATACTTGTATGATTTCTTGTGA	TGGGTAGCTAATTTTAAGAAAGTGACCAGA	
NM_003893	8861	ENSG00000198728	LDB1	10	705	TCACAGGCCTCCCAGTAA	GTCTGAAGCTCTCACACCATATAAAATGGT	Y
NM_015062	23082	ENSG00000148840	PPRC1	10	484	CAGAAGAACCTCAGGAGGTAA	CTCATTTTAGACAATACATCTGAGGAACCA	Y
NM_001128921	4140	ENSG00000075413	MARK3	14	782	TCCAAAATTGCCAATGAGCTAAAGCTGTAA	CTCTTTATTGCCTTGGGAGAAAAAATATGC	Y
NM_005029	5309	ENSG00000107859	PITX3	10	505	GTGGAAAGGCCCGTATGA	CCCCGACCTGGAGTAGGG	Y
NM_005432	7517	ENSG00000126215	XRCC3	14	1361	GGGACCCAGTCCCACTGA	AGCTACTTAACGGTTCTGGGCC	Y
NM_197977	7743	ENSG00000136870	ZNF189	9	1191	TTGCACACAGCATGGATGCAATAA	CCTGGGCTTAATTGAGCTACTCCAG	Y
NM_019592	56254	ENSG00000155827	RNF20	9	1102	AATGATTTTCATCGCATCTACATTGGTTGA	TAAGCAAAGATCTAAATACTCTAAACAAGG	
NM_001178133	51684	LRG_521	SUFU	10	572	TATGTTTGACATCCTCAGCTCTGA	CCAGGGCTCCTTGGAAAGG	Y
NM_182931	55904	ENSG00000005483	MLL5	7	932	CATGGGTCAGGGTGGCATTAA	CCACCACAGGAACAGCCC	Y
NM_182691	6733	ENSG00000135250	SRPK2	7	1642	CGGCATCCTTGGTTGAATTCTTAG	AATAAATGATTTCCTTATAATACGCCTCAA	Y
NM_017649	54805	ENSG00000148842	CNNM2	10	1587	AACGAAGGCGCCATCTAG	AGAAAACGCTCTCTCCTTTCCTAGTGAT	Y
NM_006951	6877	ENSG00000148835	TAF5	10	1022	CTAGCTGCAGGAGCTTATAGTCCACAATAA	AGAGTAAAGCTGGCCACATTTGTATCTAAA	Y
NM_005163	207	LRG_721	AKT1	14	1171	GCCAGCGGCACGGCCTGA	ACACAGCCTGTCCCCAAAC	Y
NM_001014431	207	LRG_721	AKT1	14	1171	GCCAGCGGCACGGCCTGA	ACACAGCCTGTCCCCAAAC	Y
NM_001137601	100128927	ENSG00000179627	ZBTB42	14	2425	GTCAAGTCCCTTCTGGTGTGA	CTCCCCCAAACGCTGGCA	Y
NM_001251905	55198	ENSG00000136044	APPL2	12	1197	GCAGAATCCGAAGCATAA	CTACTCATGTTGACTGGAAGTATTTTTTGA	
NM_004689	9112	ENSG00000182979	MTA1	14	685	CCCATCGTCATCGAGGACTAG	TGGCTGTCCTGGCTGAGG	Y
NM_001312	1397	ENSG00000182809	CRIP2	14	697	GGCAAGGTCCAGCCCTAG	AGAAACATCCCAGGGCCC	
NM_201557	2274	ENSG00000115641	FHL2	2	637	TGTGGGAAAGACATCTGA	TGTGATGCTGTTCCAGTTGGCAA	Y
NM_018301	55285	ENSG00000089682	RBM41	X	1404	GGTAGCACTACAGAAATTAGTGGTAGCTAG	CTGCCATTGTTTCTGAGGGGAAGT	Y
NM_004849	9474	ENSG00000057663	ATG5	6	2243	ATTAGTATCATCCCACAGCCAACAGATTGA	TATTGGGGGTCTTCTCCAATTTTCTTTTAT	Y
NM_012082	23414	ENSG00000169946	ZFPM2	8	1181	CATGCAGCAGAACATGTCAAATGA	AATTAGGTGTATGTAAAGGACAATCTTTCT	
NM_012257	26959	ENSG00000105856	HBP1	7	1278	TTATTTCCACAGGGCTCACAACAACATTAA	TAATTTTTAAAATCCTATCAGCAGCCTCCT	Y
NM_004089	1831	ENSG00000157514	TSC22D3	X	1477	GGTGGTTCTGCGGTGTAA	AGTATTGTGCAGATGACACGAGATCA	Y
NM_001163437	93627	ENSG00000145348	TBCK	4	674	ACCATCCCATCTCCTCAAATATGA	TTTAAGCCATTCGTCTTTGCTACAGGAA	Y
NM_001206691	5992	ENSG00000111783	RFX4	12	1515	TCTACAGGATGGGCTAAATGA	ACACAGCCATATAAGACTACTTAGTTTTTT	
NM_138775	91801	ENSG00000137760	ALKBH8	11	2111	GTGATTCTTCAAAAGGCCTGA	TCACTCTACTGTTGATGGACATTTGGACTT	
NM_004075	1407	ENSG00000008405	CRY1	12	1602	CCTAAAGTCCAGAGACAGAGCACTAATTAG	TTTTGAAAAACACTGTTTAACTAAACATAT	Y
NM_000111	1811	LRG_683	SLC26A3	7	568	TTCCAGGTGCCAGTTGAAACAAAATTCTAA	CTGGTAATCATAAAATCTGAGCAAAGAAGC	Y
NM_139166	137735	ENSG00000174429	ABRA	8	1734	GTTGTGATTACGCTACTCAAGTGA	GAGGAGACTCTTGAAACCTTCATCTATTAA	Y
NM_002519	4863	ENSG00000149308	NPAT	11	1730	TTTTTGTTATCATTGCATTATGATGAGTAA	TGAATTTTCAGTGCTTAGTACATACAACTA	Y
NM_012406	11108	ENSG00000110851	PRDM4	12	1524	GCGGATGAGTCTCTTTCTGCACATAAATAA	AACAGCAGAAACAATAAGAATATGATCTCA	Y
NM_182529	168451	ENSG00000177683	THAP5	7	2144	TTTACAACATATGAAGTCACTATGATATAG	GTCATGTTTGTGCTCAAAAAGTTTCAGATT	
NM_000051	472	LRG_135	ATM	11	3771	TGGAAAGCTTGGGTGTGA	GCGTGGTCAGTTGCCTATCCT	
NM_005421	6887	ENSG00000186051	TAL2	9	461	CCAAGCCACCACATTCCTTAG	TGATCAAATCTCATTTTTCAAAACAAACTT	
NM_003269	7101	ENSG00000112333	NR2E1	6	1526	TCAGATATGTACAAATCCAGTGATATCTAA	ACTGTAAGAAGGAAATTGCCTTTGTAGTTC	Y
NM_206937	3981	LRG_79	LIG4	13	1269	TTACAAGAAGAAAACCAGTATTTGATTTAA	GAGCTGAGATTGTGCCACTGCA	Y
NM_014706	9733	ENSG00000075856	SART3	12	1423	GCCAAGCTGTTTCTGAGAAAGTGA	GTGAAGGCCTTTCAGTCCTCCT	Y
NM_001130714	51176	ENSG00000138795	LEF1	4	1307	CTGGAGATGGAAGCTTGTTGA	ATTTACAGCCGGTTCTGGCCT	Y
NM_213596	121643	ENSG00000139445	FOXN4	12	1873	AAGCCTATAGCCCTGCTTTGA	GGGAACTTGGTGTCAGAATGCCT	Y
NM_004235	9314	ENSG00000136826	KLF4	9	1079	CTCGCCTTACACATGAAGAGGCATTTTTAA	GTTTTTTTTTAATAAAAAAAGGTATTTTAA	
NM_001128167	5063	ENSG00000077264	PAK3	X	678	GCAATTAAGAACAGCAGCCGCTAA	AAACCAAGCCACCACTATAGAAGAAGAATA	Y
NM_006492	257	ENSG00000156150	ALX3	1	538	CTGAACTGGACCACGTGA	CCTTTACACCCTCCTTAGTGTCAGAC	Y
NM_001744	814	ENSG00000152495	CAMK4	5	764	GATGTGATCCTGCCAGAGTACTAA	GAGAGCAGGTGAAGGAAAGAATGGAA	Y
NM_022768	64783	ENSG00000162775	RBM15	1	1252	AACTTGGCGCTGACCCTGTTATAG	TCAGAATTGACACACCGTGATAGTCAAAAT	Y
NM_006235	5450	ENSG00000110777	POU2AF1	11	2185	ACTCTCTCTGTGGAAGGCTTTTAG	ATCTTCTCAAATGCTGTCTTGAACTTTTAA	
NM_001267728	NULL	NULL	ING1	13	1322	AAAGAGAGGGCTTACAACAGGTAG	ATGCTAGTCTAAATCAGAGGTCAGCAAA	Y
NM_000325	5308	ENSG00000164093	PITX2	4	881	GTGGACCGGCCCGTGTGA	GCTATCATTTATTTCTACCAAATTTAAATA	Y
NM_015267	23316	ENSG00000111249	CUTL2	12	2409	CTGGAGTGGGAGTTCTGA	GCCTCCCAAAGCACTGGGATTATA	
NM_002973	6311	ENSG00000204842	ATXN2	12	778	CACCAACAGCAGTTGTAA	TCTAGAGATACCTTGAACCAGAACTAAGGG	Y

NM_153048	2534	ENSG00000010810	FYN	6	1587	TACCAACCTGGTGAAAACCTGTAA	ACTCTCCAGATGTTCTGAATGTTTCTGTCA	Y
NM_024102	79084	ENSG00000116455	WDR77	1	1520	CCTGCAAGTGTTACTGAGTAG	TTACTTTCTCTAAGTGTCCACAAAATTAAA	Y
NM_001037290	83875	ENSG00000197580	BCDO2	11	1232	CATGGTACCTTCATACCCATCTGA	TAAAAAAGTGTGCCTCTAATCTAACTCAGC	Y
NM_001127511	324	LRG_130	APC	5	2293	TCTGGGTCTTACCTTGTGACATCTGTTTAA	ATTTGAATTAAAAGGCAAGTTTATCTAATT	
NM_007204	11218	ENSG00000064703	DDX20	1	858	CTACAAGAAATGATGCATAGTAACCAGTGA	CCTTGAAATTACACTCACAATTCTGTGCTT	Y
NM_003668	8550	ENSG00000089022	MAPKAPK5	12	543	TCCCACGAATCCCAATAA	TCCATCATGGAAGTTGGCTCC	Y
NM_006343	10461	ENSG00000153208	MERTK	2	684	GGCTCAGAAGTCCTGATGTGA	TGGTGTGGTTTTTCCATATCTCTATAAAGC	Y
NM_022828	64848	ENSG00000047188	YTHDC2	5	2084	GGAGAAAAAAACACAACTGATTGA	TTACAGGGAAGGGAGTTTCAATACAAAC	Y
NM_175744	389	ENSG00000155366	RHOC	1	593	GGCTGTCCCATTCTCTGA	GAGCCAGGCATGACCTCATC	Y
NM_024019	63973	ENSG00000178403	NEUROG2	4	1321	CCCATAGCCAGGGATTGTATCTAG	TGTGATTTCTTAGTGATATTCTACACAAAG	Y
NM_004416	1840	ENSG00000135144	DTX1	12	1269	GCTGCAGCCAAGGCTTGA	GTCTGCCACTGCTTCACC	Y
NM_015454	51574	ENSG00000174720	LARP7	4	445	AAACATATAAGATTTTCTGAATATGATTGA	AACATGAAAAGGTATTGAAGGTATTGCAAG	Y
NM_024072	79039	ENSG00000123064	DDX54	12	1884	ATGCGGAAGAGGATGTGA	GCATTATTTTTCCAGTTGCCCCCTAGTTAT	Y
NM_022363	64211	ENSG00000089116	LHX5	12	481	GAAGCCGCCGTGTGGTAA	GGAAAGCACAGCGAGAAATAAGATTACAAA	Y
NM_015642	26137	ENSG00000181722	ZBTB20	3	1085	AGGATGCATGTGTCTGACGGATAA	TAAAAAAATCAGCATTCTATCTGGTAAACG	Y
NM_016090	10179	ENSG00000076053	RBM7	11	1286	TGGCGCTCATCTCGACACTAA	CTATATGTACAAAAGATGTATTTCAGGATG	Y
NM_007111	7027	ENSG00000198176	TFDP1	13	1386	GAGAATGACGAGGACGACTGA	ACAACATGTGGTGGTGGGGTTT	Y
NM_133464	158399	ENSG00000173258	ZNF483	9	1442	GGATTTCACTCTGCAGAGTAA	ATACAGTGGGAAAGTCAGGTGACATACA	Y
NM_001007169	158399	ENSG00000173258	ZNF483	9	1610	AAGATGCAGATGTTCTCTGAGGCTGAATAA	CGCTACTCATTAGCAGTCACTCTCCATT	Y
NM_002929	NULL	NULL	GRK1	13	481	TCAGGGATGTGTCTGGTTTCCTAG	CCAGGGTTCACTGACCCCTG	Y
NM_080717	6910	LRG_670	TBX5	12	1782	GAGTGGAGCGACAATAGCTAA	CCCAAATTCTCCTGCGATGG	Y
NM_001146274	6934	ENSG00000148737	TCF7L2	10	1885	CTCGTCACCAAGTCTTTAGAATAG	GAGTCCAAGACCACGTTCTGGTATCTAA	Y
NM_003408	7539	ENSG00000136866	ZFP37	9	1011	ACTCATTCAGAAGATAAATCTCATGAGTGA	TGTCAAAATCTGAGATCTCACATGTTGATC	Y
NM_002031	2444	ENSG00000111816	FRK	6	1064	TATTCAGATGCAAATAACTTCATAAGATGA	AAATGCCTAATGTAGACGACGGGTT	Y
NM_005599	4808	ENSG00000177551	NHLH2	1	1767	CACGTCCTGGACGTGTAG	ATCAAATATTTCACTACATAAATATGTTTA	
NM_000245	4233	LRG_662	MET	7	2442	TCCTTCTGGGAGACATCATAG	TGCTGTTTTACTACTTCCTCAGCATTCA	Y
NM_173560	222546	ENSG00000185002	RFXDC1	6	837	GCAGCTGGAGGCACTTAA	GGATATGATATGCAAATGTTTTTAAATTAT	
NM_153453	245806	ENSG00000170162	VGLL2	6	1251	GCATCCCTCCTGAGCTGA	GGAAACACAGAAAGATCTATCTATCTATCT	
NM_003594	8458	ENSG00000116830	TTF2	1	1525	CTCAGAGTCCTTTTTGGCATCTAA	GAGGGCCACCTAGAAGTGACTTACAT	Y
NM_144658	139818	ENSG00000147251	DOCK11	X	533	CCAAGATACGCTGAAGTGTGA	CTAATTGGCAAGGATTCAAATGATCTCCCC	Y
NM_006265	5885	ENSG00000164754	RAD21	8	1746	ACACCTGGACCAAGGTTCCATATTATATAA	CTAAGACCTCTTTCTTTTGAAAAATTCATT	
NM_017418	50514	ENSG00000173077	42339	9	699	CTTTTTTTTTTTTTGTCTCCCAGCAGATTGA	AGACCAAATCTTGAAACCAGAATAGGCA	Y
NM_080651	90390	ENSG00000164758	THRAP6	8	464	ATAAATGCCATGTTGGCAATGAGGAACTAA	TGAATTTAAAAACTTGAGCTGATATGGTAA	Y
NM_016281	51347	ENSG00000135090	TAOK3	12	1376	TTAGATTTTCCTAAGGAGGACTACAGATGA	GAAGACAGAGCTTTACCTGAAAGGCTT	Y
NM_001173488	55922	ENSG00000186416	NKRF	X	1195	CTTGTTATGCCTCAAGCAAATTGA	AAAGTGATAGTTGACTGATTCCTATCTGTT	Y
NM_182557	283149	ENSG00000186174	BCL9L	11	2453	GCCAACCTGCCCTTCTAG	GAAATAACAGCTGTGGCTCACGTGA	
NM_001112704	11023	ENSG00000148704	VAX1	10	898	AAAAAAGCGCTGGACTGA	CTTTGCTTTTCCCCCATTAAATTCCTGTTT	Y
NM_080632	65109	ENSG00000125351	UPF3B	X	1032	AGAAAAGAAGGAGGAGAGGAGTGA	TTTGGGTGAGATGCCTGCTTCTA	Y
NM_001243259	25988	ENSG00000172273	HINFP	11	1093	TGGTTCGCTACGAGAGTGTAG	GAGGAGAAGACAACAGGACAGTTAAG	Y
NM_015517	25988	ENSG00000172273	MIZF	11	737	CCAGAGATCCAGATGGTTTGA	GAGGAGAAGACAACAGGACAGTTAAG	Y
NM_014034	25842	ENSG00000111875	ASF1A	6	1736	TCCCACATGGACTGCATGTGA	CCTAACCCTTTGATGTCACTACAAATAGAT	
NM_001099685	727940	ENSG00000203989	RHOXF2B	X	432	TCTTTCACATTCCCCAATGTCTAA	GAGGAGATGAGTATGGGGTGTTG	Y
NM_004098	2018	ENSG00000170370	EMX2	10	1494	GAGGAAATAGACGTGACCTCAGATGATTAA	ATCTTTCTTTAAAATGCCTGAGAATCCATC	
NM_152380	6913	ENSG00000092607	TBX15	1	1869	ATGTCCGTGCACATGGTTTAA	TTACTACTTATAAAAGGATGCTAATATTCA	
NM_001099679	22954	LRG_211	TRIM32	9	1776	TACCATCTGAGAAGATATTCCACCCCATAG	ACAGATAAAGTGGCTCTAATAACTAGTGGA	Y
NM_033013	8856	ENSG00000144852	NR1I2	3	1453	GGCATCACAGGTAGCTGA	AATTCATTTTTATGTTCTTACGCCGGAGTC	Y
NM_001426	2019	ENSG00000163064	EN1	2	947	GACAAAGACGAGAGCGAGTAG	TCTGGGTCGCGCTTCCCC	Y
NM_014365	26353	LRG_249	HSPB8	12	1068	GAAGTCACCTGTACCTGA	TGCCTCGCCCAGCCTTTG	Y
NM_014352	25833	ENSG00000137709	POU2F3	11	1732	CATTCCACCTACCTCCACTGA	CTTTAGCATCAGTTTGGGGAGTAATACACT	Y
NM_003750	8661	ENSG00000107581	EIF3S10	10	1190	GGATGGACCACAGTACGACGTTAA	TCCTAGCCACATATGCTTTCTTTGTTATAT	Y
NM_005308	2869	ENSG00000198873	GRK5	10	732	TCCACCGGAAGCAGCTAG	GAGGACATTCTGGCTGGGAG	Y
NM_003252	7073	ENSG00000151923	TIAL1	10	2379	ATGGCAAGTTACCAAACACAGTGA	TCACTACATGTACTCCAGCCTGG	
NM_004281	9531	LRG_742	BAG3	10	715	AACCCAGCAGCACCGTAG	CAATTCTCTCTTGAGCCGGGCTATT	Y
NM_000545	6927	LRG_522	TCF1	12	1499	ATGGCCTCTTCCTCCCAGTAA	TAGAGTCTCAGCAGAGCAAGAAAAGC	Y
NM_018699	11107	ENSG00000138738	PRDM5	4	517	ATCCATGGTGTAGCTGACAGCTAA	CCTCATCTGCAAAGAAATAGTTGGATATCT	Y
NM_172214	10645	ENSG00000110931	CAMKK2	12	716	CTTCTCTTTCTTTGATGTGTTTAGACCTAG	CCACAGAGGAATGGATGAACAAAGTGA	Y
NM_005270	2736	ENSG00000074047	GLI2	2	2158	AAGTTCCTGAACATGATGACCTAG	GTTCTGACCCTAGGAGTGTTTGGG	Y
NM_001160264	389549	ENSG00000128610	FEZF1	7	783	CACCAGGGCCACCAGTGA	AGGGATGAGGGGGTCAGAGT	Y
NM_019034	54509	ENSG00000139725	RHOF	12	1924	CTCTGCCTGCTGCTCTGA	GGCACCTGCGTTTGCCCT	Y
NM_002813	5715	ENSG00000110801	PSMD9	12	1734	AACATTATTCCTCTGCAAAGATGA	TGCAGGAAACCAGCCAGAG	Y
NM_032390	84365	ENSG00000155438	MKI67IP	2	959	AAAAAAAGACGAAGAAGCAGCAATCAGTGA	GCAAGCTACCGTGCCTGG	Y

NM_001136239	93166	ENSG00000061455	PRDM6	5	1030	CCAGAATCAATCGAAGTGGATTAA	AAGGGGGCTCCTGGGGTT	Y
NM_001237	890	ENSG00000145386	CCNA2	4	1374	CTCAACCCACCAGAGACACTAAATCTGTAA	AAATTCATGTAGAAATAAATTTTGCAAGAG	
NM_001135564	3298	ENSG00000025156	HSF2	6	1079	GATAGTGATATGCCACTTTTAGATAGCTAA	TCCAGTGATAATATTCTTACACTATTTGGG	
NM_001144917	2263	ENSG00000066468	FGFR2	10	1707	ATAAACGGCAGTGTTAAAACATGA	TGTCACAGGTACTGCTGGTCTCA	Y
NM_003455	7753	ENSG00000166261	ZNF202	11	1888	CACTCAGAAAAGACCTCCTAG	TCTGCAAACTCCAGGCTTGGAT	Y
NM_020382	387893	ENSG00000183955	SETD8	12	1830	CCGTGGCTGAAGCATTAA	TGAGGAAACGAAAGAGCTCAGAGTTC	Y
NM_014109	29028	ENSG00000156802	ATAD2	8	1463	GAGGTAGAAAACTTCAGTTGTTCCAGATGA	TGTACCTAAAATACATGTACCATATAAAAA	
NM_001077261	9612	ENSG00000196498	NCOR2	12	1171	CTCTCCGACAGCGAGTGA	TTACCAAGGGTATAAATATTCAACTTGCAA	Y
NM_005519	3167	ENSG00000188816	HMX2	10	729	TACAACCTATACAACAAGCTCGACTACTGA	CCGACCTTTAACCCCTGACC	Y
NM_001242335	26468	ENSG00000106852	LHX6	9	2363	CTCTTGCAGGTCATCCTTTTTCAGTACTAA	TCGTCAGGCCTCCACAATCCA	Y
NM_003794	8723	ENSG00000114520	SNX4	3	1309	AATGCTAAGGAATGCTTTAGCAAGATGTAA	TGTGTACTCAATGTTTAGCTCTCACTGATA	
NM_022062	63876	ENSG00000165495	PKNOX2	11	2177	CACAGTGACTCCCTGGAGTAG	CTGAGCTCACGCAGGACG	Y
NM_020924	57684	ENSG00000171448	ZBTB26	9	690	TTAAGAAATGATAGTACTTGTGTGAATTGA	AACTATCTGCCTGCAGGTGTTC	Y
NM_012259	23493	ENSG00000135547	HEY2	6	1647	ACAGAAGTTGGAGCTTTTTAA	CTGTGAATTATATCAAATGATTTTGAAAGA	
NM_025112	79364	ENSG00000070476	ZXDC	3	954	CTGCAGGATCTGCAGTGA	CCCTGTGTCATTGCTAAGTTGACAGA	Y
NM_025195	10221	ENSG00000173334	TRIB1	8	2111	GACAGTGACATTAGTTCCTTCTTCTGCTAA	ATGATCCTCATCATCTCTACTTTTCCACCT	Y
NM_001329	1488	ENSG00000175029	CTBP2	10	1849	CACCCCAACGAGCAATAG	TTAAAATGAGTGAGCAGACAAGGCCA	Y
NM_004789	9355	ENSG00000106689	LHX2	9	636	TCACAAACGACTCTTACCAACCTTTTCTAA	AGCAGCCCCAAGTAGTTTCAATAAGT	Y
NM_001166171	10783	ENSG00000119408	NEK6	9	1673	TGGATGTCCAGCACCTGA	TCAAAATTATTTGGGGAGCTTAAAGAAATC	Y
NM_004959	2516	ENSG00000136931	NR5A1	9	1702	CAAGCCAAGCAGACTTGA	CCCGCCACCCACCTGCCT	
NM_006193	5078	ENSG00000106331	PAX4	7	952	CTACTGTATGGCTTGGAATGA	CTCCCTTTCTCCACATCCTGAGACTT	Y
NM_001489	2649	ENSG00000148200	NR6A1	9	461	AGTGTGGGCAAGGAATGA	TGGAAAAAGTAGGGATGGCCACA	Y
NM_004526	4171	ENSG00000073111	MCM2	3	842	ATGATCCTGCAGCAGTTCTGA	CATTTTTGCTTTCTCTACATCCACAAACAA	Y
NM_078469	56647	ENSG00000107949	BCCIP	10	1628	GGTTGGTCTGTTCCCCCAGTATTAGAATAA	CACCAATCTATATATAGCCTTATTCCACAA	
NM_014390	27044	ENSG00000197157	SND1	7	729	GAATTTGGCTACAGCCGCTAA	CAGACTCTGAAGATCTGCCTTGCTTT	Y
NM_003707	8607	ENSG00000175792	RUVBL1	3	473	CAGCAGGATAAGTACATGAAGTGA	GAGAAAATGTGAACGCAGTAGAATAATGCA	Y
NM_001166135	55131	ENSG00000106344	RBM28	7	594	GCAAAGAGGAGCAAATGGTTTGATAGTTGA	CCCCATTCTTTTCCAAGTGTCTCC	Y
NM_000122	2071	LRG_462	ERCC3	2	486	TTCAAGCGCTTTAGGAAATGA	CTATCTCAGGGTACATCTCTCTGGTAGC	Y
NM_032638	2624	LRG_295	GATA2	3	1777	GTGACCGCCATGGGCTAG	TGGCAAAATCAGACCCAGGC	Y
NM_003069	6594	ENSG00000102038	SMARCA1	X	1988	CCAATGGTAAAATTTTCAGCATTTTCCTAA	ACACCCAGGCCCTTCTGAAC	Y
NM_001242452	3663	ENSG00000128604	IRF5	7	1396	CCAGCTGGCATGCAATAA	TGAGATACTATCTCACCTGTCAGGTTG	Y
NM_002017	2313	ENSG00000151702	FLI1	11	2459	TCACACTTAGGCAGCTACTACTAG	GCCACTAATACCAAGTTTTAATTTTCTCCT	
NM_001145928	79595	ENSG00000136715	SAP130	2	969	TCCAAATTGAAGCGAAAGGAAAAAGTCTAG	TAGACTACCCTCTAAGAGGAACTCTATGCG	Y
NM_006195	5090	ENSG00000167081	PBX3	9	1633	GTGCACTCGGATACCTCTAACTAA	GCAGCTGTTCCTTCCCTCC	Y
NM_002467	4609	ENSG00000136997	MYC	8	656	CTACGGAACTCTTGTGCGTAA	GAACTTAAAGACCTTAAGGCCCCCA	Y
NM_001190799	10733	ENSG00000142731	PLK4	4	861	TTTTCTAATCCGACTCCTAATTTTCATTGA	ACGAGAAATACCTAATGTAGGTGACCG	Y
NM_018078	55132	ENSG00000138709	LARP2	4	1261	GAGTCCAGTGACAATTCACATTAA	CTGTAAATTATGTAGTGCTCAATAAAGTGA	Y
NM_001421	2000	LRG_335	ELF4	X	1981	CTCATTAAGATGGAGCCCCATGACATATAA	GACCTTCCAGCAATGAGTTTCCATG	Y
NM_000539	6010	ENSG00000163914	RHO	3	1806	CAGGTGGCCCCGGCCTAA	GCCTCCCAAAGTGCTGGGATTA	
NM_003658	8538	ENSG00000043039	BARX2	11	1057	GAACCCCCACCATTAAGCTAA	ACTTGGACACCAATGGCCTG	Y
NM_005011	4899	ENSG00000106459	NRF1	7	2081	GTGGTGACATTGGAACAGTGA	CTTTACGGAGGTCCCCAGC	
NM_207426	399823	ENSG00000186766	FLJ46831	10	2401	GAAGGGACCGAAGTTTGA	GATTGTCCAATTGTCCATTGCATTTTCCAA	Y
NM_016024	51634	ENSG00000134597	RBMX2	X	675	GACCGTTGGCGTCACTGA	AGAGGCAATGGTTACACAACACTGT	Y
NM_001143835	4798	ENSG00000170322	NFRKB	11	1130	CAGGCACCTGAGCAACAATGA	AACAAACAAACAAACAAACAAAAAACGCTT	Y
NM_006375	10495	ENSG00000165675	COVA1	X	2111	GAGGGCTTGAAGCTGACCTAA	ACTGGCAGGAGTAACAGAAACAGAC	Y
NM_014602	30849	ENSG00000196455	PIK3R4	3	561	GGGATTGTGAAGGTGTGGAAATAA	ACTGATTCCTTGTGACATGGACTCCA	Y
NM_138693	136259	ENSG00000266265	KLF14	7	673	TTCACCACCTGCCTGTAG	GAAGTATCTCCCAGCCACACATCTACA	
NM_032438	84456	ENSG00000198945	L3MBTL3	6	1876	GCAGAGAAGAATTCTCACAATGAACTTTGA	AATATTTTTGTCATCATCTTGGGACACAGT	Y
NM_001040011	375757	ENSG00000175854	SWI5	9	453	GGGCTGGACATGAATGACTGA	TTATGCTAATTCCTCACAGGATGATTTTAA	
NM_001146003	79858	ENSG00000114670	NEK11	3	910	ACTTTGAAGAGCAGTTGCTGA	TCTCAAGTTAGTGAAAAGATCCAATGAAGG	Y
NM_016542	51765	ENSG00000134602	MST4	X	1964	TCAGCAGACGAATCCCCCTAA	CATTGTAGCTATAATTAATGCTTAATGAAA	
NM_006325	5901	ENSG00000132341	RAN	12	535	GATGAGGATGATGACCTGTGA	TGTACAAAAATAAACATAGGGAGAAATTAA	Y
NM_013355	29941	ENSG00000160447	PKN3	9	494	CGATTCCTGGAACCCTGA	GACCCCAGAACGGAGCTTCAA	Y
NM_002412	4255	ENSG00000170430	MGMT	10	702	CCTGCTGGCCGAAACTGA	GAGGTGCAAGCTCCATCGAAC	Y
NM_004830	9439	ENSG00000112282	CRSP3	6	1121	GTGTCTTTACCAGTAACTCAGTGA	GTTCTTTTTAAAAGAATCTGTAACTCTTAA	Y
NM_016521	51270	ENSG00000183434	TFDP3	X	553	AGTGAGAATGACGAGGATGACTGA	TGTTTATTGTTCTGAAAATAGAATTCTGCC	Y
NM_003565	8408	ENSG00000177169	ULK1	12	1981	ACTGGCATCTGTGCCTGA	AACACATCCAGCTTCTGCCCT	Y
NM_025215	80324	ENSG00000177192	PUS1	12	455	GACGGAGACACTGACTGA	GCCTCCCAAGTGCTGGGATTA	Y
NM_016307	51450	ENSG00000167157	PRRX2	9	500	GTGCCTACGGTGAACTGA	TGGCTGCTGGTCTGAGCC	Y
NM_201634	6932	ENSG00000081059	TCF7	5	2205	ACTATGAATTCACCCTCTGTTTACAGATAA	AGGGGAGATGACAAGTGTAATCCCT	Y
NM_003934	8939	ENSG00000107164	FUBP3	9	1510	CGCCCACAGGAGCAGTAG	AGCTAGACATCTAGACTGTGCACCAA	Y

NM_021619	59335	ENSG00000130711	PRDM12	9	1508	CCGGCCATGGTGCTGTGA	GTGGCCCATCTCACTGAGCT	
NM_001256280	NULL	NULL	ZNF26	12	1267	CTTCGTATACATCGGAAGACTCATAAATGA	TGGGAGGCTGAGGCAAGGA	Y
NM_001113575	51265	ENSG00000006837	CDKL3	5	407	TTGAAGAGAAACAGGTTTTTTTTCTGGTAG	AGAACTCATTTAAAGAGGCCTGTGTGTTAT	Y
NM_003440	7699	ENSG00000196387	ZNF140	12	1201	TCATTCCTTACTGAACACCAGTGA	CTTACCTTCCTGATTAAGGCAAACTAACCC	Y
NM_003337	7320	ENSG00000119048	UBE2B	5	1935	ATTGTTGAACAAAGCTGGAATGATTCATAA	TTTAAACTGGGAAAGGCAAAAACAGGTC	Y
NM_007313	25	ENSG00000097007	ABL1	9	2172	AAGGAAATCAGTGACATAGTGCAGAGGTAG	TGGGGGCAACCTGCCACC	
NM_004052		NULL	BNIP3	10	1004	TCCACCAGCACCTTTTGA	GTGAAACATCAAGGCGGCAG	Y
NM_001191054	647589	ENSG00000227059	ANHX	12	753	GGGAGCAGCCTGGGCTGA	AACTGTCCAGATGTCCATCTGTC	Y
NM_002958	6259	ENSG00000163785	RYK	3	1199	CTGGGGGCCTACGTCTGA	GGAGTCTGAGCCAGCTTAACTGT	Y
NM_003206	6943	LRG_446	TCF21	6	633	GGAACCACCGCGTCCTGA	TCTTCATTATGAAACTCATATGCAATTTTC	Y
NM_002653	5307	ENSG00000069011	PITX1	5	1225	TGCCAGTACAACAGCTGA	GAATGGTGGTGGGGAAGCG	
NM_001185063	7626	ENSG00000186376	ZNF75D	X	1526	GCATGTCTAGTGTCTCCAAACTGA	CATGTGTGCATTTAATCCTCACAGTAGC	Y
NM_001143676	6446	ENSG00000118515	SGK1	6	1202	ACGGACTCTTTCCTCTGA	ACCATCCTATACTCCCCTGGAGTC	Y
NM_004269	9442	ENSG00000160563	CRSP8	9	608	GACACCTGCCGGCAGTAG	ACGCAGGGCAGTACGTGC	Y
NM_006161	4762	ENSG00000181965	NEUROG1	5	875	TGTTTCATTCCTTACCACTAG	AATCAGGTGCGTTTTATTTTTTAATTGTTT	Y
NM_001253882	9442	ENSG00000160563	MED27	9	527	ATTCCAAGAATATTCCATTGGAAAGTCTGA	TCCATCATAAGGTCTCCAAATTGCCT	Y
NM_004441	2047	ENSG00000154928	EPHB1	3	1525	CCAACGGCAATGGCATGA	AGTTTACTACATTCTGCTTCTTGGCATTCA	Y
NM_014468	27287	ENSG00000151650	VENTX	10	1803	ACGGGGGATGCATTTTGA	GGCTGCCCACCTTGTAGG	
NM_001008225	4850	ENSG00000080802	CNOT4	7	1904	CACACTACTGTGGCCTGA	ACTAACCACTTGCTATGGCTTTGTAAGAAG	Y
NM_007344	7270	ENSG00000125482	TTF1	9	545	GGCCGGTGGATCATCTGA	CAGTACCCATATAGCTGAACTCACGTAATG	Y
NM_001159700	2273	ENSG00000022267	FHL1	X	1514	GACTGTGCCAAAAAGCTGTAA	TGTGGTTTTTACAGGTTTGTTTATAATTCC	
NM_001174157	57623	ENSG00000066827	ZFAT	8	874	CCGGCCCAGGAACTCTAG	GTTTCCAAAGCAAACCTAAATGCAGAG	Y
NM_005375	4602	ENSG00000118513	MYB	6	1372	CGGACGCTGGTCATGTGA	TGTTCTTTCTTTTCCATTGTAATGATTCCT	Y
NM_014500	27336	ENSG00000102241	HTATSF1	X	511	AGTAGCGATGATGATGACGATGATATTTAA	TTTATAGAGAGGAAATGGAGACATACGTGC	Y
NM_016267	51442	ENSG00000102243	VGLL1	X	448	CATCGATACCTGCAGCATCTTTAG	CCTGATACTGGTCATGTGATCTTAGGC	Y
NM_001135031	8328	ENSG00000165702	GFI1B	9	827	AGCCAGCACAATCTCAAGTGA	TCTGTGCACGTGGGGGCT	Y
NM_153710	169436	ENSG00000281245	C9orf96	9	855	TCTGGGGGACTGGAATAG	TAGCCGGTGGCTTTCTGG	Y
NM_020385	57109	ENSG00000280706	REXO4	9	1071	TGCAGTGACGACGCCTAG	GCCAGTTTCTGCCTTAATCATTTCTG	Y
NM_005915	4175	ENSG00000076003	MCM6	2	1389	GTTAACCCTAACTACTTGCTCGAAGATTGA	TTTTTTCTTAAGTGAAAGCAAGGCTATTAG	
NM_003413	7547	ENSG00000156925	ZIC3	X	2210	CCTCCTAATTTTAACGAATGGTACGTCTGA	GACAAAGATCAGTAGATAACTTAAATAGAC	
NM_005923	4217	ENSG00000197442	MAP3K5	6	889	ATCATTGACTTTCGAAACAAACAGACTTGA	GAACAACCTGAATGTCAGTTGTCTGACT	Y
NM_052821	11091	ENSG00000196363	WDR5	9	2159	ACAATTAAACTGTGGAAGAGTGACTGCTAA	CCTAAGGTTACTGTAAAAACCCAGGACT	Y
NM_006805	10949	ENSG00000177733	HNRPA0	5	1945	TATGGAGGCAGCTCCTTCTAA	CAAAATGACTACTGAGCAGCTATAACCTAG	Y
NM_004189	8403	ENSG00000168875	SOX14	3	1003	CACGCTACGGCCATGTAA	CAGACCACCAAGCTGGGC	Y
NM_006696	10902	ENSG00000112983	BRD8	5	504	ATTGAAGCAGATATGAAGATGAAAAAGTGA	TCACACTACAGCAAGCAGTTCATAAGAC	Y
NM_016604	51780	ENSG00000120733	JMJD1B	5	1507	GAATCCAAACTGGCAAGGTCCTAG	AACATGGCACAGTCTCAACAAGCAA	Y
NM_001964	1958	ENSG00000120738	EGR1	5	1414	TTTTCTCCCAGGACAATTGAAATTTGCTAA	ATACTCAGTCCTCATAAATAACGAACTCCA	Y
NM_175747	167826	ENSG00000177468	OLIG3	6	1333	TCCAAGGACTTGCTCAAGTGA	AATTTACCCATCCTGTTTACTTGTACGTAA	Y
NM_014279	10439	ENSG00000130558	OLFM1	9	1186	CGCTCCGACGAGTTGTAG	TGGAGATCCAACAGGGACCTGT	Y
NM_006290	7128	ENSG00000118503	TNFAIP3	6	2175	CAGTTCAAGCAGATGTATGGCTAA	TTATTATTTATGAAAAGCTGCCTTGCTACC	
NM_003852	8805	ENSG00000122779	TRIM24	7	816	AGCATTGAAGAACGCCAGTTGCTTAAATAA	TAGACACACAGCAAACCCAAATGTATATAC	
NM_001012415	402381	ENSG00000165643	SOHLH1	9	1117	GCTGGTCCCCCCGCGTAA	CTACCCCAGGGCACCAGG	Y
NM_023067	668	ENSG00000183770	FOXL2	3	1548	TCGCGCCTCGATCTCTGA	ACAAAGCAGCAGCGACAG	Y
NM_001194954	9782	ENSG00000280987	MATR3	5	2462	GAAGAACGCAGACAGAAGAAGGAAACTTAA	AATTCATATTCTTGAATAACATTTCAAATG	
NM_080660	92092	ENSG00000146858	ZC3HAV1L	7	1018	CTCACAGTGTCTTGCGAGAAGTAA	TATCATCCAAGCACAGTATTGCTGAGG	Y
NM_024625	56829	ENSG00000105939	ZC3HAV1	7	874	ATGAAGAGAGGGCCAGAGTAA	TTTATCTTTTTAGTAACTTCCTTTTTCATC	Y
NM_178138	8022	ENSG00000107187	LHX3	9	1255	GTAGACCACGCTCAGTTCTGA	AAGAGAGCCCAAATTCATACTAGGTG	Y
NM_001130992	5947	ENSG00000114115	RBP1	3	1082	CGTGATCTCAGCTCACTGTAA	ACTGCACTCATTCATCCCAGGG	Y
NM_005634	6658	ENSG00000134595	SOX3	X	913	CCGCTGACCCACATCTGA	TTAAAAGGAAACCATTTCAGATTATGCTTG	Y
NM_001168389	10370	ENSG00000164442	CITED2	6	1057	AGCAGAGTGAGCTGTTGA	TGCTCTCCGCTTAAGCTCATCATG	
NM_022750	64761	ENSG00000059378	PARP12	7	991	TTCAGCAGCCGACAGTGA	ACGTCTGCTGTTGATGAGCC	Y
NM_021138	7186	ENSG00000127191	TRAF2	9	894	GTGGACCTGACAGGGCTCTAA	GAGAACACAGCCTGGCTAGCTAG	Y
NM_017747	54882	ENSG00000131503	ANKHD1	5	618	GGAAACATGCATCTCAAATATGTCAACTAA	TAGTCTTAAAAGTGCTTTTTTAAGGCAGTC	Y
NM_001253764	NULL	NULL	SRA1	5	813	TTCCAGCAGGCTTCATAA	TCTCCCTGTGCCTTTTCTGCTT	Y
NM_133174	10307	ENSG00000113108	APBB3	5	497	TCTCTGCTCCATATGCCCTAA	ATGTTTGTTGAATGAAGTCAGATCTCCATC	Y
NM_201999	1998	ENSG00000109381	ELF2	4	1446	ACAGAAGGACTAGTGACATGTGAGAAATAA	CAAAAGGTCTGATTGTGAAACAAATGGTTT	
NM_194249	373863	ENSG00000256453	DND1	5	677	GGTACCATGGTTAAACAGTGA	GGCCTGGATATGAATTTATAATACCCTACA	Y
NM_013446	23608	ENSG00000133606	MKRN1	7	1657	CTGGAAGATTTTTATGACTTGGATCTATAG	GGCTAGTTAAGGTTTCCCTTTCTCTCTC	Y
NM_004333	673	LRG_299	BRAF	7	764	GCGTTTCCTGTCCACTGA	AGCGAGACATCCTTAATGTATTTTAAAAAA	Y
NM_005642	6879	ENSG00000178913	TAF7	5	685	GAGGAGCTAGAATCACTCCTAGAGAAGTAA	TTCTTTACCCCTGCTGTGTTCCTTTTTTTC	Y
NM_024757	79813	ENSG00000181090	EHMT1	9	1353	GCCGCCGACCCCCTATGA	CTGCCTGGAACGTCCCCT	Y

NM_003883	8841	ENSG00000171720	HDAC3	5	772	AATGACAAGGAAAGCGATGTGGAGATTTAA	AATAATAAATGTAGAATACATACACAGGGC	
NM_014245	9616	ENSG00000114125	RNF7	3	1706	GTCCAAAGAATCGGCAAATGA	AGAAAGTTACTAAAACAGTTCACTGCACAA	Y
NM_017444	54108	ENSG00000104472	CHRAC1	8	2086	GACCATGATGAAGCTGACTCCTAA	ATATAAAGGAATGGACTGCTGATACCTGTG	Y
NM_001020825	2908	ENSG00000113580	NR3C1	5	1613	CCAGAAAGCACATCTCACACATTAATCTGA	ATTAGCAGGCGAACATTGCCAC	Y
NM_006734	3097	ENSG00000010818	HIVEP2	6	1820	CCTTCATCAGAAAAGAGTCAGCTACATTGA	GACTGTCTTCTCACTTGTTAAAAGCATTCA	Y
NM_003461	7791	ENSG00000159840	ZYX	7	611	GCTAGAGCCCAGACCTGA	AGTAAAAGGATGATCATGGGAGGGA	Y
NM_001080952	5325	ENSG00000118495	PLAGL1	6	1304	CTGCCTCATTTCCATCATGCATTCAGATAA	TCAAAACAGTGGTTTCTGTGGGG	
NM_201589	389692	ENSG00000182759	MAFA	8	1465	GCCGACTTCTTCCTGTAG	GCTGCTCAGTTTCTCCCTGGT	
NM_015117	23144	ENSG00000014164	ZC3H3	8	581	CACATCAAACCACGTCTGTGA	CCCCACCCCAGATGGTGC	Y
NM_001261843	9831	ENSG00000276340	ZNF623	8	2427	GATAAGGGGGAACACACAGGTAACTTATAA	ACACATGTGCAAATACATACTTTTACACAA	Y
NM_173831	NULL	NULL	ZNF707	8	1035	AGGCACGGGGAGGTGTAG	CAATGGCGCAACCTTGGCT	Y
NM_178564	340371	ENSG00000185189	NRBP2	8	2257	CGTGGGACCCAGGCCTGA	GAGACTGAGGCAGGTGACC	
NM_032272	84232	ENSG00000179632	MAF1	8	683	GTCCCAGTGATCTGTATTTGA	CTCTGCCTCCATTTCAGCAACT	Y
NM_006472	10628	ENSG00000265972	TXNIP	1	1590	CTCAACAACAATGTGCAGTGA	GCCCCCACTCTGCCGACA	Y
NM_005526	3297	ENSG00000185122	HSF1	8	572	GACCCCACTGTCTCCTAG	CTGGGGAGTCGGGCAGGC	Y
NM_006099	10401	ENSG00000131788	PIAS3	1	1104	GACATCATTTCCCTGGACTGA	CACAGAACACCCCCACCGT	Y
NM_006706	10915	ENSG00000113649	TCERG1	5	1044	CCCACGAGACGATCAACAAAATAA	TGCATTATTATATTCAAACCTTTAAAAACT	
NM_138367	90987	ENSG00000198169	ZNF251	8	915	AAGAAGATTTTCCAAGAAAGACATTTTTAA	AGAAAACAAACCAGTGGTTTCCAC	Y
NM_006958	7564	ENSG00000170631	ZNF16	8	560	CAGTTGATTCACACCAGGGAATAG	AACAGGAGGGACAAAGAAGGG	Y
NM_001003688	4086	ENSG00000170365	SMAD1	4	1419	CCTCATAATCCTATTTCATCTGTATCTTAA	TCCGGTCTCTTCATGCTGCT	Y
NM_004284	9557	ENSG00000131778	CHD1L	1	437	TCAAGACAGCTGGTGCCTTAA	CATGAACTGGCTGAAGGTGCTTC	Y
NM_001185081	2332	ENSG00000102081	FMR1	X	2477	GTCAGCAACCACTCGTGA	ATTATTTTCAAGTGTCTTCGTTTTTGCCTT	Y
NM_007080	11157	ENSG00000164167	LSM6	4	585	AGTACACAGAAGAGACGGATGTGA	ACTCTTGGCTTTCCAATTCTGTTTTTTATT	Y
NM_004575	5458	ENSG00000151615	POU4F2	4	1843	AAAAGAATGAAATATTCCGCCGGCATTTAG	ATACTTTACCATAGAAACAGCCCTTACTAA	Y
NM_000024	154	ENSG00000169252	ADRB2	5	741	AATTGTAGTACAAATGACTCACTGCTGTAA	CTCAAAGGCAAATGAAAGGGTTAAGCAATC	
NM_001203247	2146	LRG_531	EZH2	7	454	GAAAGAGAAATGGAAATCCCTTGA	CAGTGTTCTTCATATATGTCTTCCACATAT	
NM_003071	6596	ENSG00000071794	HLTF	3	2283	AATGAAATCAGAACATTAATTGACTTATAA	AATTGAGCAAAATAAAGTTAGTTAGGATAT	
NM_001001661	155054	ENSG00000204947	ZNF425	7	1006	GAGAAGCCCTCCAGCCTCTAG	CCCTAATGTTAAGTATAGTTCTTTGAGAAC	Y
NM_001892	1452	ENSG00000113712	CSNK1A1	5	1751	TTTTGTTTTGATTTTTTACCAGGTTTCTAA	TCTTCAGTATTACGGTACTAATATCCCTTA	
NM_003575	8427	ENSG00000170265	ZNF282	7	1780	CCTCCTGAGCGAGACTAG	CACTACCCCCCTCCCCCA	Y
NM_012256	7988	ENSG00000170260	ZNF212	7	1374	CCCAATGGCCTGCTTTAA	CACATCTTGTCTTCTGTCCTAGGTTTAAGT	Y
NM_001163474	155061	ENSG00000181220	ZNF746	7	1771	GATGGCGGGGACATGTGA	TAGATCTCAAGGTCAGTTACCTGTCTG	Y
NM_014983	22993	ENSG00000113716	HMGXB3	5	1131	GCCGCAGTGGCAGAATAA	TCAGCCTCTGGTTCTTTGTGC	Y
NM_207336	168544	ENSG00000181444	ZNF467	7	530	CCCCCGCTCTTCTTCTGA	GGAGAACCAATTAACAGCAGCTG	Y
NM_002609	5159	ENSG00000113721	PDGFRB	5	2104	GCAGAGGATAGCTTCCTGTAG	GGGAGAGCGAAAATCCCCACTTTA	Y
NM_001804	1044	ENSG00000113722	CDX1	5	1058	GTGAAAGAGGAGTTTCTGCCATAG	GCTTACAAAATGGACTCACATTTTTACGTA	
NM_015093	23118	ENSG00000055208	MAP3K7IP2	6	2072	GAGATGCCAAGGCATTTCTGA	TAAAAACAGTCCAAGAAATGCAGCTGAGTA	Y
NM_005850	10262	ENSG00000143368	SF3B4	1	405	GGCCCTCTCCCTCAGTAA	CAACTGCAACAGCACATTACAGAATAAG	Y
NM_001099696	29803	ENSG00000214022	REPIN1	7	1279	CAGAAGAAGCACGATGTCTGA	GAACTGAGGCAGGGGGCC	Y
NM_018047	55696	ENSG00000086589	RBM22	5	1141	AAACACAGCAGCCCCTAG	TAAATCATCACGGAATGGATATAATCAAAA	Y
NM_173680	285971	ENSG00000196456	ZNF775	7	716	AAGGAGGAGGCGCGCTAG	TACCTCCTCAGGCAGCCAA	Y
NM_014779	9819	ENSG00000196428	TSC22D2	3	1372	CCGAATGTCTCCTCAGCATAA	TAAATACTCAGTATACACTGCACTTAATCT	
NM_001172831	91975	ENSG00000145908	ZNF300	5	1212	CAGAGAATTCACACAGTGGTAAAATCATAA	ATGACAGATAGTAGGTGTTATCTTAGAGGC	Y
NM_004698	9129	ENSG00000117360	PRPF3	1	429	AGTGAATCTGTGTTAGAGTCCACTGATTGA	TCCACTAGACTTCAAAAATACACATCCATC	Y
NM_005067	6478	ENSG00000181788	SIAH2	3	1198	AATGTTACTATTTCTACATGTTGTCCATGA	CCGTGCCATGACCTTGGC	Y
NM_178427	405	ENSG00000143437	ARNT	1	2496	CTAACTATGTTTCCCCCCTTTTCAGAATAG	AGCCAAGATCGTGCCACTG	
NM_001145415	9869	ENSG00000143379	SETDB1	1	560	GAATGCAGAGGACGTCTTCTTTAG	CGAGAACTCCATCTAAAAAAATTCACATGC	Y
NM_181746	29956	ENSG00000143418	LASS2	1	1155	CTCAATAACAACCATCGTAAGAATGACTGA	CCAGCTAGATGTATTTTTAGTAGAGATGGG	Y
NM_001271088	6944	ENSG00000163159	VPS72	1	750	AAAAGTCCCTGTTCGTGA	ATTAACAGCCATGGCATCTCTTCCA	Y
NM_005614	6009	ENSG00000106615	RHEB	7	1287	TCTTCATGCTCGGTGATGTGA	ACAGAGTGAGACCCTGTCTTTAAAAATGTA	Y
NM_020832	57592	ENSG00000143373	ZNF687	1	876	GCTGTTGGGGACAACTAG	CCAAGATACAAAAGGCACTACATAGAAGTT	Y
NM_000449	5993	LRG_101	RFX5	1	1726	AAAGCAACACCCCCATGA	TAAAAAACAAGCAAAATTAAACACATTTTT	
NM_145796	23126	ENSG00000143442	POGZ	1	2258	GAAGCTGACCTAGATCTGATGGAGATTTGA	GGCAGCAAATTCAAAGTACAGAAATTCAGT	
NM_001083963	11022	ENSG00000182134	TDRKH	1	1153	GATAACCTTGAAGATGACTACTTACTCTGA	GGTTATTTCTGAAGTGACTTTAGAGAAAGA	Y
NM_005060	6097	ENSG00000143365	RORC	1	1581	GTGGGGCTGTCCAAGTGA	GCAGGGGTCGGGTGAGGG	Y
NM_170606	58508	ENSG00000055609	MLL3	7	2087	CGGAAGTGGATGAACTGA	AACTTATTTGAAAATCTAAGAAAACCAAGT	
NM_004688	9111	ENSG00000123609	NMI	2	405	CAACCTCACATAGCATACTTTGAAGAATAG	GGAAAATGTTAAGAGTCAAGTCTGCATATT	Y
NM_005334	3054	ENSG00000172534	HCFC1	X	1970	AAATCTAAGGCCGATGGTCAGTGA	GGCCACAGCAGAGGCCTC	
NM_001025243	3654	ENSG00000184216	IRAK1	X	1533	GAAAGTGATGAATTTCAGAGCTGA	GACTACATCAGGAAAAACCTGACACG	Y
NM_001456	2316	ENSG00000196924	FLNA	X	497	CGCGTTGTGGTGCCCTGA	GTGCCTTCTGGAGTCAGGGT	Y
NM_000117	2010	ENSG00000102119	EMD	X	506	GAAGAAGGCAACCCCTTCTAG	CACAAACATCTCTGGGTAACATTTGATTCC	Y

NM_004515	3608	ENSG00000143621	ILF2	1	788	GAAAGCATGGAAACTCAGGAGTGA	CATGCCTGGTTACATGGGCCT	Y
NM_000906	4881	ENSG00000169418	NPR1	1	758	AGTAGCACCCGAGGCTGA	AGCCTGCATTCCCAGCTTAAGAAT	Y
NM_004821	9421	ENSG00000113196	HAND1	5	1014	CTGGAGTTAAACCAGTGA	GAAATACATTCTTTATCTGCCGGCTTCC	Y
NM_001255981	148327	ENSG00000143578	CREB3L4	1	479	CTGCATGCAGATGAGATGTGA	GGTTTCCTTCCCTTCTGTAACTTGGA	Y
NM_001363	1736	LRG_55	DKC1	X	1002	GCAAAAGAGGTAGAATTGGTTTCTGAGTAG	CCCTCTCCTTCTCTGTTCTTCTAGAGAT	Y
NM_001098475	126668	ENSG00000163239	TDRD10	1	770	CAGCAGGCTGCTGCCTAG	CCCTACACTTCAAACATGTATCTATATCCC	Y
NM_182499	126668	ENSG00000163239	TDRD10	1	617	CACATCCTAAAGTTTGAAGAGTCTAAATAA	CCCTACACTTCAAACATGTATCTATATCCC	Y
NM_003264	7097	ENSG00000137462	TLR2	4	1008	GTAAATCTGAGAGCTGCGATAAAGTCCTAG	ACTGAGTATTTATTTTGTTAAATTACAGAA	Y
NM_007349	22976	ENSG00000157212	PAXIP1	7	639	CTCAACAAGCCTACATATAAGTTTAACTGA	ACAAAAAAAATGGCTGGCTTCTAGAAATTA	Y
NM_020524	57326	ENSG00000163346	PBXIP1	1	1121	CACCACCACCGGGGCTGA	GAGCAAAATGGAGCCAGGGT	Y
NM_001252406	51043	ENSG00000160685	ZBTB7B	1	2020	GGTGCCATGGAGTCCTCTTAA	ACCAGCCCTGAGCCCCAC	Y
NM_003993	1196	ENSG00000176444	CLK2	1	530	CGGGATATCAGTCGGTGA	ATATGATAAGGTGGACAGGGGAAATAAACT	Y
NM_001427	2020	ENSG00000164778	EN2	7	2324	AAGTCGGACAGCGAGTAG	TAATAATCACCTCTCGCTACAAAGGCA	Y
NM_001201564	5356	ENSG00000171566	PLRG1	4	1868	CCAGAAATTATCAAGAGAAAGAGATTTTAA	AACTTGAACACAATAGTTGAAGCTTTGCCA	Y
NM_144979	166863	ENSG00000151962	MGC27016	4	926	AATCAGGCCTCCTTCTTCTGA	AACAACTTTCTTCAACATCCTTCACTTGAG	Y
NM_170708	4000	LRG_254	LMNA	1	1163	CAGAACTGCAGCATCATGTAA	CAATGAGCAGGAGGATGCAGTGA	Y
NM_001199653	11243	ENSG00000160783	PMF1	1	670	AGCTGGTTGCTGTGCTGA	GGTTTTTGTACAAGGGGCTCTGC	Y
NM_001199654	11243	ENSG00000160783	PMF1	1	656	CTGAGGGAGCCTGAGTGA	GGTTTTTGTACAAGGGGCTCTGC	Y
NM_015508	25976	ENSG00000163659	TIPARP	3	1817	GAAGAAGTCAGTAACACTGTTTCCATTTGA	CACACTGCAGAAAAGTTTAAGCAGCAA	Y
NM_001100816	9443	ENSG00000155868	MED7	5	470	CTAATTGATGAGATGAATGAAAGACCATGA	TTATGCTACTTTTTGGAGCTTAAGCAAAAT	Y
NM_006617	10763	ENSG00000132688	NES	1	738	TCCTCAGGGGAGGACTAG	CATCCCCCCAGGTCCTCCT	Y
NM_004494	3068	ENSG00000143321	HDGF	1	1522	CTCCCTCACAGCCTGTAG	CAAGAAGTTTGGATTTCCAAATGCCTAAAA	Y
NM_007017	11063	ENSG00000039600	SOX30	5	1104	CAAAACATGAGGGTATCTTTTCAACTTTAA	TGTTTAAGTCTCGTGTGAGCTAATAGAATC	Y
NM_005240	2117	ENSG00000117036	ETV3	1	1076	AAGATACAAACTCTTTTGGTAGGGAATTAA	GAAAACAGTTAAATACAATCTACAGTAGTC	
NM_006186	4929	ENSG00000153234	NR4A2	2	1492	AAACTTTTCCTGGACACTTTACCTTTCTAA	TTCTGGTTCACCTGATTTCTTTTGCCA	Y
NM_173491	134353	ENSG00000155858	LSM11	5	1654	CTGGTTCATCTTGCACAGTGA	GCTGAGATTACTTTTTTATGTAGCACCTGC	Y
NM_002432	4332	ENSG00000163563	MNDA	1	423	AACAAGGAAGGACCAATGAATGTTAATTGA	AATGTGGATAAGGCCCCATGTGT	Y
NM_198930	149628	ENSG00000163564	PYHIN1	1	512	AAGGTCACCAAGGACAAGGATATCAAATAA	GCAGGGCACAGTCAGTTCAGT	Y
NM_005531	3428	ENSG00000163565	IFI16	1	434	ATGGAAACTTCACCAGACTTTTTCTTCTAA	CTTGATCAGGGCTTTACCCGGTAAA	Y
NM_003564	8407	ENSG00000158710	TAGLN2	1	867	CCACGCCAGATCCTCTGA	GAGAATGAGGATGGGGAGGAAACTCA	Y
NM_013450	29994	ENSG00000123636	BAZ2B	2	1466	AAGTGGACAGATACTTTCAAAGTGAGCTGA	ACTTAAACTCACCCTCTTCCACCCA	Y
NM_005598	4807	ENSG00000171786	NHLH1	1	1895	CACGTGCTGGACGTCTGA	TGTGGCCCCTTTTCTCTGTCTT	
NM_207005	7391	ENSG00000158773	USF1	1	849	GTCATCAAGAATGACAGCAACTAA	CACACCGGTCTGGCCCAG	Y
NM_006724	4216	ENSG00000085511	MAP3K4	6	695	CAGGTTTGCACAGATGAAGAATGA	AGGCGATCCTAAAGAGACGC	Y
NM_006593	10716	ENSG00000136535	TBR1	2	1015	TATGGCTTCTACTCGCACAGCTAG	TGGCACTTTGGGGAAAAACAAATAAG	Y
NM_004460	2191	ENSG00000078098	FAP	2	469	CTAAAGCAGTGTTTCTCTTTGTCAGACTAA	GAGTTCTCATGACAGAAATCCAAAACCACT	Y
NM_177398	4009	ENSG00000162761	LMX1A	1	2193	TCCATGCAGAATTCTTACTTCACATCTTGA	TTGAAGACCCTTTTCTCCTTATGTTTTGCT	Y
NM_006917	6258	ENSG00000143171	RXRG	1	521	CCGCTGCAGATCACCTGA	GCAGCAAAGCCCAGCTTAGATTC	
NM_003181	6862	ENSG00000164458	T	6	837	TCGCCACCTTCCATGTGA	TCACTTCCAGTTTTGCAACTAATTAGATAC	Y
NM_053053	117143	ENSG00000152382	TADA1L	1	1235	GGGCTTTTGCTGTGCTAA	AAATGGCATACCTTAGTTGGAATCTGCTTT	Y
NM_003851	8804	ENSG00000143162	CREG1	1	1464	TAATTCTTCTCTGTTCCCCTTTCTAGGTGA	GCTTTTTGCCAATTAGAAAGAGTGGTATTA	Y
NM_001017977	55827	ENSG00000143164	IQWD1	1	590	CAAGAGAATGAAAATGAGGATGAGGAATAA	GAAGGAAGTCCTTCTATGTGATTCTATCCC	Y
NM_005149	9095	ENSG00000143178	TBX19	1	1604	TCTCCTTCCTCACTGGATGGTTAA	TGCCTCAGATGAGAAGCCCG	Y
NM_004991	2122	ENSG00000085276	MDS1	3	1590	ATCCAGTCCATAAGCCACGTATGA	GAACTATCATTTATGCTATCTCAAAACAAA	Y
NM_013233	27347	ENSG00000198648	STK39	2	1656	GCTCAGTTGAGTGTCAGCTGA	CAGTTCTCTGTACTGGATGCATGATG	
NM_144769	2299	ENSG00000168269	FOXI1	5	1294	GAGGGCACCGAGGTCTAG	AGAGGTGAAAAGAGTCTTGATATGCATTCT	Y
NM_181093	57147	ENSG00000000457	SCYL3	1	819	GAGCTGAACTGGGAAGATAATAACTGGTGA	TGGAAATGGAGGCGCTCCAA	Y
NM_005618	28514	ENSG00000198719	DLL1	6	848	GTGGCCCCCCAGGTGTAA	AGGAGAGGAACGGGCTGCT	Y
NM_003142	6741	ENSG00000138385	SSB	2	485	AATGGTGCTGGAGACCAGTAG	GTGTTACCAACTTTCCTGTGTAAATATACA	Y
NM_021025	30012	ENSG00000164438	TLX3	5	715	GTCACCTCCCTGGTGTGA	GGTTGCAGCCGAGGCGAA	Y
NM_001161562	23043	ENSG00000154310	TNIK	3	1558	CTCAACAGAAATTCCATGATGAACTGGTAA	GAGATAACACTGCTGTGATGAACAGTGAAA	Y
NM_001037738	4869	LRG_458	NPM1	5	502	GTTTAATTGCAGGCGCATTGA	TGCATTTTTAAAAGATTGAAAAGGTTTTCA	Y
NM_001172085	6908	ENSG00000112592	TBP	6	785	ATTCTAAAGGGATTCAGGAAGACGACGTAA	AATATATGGAAGGTTCTCAGATCATTAATC	Y
NM_001083615	140469	ENSG00000071909	MYO3B	2	2223	TCAAAAGGAGACTCTTTTGCTCAACATTAA	ACTGTGACTAGCCCAAGAATAATGTCTACA	Y
NM_004387	1482	ENSG00000183072	NKX2-5	5	645	GGTATCCGAGCCTGGTAG	AACCAGTATGGTTCCAGCAAGG	Y
NM_001038493	1745	ENSG00000144355	DLX1	2	1774	GGCGGCTCTGGAGGGTAG	CTGACGATACCCTGGTGTTTCTG	
NM_004405	1746	ENSG00000115844	DLX2	2	1285	GCGGGGACGATTTTCTAA	GCCTGTCCTCTCCTTGCC	Y
NM_001122770	84614	ENSG00000185278	ZBTB37	1	692	ACCACTGGGCCAGACTGA	TTGCCAAGTGCTTCAGGTGATGT	Y
NM_016653	51776	ENSG00000091436	ZAK	2	1439	CGTGGATGGAGAAACTTTTGA	TGTACTCTGATGACCAGCTTCTTTTAAAAA	
NM_002449	4488	ENSG00000120149	MSX2	5	1496	TATGGCATGTACCACCTGTCCTAA	CCCCACCACCACCAATCAC	
NM_002129	3148	ENSG00000164104	HMGB2	4	884	GAGGAGGAAGAGGATGAAGATGAAGAATAA	CCAGGGAGGCTGAGGCAG	

NM_021973	9464	ENSG00000164107	HAND2	4	956	CTGGAGCTCAAGCAGTGA	GGAAGGAGACACCACACCGA	Y
NM_004882	9541	ENSG00000138433	CIR	2	527	ACACATACTAAAGTGACACAAAGAGAATGA	AGAACAAAACACCTAAAGGGGGCTTAAAAG	Y
NM_001256094	1386	ENSG00000115966	ATF2	2	623	TCCTCTAACAGGCCAATTGTGTAA	ATGGGCCTGGTACAGGGCTA	Y
NM_016290	51720	ENSG00000087206	UIMC1	5	457	AGAGGAAGAAGGAGAAAATTCTGA	GGAATTGTGCAGTACACATTAAGTGAATAT	Y
NM_002011	2264	ENSG00000160867	FGFR4	5	629	TCTGGGGTGCAGACATGA	CACACTGGCTCCTGGGGC	Y
NM_001004106	2870	ENSG00000198055	GRK6	5	1239	CTCCCCACCCGCCTCTAG	TTCCCTCCAGGCCCACCT	Y
NM_000523	3239	ENSG00000128714	HOXD13	2	1388	AAGCTCAAAGATACTGTCTCCTGA	AGGTTCCAGAAACCAGAATTAGTGTTGA	Y
NM_021192	3237	ENSG00000128713	HOXD11	2	626	CAGTATTTCACTGGAAACCCCTTATTTTGA	TCGATTTTCAGTTGCATGGGTTCTG	Y
NM_002148	3236	LRG_246	HOXD10	2	891	GCCAACCTCACGTTTTCTTAG	CCTCCAGCTTTTCTCCCCCAT	
NM_014213	3235	ENSG00000128709	HOXD9	2	922	AAATGCCCCAAAGGAGACTGA	GAGACACATCAGAGAGATCTGTCAGGT	Y
NM_019558	3234	ENSG00000175879	HOXD8	2	1263	GCCGAAGGCCTGACAAATTAA	TTTTTTTTTTTTAAACAGCGCGGAATGTGT	Y
NM_014621	3233	ENSG00000278500	HOXD4	2	459	ACGGACCTGACGACCTTATAG	ACAGGGGTGGCCAACCTT	Y
NM_006898	3232	ENSG00000128652	HOXD3	2	1004	CCCAAACTGACGCATCTGTAG	CCCGAGAACCAATTTATGCACTAGACT	Y
NM_024501	3231	ENSG00000128645	HOXD1	2	945	TCCCAAGAGCCTTCGTGA	AACAGAGCAAGACTCCGTGTCAAAAAAAAA	
NM_031266	3182	ENSG00000197451	HNRPAB	5	740	CAGAATAACTACAAGCCATACTGA	CTCAGCCCTAGGCAGGGC	Y
NM_020666	57396	ENSG00000113240	CLK4	5	1133	CCTTTCTTTGACTTATTAAAAAAGAAATGA	TGGCAAGGCTGGTCTCAAACT	
NM_001145413	4780	ENSG00000116044	NFE2L2	2	662	AGTAAGAAGCCAGATGTTAAGAAAAACTAG	TATTTCTCTGTAACCCTGGTACTAGAAATG	
NM_005649	6940	ENSG00000169131	ZNF354A	5	719	TATAAAATTCATATCGAAGAGGACCCCTAG	AAATTATTGCCTGTTTTTCTATCTAATACA	Y
NM_030613	80108	ENSG00000198939	ZFP2	5	690	CTTACACGACATCAAAGAACTCATACGTGA	AATGCAGGGGAAGATTAGGGG	Y
NM_001178090	285676	ENSG00000178187	ZNF454	5	424	CATCAGAGACATCATATTGGAGAGAAGTGA	CTGCCTCCCGGGTTCATG	Y
NM_001136116	345462	ENSG00000234284	ZNF879	5	927	CTTACTAATCATCAAAGGACTCATAATTGA	TGACCTGAGTTCTACATTGTTCCTGG	Y
NM_014594	30832	ENSG00000177932	ZNF354C	5	773	TTTAAAGGAGATAAAGCCTATGAGGTTTAG	AATCCGCCTTCTACGGTACTGTAATAGTAC	Y
NM_001257293	3187	ENSG00000169045	HNRNPH1	5	1548	TCCAGTGATTTTCAATCAAACATTGCATAG	ACGAGTTTACCTTCAGTTGCACTG	Y
NM_016331	51193	ENSG00000121864	ZNF639	3	1290	CCAGTCCATGAGACAACTTGA	TCCTTCCCCCACAACCCTC	Y
NM_001136001	27	ENSG00000143322	ABL2	1	404	AATCAGACGTGCATAACATTGTGA	GGCTCTCCCAAGCTCCTCA	Y
NM_178042	86	ENSG00000136518	ACTL6A	3	575	TGTGTAGAAAGAAAATGCCCTTGA	GAATTTGAATAATAGTTTTACCTCTGTTGT	Y
NM_133379	7273	LRG_391	TTN	2	445	TTTGCAGCACTCACCTTAACTTAA	TGCCCTTACCAATTTGTCTGCGT	Y
NM_182925	2324	ENSG00000037280	FLT4	5	1866	GTGACTTTCTTCACAGACAACAGCTACTAA	CGAGATCGCGCCACTGAACT	Y
NM_002826	5768	ENSG00000116260	QSCN6	1	1177	GGCCACCCTGCAGCCTGA	CAGACAGTTCTATGGGCAGTCTGT	Y
NM_033343	89884	ENSG00000121454	LHX4	1	654	CTCGATGAAATGGATCATCCTCCTTTTTAA	TCATATTCTCTGGTTTGTAGGAAAGAATTC	Y
NM_152283	643836	ENSG00000196670	ZFP62	5	1361	ATGAGGATGCCTCTGTAG	GCACCTTCTAACTTCTGGTGAAACCA	Y
NM_003106	6657	LRG_719	SOX2	3	1301	CCCCTCTCACACATGTGA	TTTTGCAGTTAACATTAGCACATGATGCTG	Y
NM_001009992	127665	ENSG00000179930	ZNF648	1	1914	TCCTCCTCTGACGAGTGA	TATGGTTAACAAACAGCGCAGGTGA	Y
NM_002500	4760	ENSG00000162992	NEUROD1	2	1864	CTCAATGCCATATTTCATGATTAG	TTCTGGGACAGTACTAGTTTGCAAGATAAA	
NM_004443	2049	ENSG00000182580	EPHB3	3	966	CTGCCTGTGCAGGTCTGA	GCCAAGCACAAAGGGGAAACTG	Y
NM_007212	6045	ENSG00000121481	RNF2	1	2487	CCTACAAAGGAGCACAAATGA	ACATACATACAATATTATTCAGCCATAAAT	
NM_002199	3660	ENSG00000168310	IRF2	4	1216	CGCGTCAAGAGCTGTTAA	AACTGAAGCCTCACAGTAAGAACCT	Y
NM_001007225	10644	ENSG00000073792	IGF2BP2	3	1972	TCACAGCGCAGCAAGTGA	GACTTTCCTGGGCAAAACCAGCT	
NM_024629	79682	ENSG00000151725	MLF1IP	4	1403	AAGCTCCTTGACCAGGGATGA	CCGTCTCAGCCTCCCCAGTA	
NM_001967	1974	ENSG00000156976	EIF4A2	3	807	ATGAATGTGGCTGACCTTATTTAA	GCAGATGGTGCTGATGAACATTTG	Y
NM_001706	604	ENSG00000113916	BCL6	3	1261	CTCCCCAAAGCCTGCTGA	ACCTAGCCTCATCTCAAAAGACGC	
NM_174900	132625	ENSG00000179059	ZFP42	4	1485	ACGAACAAGAATGAACAAGAGGGAAAGTAG	ATCCAACCACACGTAAGCCCAA	Y
NM_001114982	8626	ENSG00000073282	TP63	3	1497	AACCGATCAGTGTACCCATAG	CAGAAGAAATGTACGTAGGAACAAAGTGAT	Y
NM_007315	6772	LRG_111	STAT1	2	1847	TTTGTCTTTTTACAGATGAACACAGTATAG	TAGGGCAGGTAAGGACAACATTTAGGAAAA	
NM_001243835	6775	ENSG00000138378	STAT4	2	444	TAGATGAAGTCTCCTTATTCTGCTGAATGA	TGGCTTTTCCCTCACGCCTT	Y
NM_004600	6738	ENSG00000116747	TROVE2	1	1434	ATTCGAAATTTCACATTAGATATGATTTAA	ACTACAGTTTGACTTTTCACATAACAGCGA	Y
NM_005524	3280	ENSG00000114315	HES1	3	559	AGGCCGTGGCGGAACTGA	TAGTTCATGGAGGATTGGTGAAAAGTTTGA	Y
NM_033030	66037	ENSG00000152430	BOLL	2	1856	TAACAGACAGTGTGGAGCATTCATTATTAA	AGTAGCTTCTCAAAATAAAGCTAAATTCTA	Y
NM_012482	23528	ENSG00000162702	ZNF281	1	906	ACCAGCCAGAGTTACAGGTAA	TCAGTTAATCATATAATTTCAACTTGAGTT	Y
NM_004078	1465	ENSG00000159176	CSRP1	1	1363	TTGGTCCACTCTGAGTGA	TTGAGTCAGAATTTCACCATGTTAGCCA	Y
NM_001207067	9689	ENSG00000082153	BZW1	2	1848	TCTGAAGCTGAAGAAGGTGACTGA	TTTAGCTACACCAGAATTCTGTGTAGGTGG	
NM_004071	1195	ENSG00000013441	CLK1	2	477	TTCTTTGACCTTCTGAAGAAAAGTATATAG	ACTCTTCTCGGCTAATCTTAAGTTCTGTGT	Y
NM_006190	4999	ENSG00000115942	ORC2L	2	1310	AAGGAAGAAGAGGAGGCTTGA	CCCAAAGTGTTGTGATTACAGGCATAA	Y
NM_001114309	1999	ENSG00000163435	ELF3	1	2044	GTTCTCCAGAGTCGGAACTGA	TGTAAGTAAAAGCCTTTTTCCAAATGGCTC	Y
NM_018571	55437	ENSG00000082146	ALS2CR2	2	856	GATGAAAAAGACTCATACTGGGAATTCTAG	CAGAATGCCTTCTGCCTCCATTTAATCAAA	Y
NM_006618	10765	ENSG00000117139	JARID1B	1	1812	GCACCAAGCCGAAAGTAA	AGGGGATTCTAAGAATTTGAATACTAACAA	Y
NM_002479	4656	ENSG00000122180	MYOG	1	961	GATGAAACCATGCCCAACTGA	AAGAGCAGGGGTCCCCAG	Y
NM_014827	NULL	NULL	ZC3H11A	1	1909	CTTGAGCTATCAGAAATGATTGATAGCTGA	TGGCAATATATCCAGATGAAAGGTTTCTAA	
NM_003094	6635	ENSG00000182004	SNRPE	1	1400	ATTACTCTGCTACAAAGTGTCTCCAACTAG	CTGCAGGGAGGTCTTTGCTCT	Y
NM_005686	9580	ENSG00000143842	SOX13	1	1789	GTGGTGCTCACAGACTGA	GGATTCCCTTGCATTAAGCCAATTCC	Y
NM_018208	55224	ENSG00000143845	ETNK2	1	1302	TTGGAGATGCCAAAGTGA	CGGAGCTAGGTGCACAGGA	Y

NM_030952	81788	ENSG00000163545	NUAK2	1	1567	TGCTCAAAGCTCACCTGA	GAGCTACTGAGCTCACGTTTGTTTTGATTC	Y
NM_021795	2005	ENSG00000158711	ELK4	1	740	TTAGAAAGGTTATGTGTGACTGTCATGTGA	GATGTTTTATCCTTGAAACAGCACACAAAT	Y
NM_014002	9641	ENSG00000263528	IKBKE	1	925	GCACCTCCTGATGTCTGA	TTTAGGGGAAAGGCAGAAATCAGGA	Y
NM_003582	8444	ENSG00000143479	DYRK3	1	412	TGCAGTGTATTGCCAAAACTGATTAGCTAG	CCAAATTACCCCTCCAGAACAGATG	Y
NM_004759	9261	ENSG00000162889	MAPKAPK2	1	2374	TGGCTGACCAGGTTGTGA	AATGAGGTGCTGGCTCCTG	
NM_005896	3417	LRG_610	IDH1	2	1030	AAGATCAAACTAGCTCAGGCCAAACTTTAA	TTCCTTTCACTGCAGGATCTACATCTTACT	Y
NM_020439	57172	ENSG00000008118	CAMK1G	1	1244	ACTGGAGTCTGTCTCATTATGTGA	CTGTATACTCAGCTATATGCACATCTGTAT	Y
NM_001122834	55733	ENSG00000280680	HHAT	1	2097	ACCTACGCCACGGACTAA	GCACCACCATTCAGAGTAACTACAAGATTT	Y
NM_001136225	55758	ENSG00000117625	RCOR3	1	499	TGTTTGTGTATGGAATTTGAGCTAATATGA	GTGATGGACCCAGTGTCCGA	Y
NM_002497	4751	ENSG00000117650	NEK2	1	834	ATCCTGGGCATGCGCTAG	ATGGGAGGTAAAGCGCCATACA	Y
NM_001206488	467	ENSG00000162772	ATF3	1	1402	CAGATAAAAGAAGGAACATTGCAGAGCTAA	ACTATCCCATCTAGTGATGCCCCAAA	Y
NM_016343	1063	ENSG00000117724	CENPF	1	957	GAGAACTGTAAGGTCCAGTGA	ATTGTGTCTTTTAATACAACTAAAGATGCT	Y
NM_021141	7520	ENSG00000079246	XRCC5	2	1271	TGTTCTTGTTCACAGTTGGACATGATATAG	TCCTAGAAGCCCAAAGTAAAGCACT	Y
NM_001105537	7701	ENSG00000115568	ZNF142	2	602	ACTGGACCTGAGGGCTGA	GCAGACCTAGGCCTGTTTCAGTATCAAT	Y
NM_015690	27148	ENSG00000163482	STK36	2	888	CCAGCCCATAGCATGTGA	CAACCTAATTGACCGCTCTGTTGAATCTTA	Y
NM_024782	79840	LRG_90	NHEJ1	2	1237	AAGCCAAGGGGTCTCTTCAGTTAA	GACCTTTGATAAGTCACTTACTCACTTCAG	Y
NM_001008910	8576	ENSG00000115661	STK16	2	1958	CAACATACTACCCAAATCTGA	TGTGCACTGGTATGTGGGTGA	Y
NM_001173476	NULL	NULL	SPEG	2	978	TGTCTGTACACAGGCGAGTGA	GCATGCCCAGACCTGGCTT	Y
NM_005876	NULL	NULL	SPEG	2	1026	TACCCTGGCGGCCCCTAG	GAGGGGGTTGGAAGGACC	Y
NM_018650	4139	ENSG00000116141	MARK1	1	2471	TCAAAAATAGCAAATGAGCTTAAGCTGTAA	AATGACACTGTTGGTGAAAATCACCTCAAA	Y
NM_021958	3142	ENSG00000136630	HLX1	1	534	GCGCTTGGCTGCTTATAG	TGTCTGTAACAAGATGGCTCTGAGGT	Y
NM_001201536	9015	ENSG00000143498	TAF1A	1	938	AAATACAGTATTGTAAATCCAAGACTCTGA	GCCAGCTGAAAACAGCCAG	Y
NM_001127366	5077	ENSG00000135903	PAX3	2	1701	TTTCATTATCTCAAGCCAGATATCGCGTAA	ATACAACTGTGGGTTGTGTAACCTTATTTG	Y
NM_005426	7159	ENSG00000143514	TP53BP2	1	1140	CAAAGGAGCTTGGCCTGA	GCAAATGGGTTTGGAAACAATTCACAAA	Y
NM_001618	142	ENSG00000143799	PARP1	1	949	AAATTCAATTTTAAGACCTCCCTGTGGTAA	TCCCATTAAGCAATGCTATTTGCTG	Y
NM_020247	56997	ENSG00000163050	CABC1	1	988	AGGCAGGCCCAGCAGTAG	CCAAGGGCCTCACGCTGAA	Y
NM_001161465	65094	ENSG00000081692	JMJD4	1	1383	GCTGCTGCGGCCCCATAG	CCCCTCCTCCCCGACAAA	Y
NM_033131	89780	ENSG00000154342	WNT3A	1	1986	GTGCACACCTGCAAGTAG	TGCTGACCCACCACCAAACAT	Y
NM_014409	27097	ENSG00000135801	TAF5L	1	1358	GGAATTACACAAGAAAATCAGGAACATTAA	CAGATAAAAATGCACCTTCGTTGGGG	Y
NM_007237	11262	ENSG00000079263	SP140	2	711	GCTATTCAGGAAACAAATGGGAACAATTGA	ACTCAGCAGTTGGGGATGAAAAGA	Y
NM_138402	93349	ENSG00000185404	LOC93349	2	1014	GCTATTCAGGAAACAAATGGGAACAGTTGA	ACTCAGCAGCTGGGGATGAAAA	Y
NM_003113	6672	ENSG00000067066	SP100	2	1069	GAGGAAAATGAAGAAGATGATGATAAATAA	CTCTGGCAAGTACTCTTGAACATAGGTTTC	Y
NM_005381	4691	ENSG00000115053	NCL	2	623	CCACAAGGAAAGAAGACGAAGTTTGAATAG	CACCAGCATTTCAACCAAGTTAGTTAGG	Y
NM_018410	55355	ENSG00000123485	DKFZp762E1312	2	917	CTAGAAAAATTGGAAACTAAAAGTGTGTAG	ACCCAAACTCATATGGCCATATATTAACTA	Y
NM_031371	51742	ENSG00000054267	ARID4B	1	1810	ATGTCAGTTGAGTGCAGGTGA	GATTAATGGACTTTTAATTTTCATAGGTAG	Y
NM_001103	88	ENSG00000077522	ACTN2	1	1819	GGGGAGAGCGATCTGTGA	CACACCCATCCTAAAACACCACAGA	Y
NM_001485	2637	ENSG00000168505	GBX2	2	430	GAACAGGCCCGGCCCTGA	GTTCTTGGGTTTCACGTTTACCTTTGGA	Y
NM_006710	10920	ENSG00000198612	COPS8	2	1284	TATGTGGCTTTCCTTGAAAACTGA	CTCCTGATCCTCTGACTTCTTGAATTC	Y
NM_024101	79083	LRG_83	MLPH	2	1906	GTGGCCCACCAGTCCTAA	GAGAAACCACTGACTTCTGTCAATCTTTAG	Y
NM_001142853	55502	ENSG00000144485	HES6	2	740	GTCTGGAGGCCTTGGTGA	CGGAGTGCATCCCCACCA	Y
NM_001252124	23178	ENSG00000115687	PASK	2	1489	ATCCATACTCGGTTCTCTCTCTGA	GTGTGCATACCTATACACATAACATATACT	Y
NM_006374	10494	ENSG00000115694	STK25	2	825	CTGACATCCACCCGCTGA	GTTCTTCCGGTGGGCCAA	Y
NM_178326	23192	ENSG00000168397	ATG4B	2	1831	TCCTGTTCCTTCTAGATTCTTCTGATGTAG	TGCCAGGGCTGGGGACAC	Y
NM_006352	10472	ENSG00000179456	ZNF238	1	2286	GAAGATAGCTCTCAAGAACTTTGGAAATAA	TAGAATGCTTACACATTTTCCACTCGAGTG	
NM_022366	64216	ENSG00000162851	TFB2M	1	650	CTGTATGATGAAACCCTGGAAGATAGGTAG	TGATAGAACAAATGTGCAGACTTTTCAGTA	Y
NM_003431	7678	ENSG00000196418	ZNF124	1	845	GAAAAGCCCTATAAATGTAAAAAAATGTAA	ACTCAGGAGGCTGAGGCAG	
NM_024836	79894	ENSG00000171161	ZNF672	1	1062	CCAGGGTTCTCTGTGTCCTAG	TTTAGTGTGGAATCAAGTGGTCCTCCA	Y
