# Supplementary material for: A human 3′UTR clone collection to study post-transcriptional gene regulation
Source: BMC Genomics. 2015 Dec 9;16:1036. doi: 10.1186/s12864-015-2238-1 (PMC4673713; doi:10.1186/s12864-015-2238-1)
Supplement: Additional file 2: Table S2. — List of 3′UTRs from the h3′UTRome v1 clone library used in the 3′LIFE assay. Each 3′UTR is referred to by a unique RefSeq ID and its Alias. The 3′UTRs were queried for predicted binding by miR-221 and let-7c using three prediction software; TargetScan, PicTar and DIANA tools [10–12]. Previously validated 3′UTR-miRNA interactions are listed with reference PMIDs. The direct involvement of each gene in the RAS pathway was established by using the GO [54] and KEGG [39] databases. The following column indicates if a given 3′UTR was among the top hits in the 3′LIFE screen for each miRNA. (PDF 37 kb) [file 12864_2015_2238_MOESM2_ESM.pdf]

RefSeq ID	Alias	h3`UTRome Plate	h3`UTRome Well	Predicted?			Validated taget of miR-221/let-7c	Validation References (PMIDs)	RAS pathway	Top hit in the 3`LIFE screen	
				TargetScan [44]	PicTar [45]	DIANA microT [46]				miR-221	let-7c
NM_000076	CDKN1C	R1	A4	miR-221	miR-221	miR-221	miR-221	21461636	-	-	-
NM_001195259	TGFBR3L	R1	C5	-	-	-	-	-	-	-	-
NM_005252	FOS	R1	C11	miR-221	miR-221	miR-221	miR-221	20299489	Y	-	-
NM_001950	E2F4	R1	D10	-	-	-	-	-	-	-	-
NM_024832	RIN3	R1	D11	-	-	-	-	-	Y	-	-
NM_001271007	STRA13	R1	E1	-	-	-	-	-	-	-	-
NM_002467	MYC	R2	C9	-	-	-	-	-	Y	-	-
NM_198256	E2F6	R2	D4	let-7	-	let-7	-	-	-	-	-
NM_001789	CDC25A	R2	D12	let-7	let-7	let-7	let-7c	25909324	-	-	Y
NM_007241	SNF8	R1	E2	-	-	-	-	-	-	-	-
NM_004040	RHOB	R2	E8	-	-	-	-	-	Y	-	Y
NM_001665	RHOG	R2	G6	-	-	-	-	-	Y	-	-
NM_014245	RNF7	R2	H10	let-7	let-7	-	-	-	-	-	Y
NM_002199	IRF2	R3	A9	miR-221	miR-221	miR-221	miR-221	24607843	-	-	-
NM_014212	HOXC11	R3	B1	let-7	let-7	-	-	-	-	-	-
NM_133639	RHOV	R3	B5	-	-	-	-	-	Y	-	-
NM_004310	RHOH	R3	B9	-	-	-	-	-	Y	-	-
NM_001039111	TRIM71	R3	B10	let-7	-	let-7	let-7c	17890240	-	-	Y
NM_005343	HRAS	R3	B12	-	-	-	-	-	Y	-	-
NM_001191	BCL2L1	R3	C10	let-7	let-7	let-7	let-7c	23562878	Y	-	Y
NM_005080	XBP1	R3	D10	-	-	-	-	-	-	Y	-
NM_004958	FRAP1	R3	D11	-	-	-	-	-	-	Y	-
NM_007299	BRCA1	R3	F1	-	-	-	-	-	-	Y	-
NM_002228	JUN	R3	F2	-	-	-	-	-	-	-	-
NM_005225	E2F1	R3	F3	-	-	-	-	-	-	-	-
NM_001126115	TP53	R3	F5	let-7	-	let-7	-	-	-	-	-
NM_198443	NRN1L	R1	E3	-	-	-	-	-	-	-	-
NM_005474	HDAC5	R1	E4	-	-	-	-	-	-	-	-
NM_001014835	PAK4	R4	B6	-	-	-	-	-	Y	-	Y
NM_001654	ARAF	R4	C1	-	-	-	-	-	Y	-	-
NM_000201	ICAM1	R4	E6	-	-	-	miR-221	20110463	-	-	-
NM_004579	MAP4K2	R4	G2	-	-	let-7	-	-	-	-	-
NM_001243926	MAPKAPK3	R4	H6	-	-	-	-	-	Y	-	-
NM_032390	MKI67IP	R5	A12	-	-	-	-	-	-	-	-
NM_004535	MYT1	R5	D12	-	-	-	-	-	-	-	Y
NM_005904	SMAD7	R5	E11	-	-	-	-	-	-	-	-
NM_001143976	WEE1	R5	F9	miR-221	-	-	miR-221	23630541	-	Y	-
NM_152688	KHDRBS2	R5	G4	miR-221	miR-221	miR-221	-	-	-	-	-
NM_002755	MAP2K1	R6	C4	-	-	-	-	-	Y	-	-
NM_005914	MCM4	R6	C10	-	-	-	-	-	-	-	Y
NM_153048	FYN	R6	E6	-	-	-	-	-	-	-	-
NM_003804	RIPK1	R7	A10	-	-	-	-	-	-	-	-
NM_015235	CSTF2T	R7	D7	-	-	-	-	-	-	-	Y
NM_003253	TIAM1	R7	E5	-	-	-	-	-	Y	-	-
NM_032638	GATA2	R1	E5	-	-	-	-	-	-	-	-
NM_015401	HDAC7A	R7	H3	-	-	-	-	-	-	-	-
NM_001145138	RELA	R8	B10	-	-	-	-	-	Y	-	-
NM_005923	MAP3K5	R8	B12	-	-	-	-	-	-	-	-
NM_019884	GSK3A	R8	D1	-	-	-	-	-	-	Y	-
NM_024680	E2F8	R8	D4	-	-	-	-	-	-	-	-
NM_001128167	PAK3	R8	E4	-	-	-	-	-	Y	-	Y
NM_015895	GMNN	R8	G1	-	-	-	-	-	-	Y	Y
NM_001256295	ETS2	R9	A2	miR-221	miR-221	-	miR-221	21715310	Y	Y	-
NM_004556	NFKBIE	R9	D9	-	-	-	-	-	-	-	-
NM_001211	BUB1B	R10	D2	-	-	-	-	-	-	Y	Y
NM_001185081	FMR1	R10	E10	miR-221	-	miR-221	miR-221	23390134	-	Y	-
NM_000222	KIT	R10	F2	miR-221	-	miR-221	miR-221	21119596	Y	Y	Y

NM_005228	EGFR	R10	F12	-	-	-	-	-	Y	-	-
NM_001025243	IRAK1	R10	G11	-	-	-	-	-	-	-	-
NM_002915	RFC3	R10	H2	-	-	-	-	-	-	-	-
NM_021795	ELK4	R11	C3	-	-	let-7	-	-	-	-	-
NM_006343	MERTK	R11	C7	-	-	-	-	-	-	Y	Y
NM_003668	MAPKAPK5	R11	D1	-	-	-	-	-	-	-	-
NM_005391	PDK3	R11	D3	-	-	-	-	-	-	-	Y
NM_001260	CDK8	R11	D5	-	-	-	-	-	-	-	-
NM_001786	CDC2	R11	H2	-	-	-	-	-	-	-	-
NM_004526	MCM2	R12	A1	-	-	-	-	-	-	-	Y
NM_001128620	PAK1	R12	D10	let-7/miR-221	miR-221/let-7	-	miR-221	23333386	Y	-	-
NM_004333	BRAF	R12	F12	-	-	-	-	-	Y	-	Y
NM_020529	NFKBIA	R13	A7	-	-	-	-	-	Y	Y	Y
NM_002229	JUNB	R13	A8	-	-	-	-	-	-	-	-
NM_002913	RFC1	R13	H7	-	-	-	-	-	-	-	-
NM_002908	REL	R14	C3	-	-	-	-	-	Y	-	-
NM_002880	RAF1	R14	E5	-	-	-	-	-	Y	-	-
NM_012115	CASP8AP2	R14	E10	-	-	-	-	-	-	-	-
NM_013254	TBK1	R14	H10	-	-	-	-	-	Y	-	-
NM_003954	MAP3K14	R15	E5	-	-	-	-	-	-	-	-
NM_004052	BNIP3	R15	G9	-	-	-	-	-	-	-	-
NM_003106	SOX2	R2	F2	-	-	-	-	-	-	-	-
NM_000346	SOX9	R2	H1	-	-	-	-	-	-	Y	-
NM_006914	RORB	R1	E6	-	-	miR-221	-	-	-	-	Y
NM_005634	SOX3	R3	E3	-	-	-	-	-	-	-	-
NM_004189	SOX14	R4	B7	-	-	-	-	-	-	-	-
NM_018419	SOX18	R4	C3	-	-	-	-	-	-	-	-
NM_031439	SOX7	R9	A6	-	-	-	-	-	-	-	-
NM_001363	DKC1	R9	E10	-	-	-	-	-	-	-	-
NM_022454	SOX17	R11	H1	-	-	-	-	-	-	-	-
