# Supplementary material for: A human 3′UTR clone collection to study post-transcriptional gene regulation
Source: BMC Genomics. 2015 Dec 9;16:1036. doi: 10.1186/s12864-015-2238-1 (PMC4673713; doi:10.1186/s12864-015-2238-1)
Supplement: Additional file 3: Table S3. — Gene Ontology analysis of let-7c and miR-221 top hits. The top hits for let-7c and miR-221 were queried for enrichments in biological processes. Only results with p < 0.05 are shown. The resulting biological processes are sorted based on fold enrichment. (PDF 57 kb) [file 12864_2015_2238_MOESM3_ESM.pdf]

Analysis Type: PANTHER Overrepresentation Test (release 20150430)
Annotation Version and Release Date: GO Ontology database Released 2015-08-06
Reference List: Homo sapiens (all genes in database)
Bonferroni correction: TRUE
Results with P<0.05

let-7c

Number of *let-7c* top hits = 19

GO biological process	Total number of genes in pathway	Number of let-7c top hits in pathway	Expected number of hits	Fold Enrichment	P-value
cell cycle checkpoint	270	5	0.25	> 5	2.85E-02
mitotic cell cycle	791	7	0.72	> 5	2.99E-02
cell cycle	1322	9	1.21	> 5	6.72E-03

---

miR-221

Number of *miR-221* top hits = 13

GO biological process	Total number of genes in pathway	Number of let-7c top hits in pathway	Expected number of hits	Fold Enrichment	P-value
negative regulation of muscle cell differentiation	54	3	0.03	> 5	2.94E-02
negative regulation of programmed cell death	846	6	0.49	> 5	2.62E-02
negative regulation of cell death	904	6	0.52	> 5	3.84E-02
response to organonitrogen compound	913	6	0.53	> 5	4.07E-02
cellular response to oxygen-containing compound	924	6	0.53	> 5	4.36E-02

negative regulation of cellular macromolecule biosynthetic process	1240	8	0.71	> 5	4.91E-04
protein phosphorylation	944	6	0.54	> 5	4.93E-02
negative regulation of macromolecule biosynthetic process	1318	8	0.76	> 5	7.89E-04
negative regulation of cellular biosynthetic process	1381	8	0.8	> 5	1.13E-03
negative regulation of biosynthetic process	1401	8	0.81	> 5	1.27E-03
negative regulation of nitrogen compound metabolic process	1406	8	0.81	> 5	1.30E-03
negative regulation of nucleobase-containing compound metabolic process	1310	7	0.76	> 5	1.82E-02
positive regulation of nucleic acid-templated transcription	1403	7	0.81	> 5	2.88E-02
positive regulation of transcription, DNA-templated	1403	7	0.81	> 5	2.88E-02
negative regulation of gene expression	1404	7	0.81	> 5	2.89E-02
regulation of programmed cell death	1424	7	0.82	> 5	3.18E-02
positive regulation of RNA biosynthetic process	1431	7	0.83	> 5	3.28E-02
positive regulation of RNA metabolic process	1467	7	0.85	> 5	3.87E-02
regulation of cell death	1508	7	0.87	> 5	4.65E-02
intracellular signal transduction	1816	8	1.05	> 5	9.36E-03
negative regulation of macromolecule metabolic process	2207	9	1.27	> 5	2.15E-03

negative regulation of cellular metabolic process	2213	9	1.28	> 5	2.20E-03
negative regulation of metabolic process	2481	9	1.43	> 5	5.92E-03
negative regulation of cellular process	4025	11	2.32	4.74	1.09E-03
negative regulation of biological process	4372	11	2.52	4.36	2.65E-03
